# Supplementary material for: Synthesis, Antiproliferative Evaluation and QSAR Analysis of Novel Halogen- and Amidino-Substituted Benzothiazoles and Benzimidazoles
Source: Int J Mol Sci. 2022 Dec 13;23(24):15843. doi: 10.3390/ijms232415843 (PMC9785280; doi:10.3390/ijms232415843)

Supplementary Material

Synthesis, antiproliferative evaluation and QSAR analysis of novel halogen- and amidino-substituted benzothiazoles and benzimidazoles

Valentina Rep Kaulić[1], Livio Racane[2], Marijana Leventić[3], Domagoj Šubarić[4], Vesna Rastija[4], Ljubica Glavaš-Obrovac[3*], Silvana Raić-Malić[1*]

[1] Department of Organic Chemistry, Faculty of Chemical Engineering and Technology, University of Zagreb, Marulićev trg 20, 10000 Zagreb, Croatia; vrep@fkit.hr

[2] Department of Applied Chemistry, Faculty of Textile Technology, University of Zagreb, Prilaz baruna Filipovića 28; 10000 Zagreb, Croatia; livio.racane@ttf.unizg.hr

[3] Department of Medicinal Chemistry, Biochemistry and Laboratory Medicine, Faculty of Medicine Osijek, University Josip Juraj Strossmayer of Osijek, Josipa Huttlera 4, 31000 Osijek, Croatia; lgobrovac@mefos.hr

[4] Faculty of Agrobiotechnical Sciences Osijek, Josip Juraj Strossmayer University of Osijek, Vladimira Preloga 1, 31000 Osijek, Croatia; vrastija@fazos.hr

**Content**	**Pages**
In vitro growth-inhibitory effects of novel compounds with calculated selectivity index	S2-S4
Experimental and calculated $\log IC_{50}$	S5–S9
^{1}H and ^{13}C NMR spectra of novel compounds	S10–S74

Table S1. *In vitro* growth-inhibitory effects of compounds **15a–26a, 15b–26b,** and **15c–26c** on selected tumor cell lines with calculated selectivity index.

Cpd	IC_{50} (µM)[1]										
	MDCK1	BJ	HeLa	SI (MDCK)	SI (BJ)	CaCo-2	SI (MDCK)	SI (BJ)	HuT78	SI (MDCK)	SI (BJ)
**15a**	>100	>100	>100	-	-	>100	-	-	>100	-	-
**15b**	>100	>100	>100	-	-	>100	-	-	>100	-	-
**15c**	79.3 ± 14.4	100	>100	-	-	>100	-	-	>100	-	-
**16a**	87.3 ± 6.9	>100	>100	-	-	>100	-	-	>100	-	-
**16b**	25.8 ± 9.4	>100	>100	-	-	89.3 ± 15.8	-	-	69.8 ± 15.5	-	1.43
**16c**	6.8 ± 6.2	>100	>100	-	-	>100	-	-	>100	-	-
**17a**	100	>100	>100	-	-	>100	-	-	>100	-	-
**17b**	>100	>100	>100	-	-	>100	-	-	>100	-	-
**17c**	78.0 ± 27.8	>100	>100	-	-	>100	-	-	>100	-	-
**18a**	17.6 ± 5.7	>100	42.4 ± 7.5	-	-	100	-	-	13.2 ± 1.2	-	3.2
**18b**	29.8 ± 17.7	>100	>100	-	-	>100	-	-	100	-	-
**18c**	41.4 ± 8.1	>100	67.3 ± 7.8	-	-	>100	-	-	55.6 ± 34.6	-	
**19a**	1.1 ± 0.2	>100	>100	-	-	>100	-	-	>100	-	-
**19b**	16.3 ± 11.5	>100	>100	-	-	>100	-	-	100	-	-
**19c**	1.1 ± 0.2	>100	>100	-	-	>100	-	-	>100	-	-
**20a**	65.7 ± 11.8	>100	>100	-	-	>100	-	-	>100	-	-
**20b**	94.2 ± 18.4	>100	>100	-	-	28.7 ± 16.7	3.28	3.48	>100	-	-
**20c**	85.9 ± 15.7	100	>100	-	-	89.2 ± 16.8	-	1.12	64.7 ± 11.3	1.33	1.55
**21a**	12.6 ±4.8	>100	100	-	-	>100	-	-	6.8 ± 6.2	1.85	14.7
**21b**	10.1 ±4.5	84.1 ±24.7	100	-	-	>100	-	-	3.6 ± 3.1	2.8	23.36
**21c**	43.2 ±12.4	>100	100	-	-	>100	-	-	24.3 ± 6.5	1.78	4.11
**22a**	1.1 ± 0.2	>100	54.5 ± 29.4	-	-	73.5 ±4.5	-	-	14.9 ± 10.8	-	6.71
**22b**	1.0 ± 0.0	86.4 ± 20.9	50.6 ± 19.3	-		100	-	-	9.1 ± 2.8	-	9.49
**22c**	0.7 ± 0.3	>100	100	-	-	>100	-	-	38.1 ± 23.4	-	2.62
**23a**	40.7 ± 5.7	>100	52.4 ± 18.4	-		95.6 ± 6.6	-	-	19.0 ± 4.0	2.14	5.26
**23b**	27.4 ± 4.2	40.5 ± 17.2	34.9 ± 14.8	-	-	56.0 ± 14.6	-	-	17.1 ± 7.7	1.6	2.34
**23c**	29.5 ± 7.2	100	45.7 ± 16.3	-		96.4 ± 13.3	-	-	20.0 ± 8.7	-	5.0
**24a**	11.4 ± 14.5	>100	>100	-	-	>100	-	-	>100	-	-
**24b**	8.8 ± 8.3	>100	>100	-	-	>100	-	-	>100	-	-
**24c**	41.1 ± 17.2	>100	82.8 ± 32.3	-	-	>100	-	-	49.6 ± 38.0	-	2.0
**25a**	8.2 ± 5.8	10 ± 0.01	>100	-	-	>100	-	-	>100	-	-
**25b**	51.3 ± 4.6	>100	>100	-	-	>100	-	-	19.7 ± 10.6	2.6	5.07
**25c**	22.8 ± 4.9	>100	>100	-	-	77.6 ±43.3	-	-	>100	-	-
**26a**	36.7 ± 18.0	>100	>100	-	-	>100	-	-	>100	-	-
**26b**	64.8 ± 38.5	>100	>100	-	-	>100	-	-	>100	-	-
**26c**	8.5 ± 6.4	76.7 ± 27.7	23.3 ± 15.3	-		100	-	-	23.7 ± 5.2	-	3.24
**5-FU**	55.0± 8.7	74.0± 3.1	8.2±1.9	6.7	9.02	5.9±0.7	9.32	7.94	>100	-	-

[1] IC_{50} – Compound concentration that inhibited cell growth by 50 %. Data represents mean IC_{50} (µM) values ± standard deviation (SD) of three independent experiments. Exponentially growing cells were treated with compounds during 72 hrs. Cytotoxicity was analysed using MTT survival assay. **5-FU**: 5-Fluorouracil. SI = IC_{50} normal cell line (MDCK1 or BJ)/IC_{50} for respective cancerous cell line.

Table S2. *In vitro* growth-inhibitory effects of benzothiazoles **34a**–**38a**, **40a**, **41a**, **34b**, **36b**–**38b**, **40b**, **34c**–**37c**, **39**c–**41c** and benzimidazoles **42a**–**49a**, **42b**–**45b**, **47b**–**49b**, **42c–46c**, **48c**, and **49c** on selected tumor and normal cell lines with calculated selectivity index.

Cpd	IC_{50} (μM)[1]										
	MDCK1	BJ	HeLa	SI (MDCK)	SI (BJ)	CaCo-2	SI (MDCK)	SI (BJ)	HuT78	SI (MDCK)	SI (BJ)
**34a**	2.8 ± 0.3	3.9 ± 0.3	3.4 ± 0.2	-	-	4.0 ± 0.2	-	-	2.2 ± 0.3	-	-
**34b**	2.2 ± 0.3	11.2 ± 6.0	4.5 ± 4.3	-	-	7.0 ± 5.4	-	-	2.1 ± 0.2	-	-
**34c**	2.1 ± 0.3	4.0 ± 0.1	3.1 ± 0.2	-	-	6.3 ± 3.0	-	-	1.8 ± 0.7	-	-
**35a**	2.7 ± 0.3	3.2 ± 0.3	18.9 ± 3.6	-	-	26.7 ± 2.1	-	-	1.4 ± 0.2	-	-
**35c**	7.5 ± 3.5	6.7 ± 4.9	21.8 ± 5.5	-	-	18.2 ± 3.3	-	-	6.2 ± 2.7	-	-
**36a**	31.8 ± 4.0	38.5 ± 4.6	62.6 ± 22.0	-	-	24.8 ± 9.2	-	-	4.4 ± 3.9	7.23	8.75
**36b**	25.2 ± 1.3	32.6 ± 3.4	30.0 ± 1.0	-	-	38.3 ± 1.2	-	-	1.8 ± 0.4	14	18.11
**36c**	100	>100	>100	-	-	>100	-	-	1.6 ± 0.8	62.5	62.5
**37a**	1.5 ± 0.3	4.3 ± 3.6	3.6 ± 0.1	-	-	3.7 ± 1.5	-	-	1.6 ± 0.8	-	2.69
**37b**	2.4 ± 0.3	5.3 ± 2.9	4.0 ± 0.4	-	-	6.1 ± 2.7	-	-	2.1 ± 0.2	-	2.52
**37c**	2.6 ± 0.1	4.8 ± 3.5	3.4 ± 0.2	-	-	4.5 ± 2.7	-	-	3.0 ± 1.0	-	1.6
**38a**	27.1 ± 3.4	22.6 ± 4.6	19.7 ± 3.1	-	-	25.4 ± 3.5	-	-	16.2 ±5.5	1.67	1.39
**38b**	26.6 ± 3.1	32.2 ± 2.8	22.1 ± 1.2	-	-	19.4 ± 4.7	1.37	1.66	4.0 ±0.9	6.65	8.05
**39c**	100	38.1 ±1.4	>100	-	-	34.7 ±2.8	2.87	-	12.7 ±0.9	7.87	3
**40a**	59.4 ± 18.8	>100	100	-	-	100	-	-	100	-	-
**40b**	>100	>100	>100	-	-	>100	-	-	37.3 ± 12.8	2.68	2.68
**40c**	>100	100	>100	-	-	>100	-	-	100	-	-
**41a**	58.2 ±17.3	27.3 ±6.0	25.8 ±0.8	-	-	29.2 ±4.2	1.99	-	4.1 ±3.6	14.19	6.65
**41c**	>100	>100	>100	-	-	>100	-	-	>100	-	-
**42a**	100	100	>100	-	-	>100	-	-	7.0 ± 5.2	14.29	14.29
**42b**	67.3 ± 12.7	47.0 ± 3.3	100	-	-	100	-	-	11.9 ± 3.6	5.66	3.95
**42c**	100	32.8 ± 4.1	>100	-	-	>100	-	-	24.9 ± 4.6	4.0	1.32
**43a**	100	33.0 ±20.3	>100	-		>100	-	-	28.6 ± 3.9	3.5	-
**43b**	100	>100	>100	-	-	>100	-	-	43.3 ± 4.4	2.3	2.3
**43c**	63.4 ± 8.9	30.9 ± 8.7	>100	-		>100	-	-	17.7 ± 6.1	3.58	1.75
**44a**	100	73.3 ± 3.2	100	-	-	>100	-	-	89.2 ± 20.6	1.2	-
**44b**	>100	45.1 ± 20.9	>100	-		>100	-	-	65.6 ± 17.3	1.52	-
**44c**	100	>100	>100	-	-	>100	-	-	100	-	-
**45a**	100	55.6 ± 13.4	>100	-	-	100	-	-	4.8 ± 3.6	20.83	11.58
**45b**	46.1 ± 14.8	82.1 ± 0.4	>100	-	-	100	-	-	5.5 ± 4.0	8.36	14.9
**45c**	68.5 ± 22.7	>100	>100	-	-	>100	-	-	4.1 ± 3.3	16.7	24.39
**46a**	100	100	>100	-	-	>100	-	-	63.0 ± 15.1	1.59	1.59
**46c**	>100	>100	>100	-	-	>100	-	-	5.1 ± 4.2	19.6	19.6
**47a**	100	>100	>100	-	-	>100	-	-	100	-	-
**47b**	>100	100	>100	-	-	>100	-	-	100	-	-
**48a**	100	>100	>100	-		>100	-	-	100	-	-
**48b**	>100	100	>100	-	-	>100	-	-	100	-	-
**48c**	>100	100	>100	-	-	>100	-	-	69.6 ± 25.9	1.44	1.44
**49a**	100	80.4 ± 9.9	100	-	-	>100	-	-	100	-	-
**49b**	82.9 ± 6.6	64.8 ± 9.2	100	-	-	>100	-	-	100	-	-
**49c**	>100	>100	100	-	-	>100	-	-	100	-	-
**5-FU**	55.0± 8.7	74.0± 3.1	8.2±1.9	6.7	9.02	5.9±0.7	9.32	7.94	>100	-	-

[1] IC_{50} – Compound concentration that inhibited cell growth by 50 %. Data represents mean IC_{50} (μM) values ± standard deviation (SD) of three independent experiments. Exponentially growing cells were treated with compounds during 72 hrs. Cytotoxicity was analysed using MTT survival assay. **5-FU**: 5-Fluorouracil. SI = IC_{50} normal cell line (MDCK1 or BJ)/IC_{50} for respective cancerous cell line.

Table S3. Additional antiproliferative testing of selected compounds with calculated selectivity index.

Cpd	IC_{50} (µM)[1]													
	**MDCK1**	**BJ**	**SW620**	**SI (MDCK)**	**SI (BJ)**	**MDA-MB-231**	**SI (MDCK)**	**SI (BJ)**	**HL60**	**SI (MDCK)**	**SI (BJ)**	**THP1**	**SI (MDCK)**	**SI (BJ)**
**36a**	2.7 ± 0.3	3.2 ± 0.3	5.6 ± 1.2	-	-	33.0 ± 4.9	-	-	18.8 ± 1.1	-	-	20.4 ± 3.3	-	-
**36c**	100	>100	100	-	-	>100	-	-	100	-	-	>100	-	-
**38b**	26.6 ± 3.1	32.2 ± 2.8	30.5 ± 9.2	-	-	42.4 ± 7.2	-	-	15.8 ± 2.3	1.68	2.04	14.7 ± 4.1	1.8	2.19
**39c**	100	38.1 ±1.4	34.2 ± 7.1	2.92	-	38.5 ± 1.6	2.6	-	31.1 ± 3.0	3.22	-	69.7 ± 6.3	1.43	-
**42a**	100	100	100	-	-	76.3 ± 11.8	1.31	1.31	33.2 ± 1.8	3.01	3.01	44.5 ± 5.3	2.25	2.25
**42b**	67.3 ± 12.7	47.0 ± 3.3	92.7 ± 34.3	-	-	84.5 ± 18.3	-	-	44.4 ± 12.6	1.51	-	100	-	-
**42c**	100	32.8 ± 4.1	29.8 ± 3.0	3.36	-	38.9 ± 4.2	2.57	-	19.5 ± 0.8	5.13	1.68	19.7 ± 2.4	5.13	1.68
**43a**	100	33.0 ±20.3	>100	-	-	100	-	-	40.4 ± 12.0	2.48	-	29.7 ± 8.6	3.37	-
**45a**	100	55.6 ± 13.4	>100	-	-	100	-	-	48.1 ± 16.4	2.07	1.15	0.8 ± 0.1	125	69,5
**45b**	46.1 ± 14.8	82.1 ± 0.4	100	-	-	100	-	-	30.0 ± 12.3	1.54	2.74	2.5 ± 1.5	18.44	32.84
**45c**	68.5 ± 22.7	>100	100	-	-	100	-	-	25.1 ± 16.0	2.73	4.0	>100	-	-
**46c**	>100	>100	>100	-	-	>100	-	-	50.7 ± 20.5	1.97	1.97	100	-	-

[1] IC_{50} – Compound concentration that inhibited cell growth by 50 %. Data represents mean IC_{50} (µM) values ± standard deviation (SD) of three independent experiments. Exponentially growing cells were treated with compounds during 72 hrs. Cytotoxicity was analysed using MTT survival assay. **5-FU**: 5-Fluorouracil. SI = IC_{50} normal cell line (MDCK1 or BJ)/ IC_{50} for respective cancerous cell line.

Table S4. Experimental and calculated l $logIC_{50}$ by Eqs. (1 and 1a), as well as values of descriptors included in QSAR model (Eq. 1 and 1a) antiproliferative activity against MDCK-1 cells.

Code	Status	Exp. endpoint	Pred. eq.(1)	Pred. eq.(1a)	*SIC1*	*GATS4p*	*BEHv6*	*BELp1*	*R7m*
**15a**	Training	2.00	1.96	1.96	0.577	1.241	2.725	1.954	0.199
**15b**	Training	2.00	2.00	1.97	0.624	1.201	2.725	1.955	0.249
**15c**	Training	1.89	1.87	1.83	0.61	1.216	2.844	1.963	0.245
**16a**	Training	1.91	1.31	1.29	0.577	1.498	2.715	1.955	0.182
**16b**	Training	1.41	1.57	1.53	0.605	1.421	2.715	1.955	0.232
**16c**	Training	0.83	1.34	1.29	0.61	1.421	2.842	1.964	0.232
**17a**	Prediction	2.00	2.10	2.12	0.529	1.271	2.713	1.955	0.162
**17b**	Training	2.00	2.11	2.10	0.577	1.238	2.713	1.955	0.214
**17c**	Prediction	1.89	1.99	1.96	0.569	1.25	2.842	1.964	0.219
**18a**	Training	1.25	1.40	1.41	0.609	1.194	2.724	1.957	0.126
**18b**	Prediction	1.47	1.37	1.36	0.659	1.177	2.724	1.958	0.179
**18c**	Training	1.62	1.40	1.37	0.639	1.187	2.843	1.967	0.187
**19a**	Training	0.04	0.62	0.61	0.609	1.516	2.714	1.958	0.112
**19b**	Training	1.21	0.90	0.88	0.638	1.436	2.714	1.958	0.166
**19c**	Training	0.04	0.78	0.74	0.639	1.43	2.842	1.967	0.176
**20a**	Prediction	1.82	1.61	1.65	0.559	1.218	2.713	1.958	0.097
**20b**	Training	1.97	1.55	1.56	0.609	1.214	2.713	1.958	0.151
**20c**	Training	1.93	1.54	1.53	0.595	1.22	2.842	1.967	0.164
**21a**	Training	1.10	1.08	1.08	0.736	0.795	2.724	1.939	0.139
**21b**	Training	1.00	0.90	0.85	0.785	0.907	2.724	1.94	0.212
**21c**	Training	1.64	1.35	1.32	0.745	0.887	2.758	1.953	0.208
**22a**	Training	0.04	0.13	0.10	0.736	1.199	2.713	1.941	0.124
**22b**	Training	0.00	0.35	0.29	0.762	1.217	2.713	1.942	0.198
**22c**	Prediction	-0.15	0.62	0.57	0.745	1.189	2.754	1.954	0.197
**23a**	Training	1.61	1.36	1.38	0.685	0.8	2.711	1.941	0.108
**23b**	Training	1.44	1.11	1.09	0.736	0.935	2.711	1.942	0.182
**23c**	Training	1.47	1.53	1.52	0.703	0.909	2.753	1.954	0.185
**24a**	Prediction	1.06	1.39	1.37	0.62	0.968	2.948	1.952	0.159
**24b**	Training	0.94	1.39	1.33	0.659	1.023	2.948	1.952	0.23
**24c**	Prediction	1.61	1.55	1.50	0.638	1.027	2.948	1.956	0.22
**25a**	Prediction	0.91	0.57	0.52	0.62	1.287	2.946	1.952	0.143

**25b**	Training	1.71	0.89	-	0.643	1.275	2.946	1.952	0.221
**25c**	Training	1.36	0.96	0.90	0.638	1.273	2.947	1.957	0.213
**26a**	Training	1.56	1.49	1.49	0.581	0.978	2.946	1.952	0.127
**26b**	Training	1.81	1.50	1.46	0.62	1.047	2.946	1.952	0.205
**26c**	Training	0.93	1.72	1.69	0.603	1.049	2.947	1.957	0.208
**34a**	Training	0.45	0.76	0.71	0.609	1.221	3.085	1.968	0.154
**34b**	Training	0.34	0.77	0.71	0.644	1.201	3.085	1.969	0.192
**34c**	Prediction	0.32	0.93	0.87	0.622	1.218	3.091	1.972	0.19
**35a**	Training	0.43	0.82	0.81	0.637	1.178	2.934	1.969	0.121
**35c**	Training	0.88	1.11	1.08	0.646	1.191	2.935	1.974	0.172
**36a**	Training	1.50	1.19	1.21	0.717	0.869	2.766	1.967	0.109
**36b**	Prediction	1.40	1.26	1.23	0.753	0.955	2.766	1.967	0.198
**36c**	Training	2.00	1.51	1.48	0.715	0.946	2.844	1.969	0.213
**37a**	Prediction	0.18	0.75	0.71	0.636	1.016	3.127	1.967	0.132
**37b**	Training	0.38	0.70	0.63	0.666	1.058	3.128	1.967	0.179
**37c**	Prediction	0.41	0.91	0.85	0.643	1.067	3.134	1.97	0.181
**38a**	Training	1.43	1.56	1.54	0.609	1.229	3.113	2.02	0.173
**38b**	Training	1.42	1.46	1.43	0.641	1.208	3.113	2.02	0.191
**39c**	Training	2.00	1.88	1.88	0.64	1.199	2.946	2.021	0.183
**40a**	Training	1.77	2.02	2.07	0.707	0.873	2.826	2.02	0.128
**40b**	Training	2.00	1.98	1.99	0.74	0.959	2.826	2.02	0.196
**40c**	Training	2.00	2.28	2.29	0.703	0.951	2.861	2.021	0.205
**41a**	Prediction	1.76	1.46	1.46	0.632	1.021	3.169	2.02	0.134
**41c**	Training	2.00	1.64	1.62	0.638	1.073	3.175	2.021	0.19
**42a**	Training	2.00	1.58	1.57	0.609	1.328	3.111	2.039	0.166
**42b**	Training	1.83	1.54	1.50	0.641	1.323	3.112	2.039	0.199
**42c**	Training	2.00	1.69	1.66	0.618	1.359	3.115	2.04	0.205
**43a**	Prediction	2.00	1.67	1.70	0.633	1.256	2.946	2.039	0.118
**43b**	Training	2.00	1.52	1.53	0.666	1.291	2.946	2.039	0.151
**43c**	Training	1.80	1.95	1.94	0.64	1.327	2.946	2.04	0.195
**44a**	Training	2.00	2.34	2.41	0.703	0.854	2.826	2.039	0.123
**44b**	Training	2.00	2.15	2.17	0.735	1.025	2.826	2.039	0.196
**44c**	Training	2.00	2.39	2.41	0.699	1.022	2.861	2.04	0.198
**45a**	Training	2.00	1.77	1.78	0.632	1.043	3.166	2.039	0.145

**45b**	Training	1.66	1.52	1.50	0.66	1.131	3.166	2.039	0.173
**45c**	Training	1.84	1.71	1.69	0.637	1.152	3.172	2.04	0.181
**46a**	Training	2.00	2.25	2.26	0.642	0.899	3.167	2.039	0.183
**46c**	Training	2.00	2.09	2.07	0.637	1.023	3.173	2.04	0.195
**47a**	Prediction	2.00	1.77	1.77	0.639	1.18	3.047	2.039	0.155
**47b**	Prediction	2.00	1.92	1.89	0.664	1.232	3.047	2.039	0.231
**48a**	Training	2.00	1.97	1.99	0.618	1.122	3.038	2.039	0.135
**48b**	Training	2.00	1.98	1.97	0.642	1.201	3.038	2.04	0.194
**48c**	Training	2.00	2.23	2.23	0.614	1.211	3.039	2.04	0.202
**49a**	Training	2.00	1.90	1.94	0.601	1.222	2.984	2.039	0.117
**49b**	Training	1.92	1.84	1.85	0.625	1.259	2.984	2.039	0.151
**49c**	Training	2.00	2.17	2.18	0.597	1.297	2.985	2.04	0.18

Table S5. Experimental and calculated l $logIC_{50}$ by Eqs. (1 and 1a), as well as values of descriptors included in QSAR model (Eq. 2 and 2a) for antiproliferative activity on T-cell lymphoma (HuT78).

Code	Status	Exp. endpoint	Pred. eq.(2)	Pred. eq.(2a)	*MATS8v*	*Mor30m*	*Mor09p*	*E2u*
**15c**	Training	2	1.52	1.6	-0.33	0.469	-1.288	0.36
**16b**	Training	1.84	1.47	1.45	-0.289	0.087	-1.498	0.484
**18a**	Training	1.12	1.31	1.41	-0.134	0.215	-1.039	0.5
**18b**	Training	2	1.63	1.65	-0.255	0.21	-1.075	0.512
**18c**	Training	1.75	1.57	1.69	-0.183	0.297	-1.006	0.443
**19b**	Prediction	2	1.99	1.96	-0.343	0.082	-1.229	0.511
**20c**	Training	1.81	1.82	1.93	-0.209	0.154	-1.123	0.44
**21a**	Training	0.83	0.82	0.82	-0.258	0.489	-1.144	0.516
**21b**	Training	0.56	0.83	0.77	-0.329	0.517	-1.226	0.525
**21c**	Training	1.39	0.97	1.01	-0.312	0.633	-1.172	0.428
**22a**	Training	1.17	1.45	1.37	-0.407	0.347	-1.29	0.513
**22b**	Prediction	0.96	1.52	1.4	-0.461	0.331	-1.352	0.524
**22c**	Training	1.58	1.38	1.37	-0.401	0.505	-1.298	0.427
**23a**	Training	1.28	1.2	1.16	-0.331	0.374	-1.243	0.514
**23b**	Prediction	1.23	1.33	1.24	-0.407	0.371	-1.307	0.521
**23c**	Prediction	1.3	1.35	1.35	-0.369	0.515	-1.245	0.425
**24c**	Training	1.7	1.7	1.76	-0.312	0.232	-1.5	0.36
**25b**	Training	1.29	0.58	-	-0.374	0.478	-1.667	0.47
**26c**	Training	1.37	1.13	1.17	-0.344	0.548	-1.473	0.348
**34a**	Training	0.34	0.08	0.08	-0.142	0.414	-1.473	0.515
**34b**	Prediction	0.34	0.11	0.05	-0.254	0.434	-1.771	0.47
**34c**	Training	0.26	0.2	0.26	-0.195	0.533	-1.681	0.376
**35a**	Training	0.15	0.5	0.49	-0.203	0.368	-1.466	0.495
**35c**	Training	0.79	0.95	1.04	-0.22	0.424	-1.42	0.383
**36a**	Training	0.64	0.76	0.7	-0.301	0.364	-1.502	0.5
**36b**	Training	0.26	0.62	0.53	-0.338	0.472	-1.488	0.506
**36c**	Training	0.2	0.76	0.75	-0.329	0.534	-1.518	0.415
**37a**	Prediction	0.2	0.51	0.43	-0.281	0.262	-1.836	0.473
**37b**	Training	0.32	0.3	0.19	-0.333	0.383	-1.892	0.472
**37c**	Training	0.48	0.71	0.72	-0.33	0.56	-1.647	0.362
**38a**	Training	1.21	0.92	0.98	-0.154	0.23	-1.331	0.486
**38b**	Training	0.6	0.73	0.75	-0.254	0.483	-1.41	0.435
**39c**	Training	1.1	1.77	-	-0.227	0.41	-0.9	0.404
**40a**	Training	2	2.08	2.09	-0.304	0.067	-1.11	0.513
**40b**	Training	1.57	1.86	1.84	-0.332	0.151	-1.188	0.509
**40c**	Training	2	1.93	1.99	-0.327	0.313	-1.097	0.427
**41a**	Prediction	0.61	0.77	0.75	-0.286	0.412	-1.494	0.459
**42a**	Training	0.85	0.86	0.87	-0.183	0.14	-1.553	0.49
**42b**	Training	1.08	1.39	1.37	-0.29	-0.003	-1.817	0.43

**42c**	Prediction	1.4	1.91	2.01	-0.225	-0.004	-1.517	0.371
**43a**	Training	1.46	1.36	1.37	-0.26	0.092	-1.558	0.458
**43b**	Prediction	1.64	1.77	1.73	-0.332	-0.048	-1.644	0.467
**43c**	Training	1.25	1.2	1.23	-0.259	0.184	-1.655	0.405
**44a**	Training	1.95	2.03	1.99	-0.364	-0.049	-1.567	0.462
**44b**	Training	1.82	1.72	1.66	-0.387	0.069	-1.61	0.468
**44c**	Training	2	1.71	1.7	-0.374	0.149	-1.628	0.405
**45a**	Training	0.68	0.79	0.71	-0.327	0.115	-2.131	0.413
**45b**	Training	0.74	0.78	0.66	-0.372	0.152	-2.114	0.437
**45c**	Training	0.61	1.03	0.98	-0.367	0.271	-1.871	0.397
**46a**	Training	1.8	1.4	1.43	-0.218	0.077	-1.44	0.47
**46c**	Prediction	0.71	1.12	1.02	-0.286	-0.027	-1.769	0.527
**47a**	Training	2	2.08	2.13	-0.261	0.051	-1.082	0.497
**47b**	Prediction	2	2.69	-	-0.363	-0.032	-1.096	0.478
**48a**	Training	2	1.92	1.97	-0.271	0.071	-1.299	0.447
**48b**	Training	2	1.8	1.76	-0.372	0.154	-1.4	0.471
**48c**	Prediction	1.84	2.24	2.29	-0.292	0.071	-1.092	0.47
**49a**	Training	2	2.22	2.32	-0.24	0.03	-1.124	0.442
**49b**	Training	2	2.18	2.18	-0.354	0.051	-1.312	0.459
**49c**	Training	2	1.83	1.85	-0.277	0.104	-1.229	0.483

Fig. S1 a) ^{1}H NMR and b) ^{13}C NMR of compd. **9a**

a)

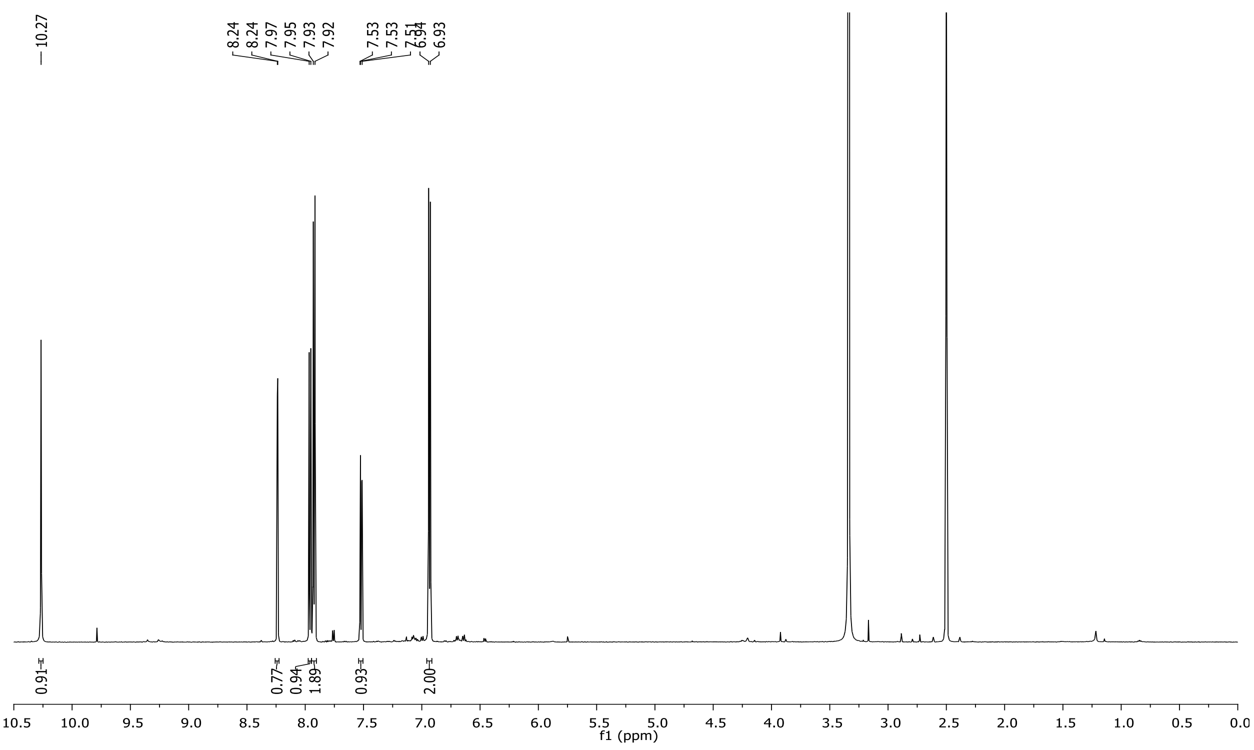

b)

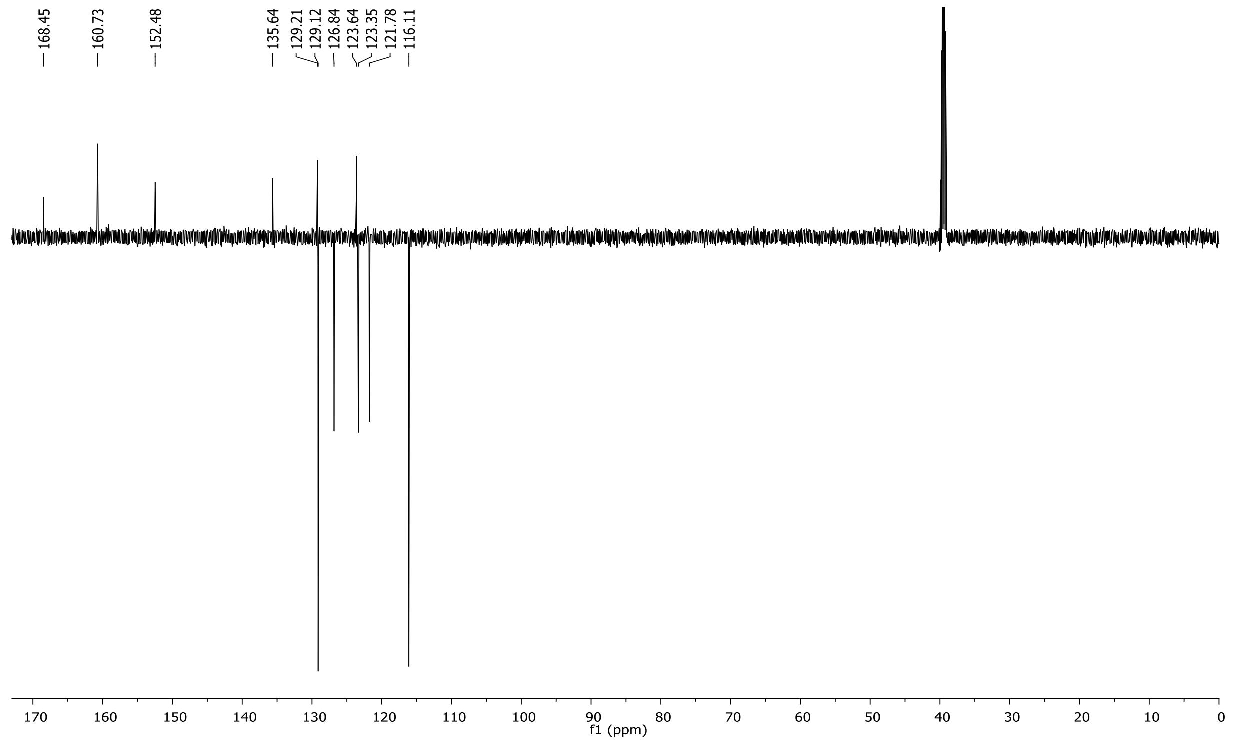

Fig. S2 a) ^{1}H NMR and b) ^{13}C NMR of compd. **9b**

a)

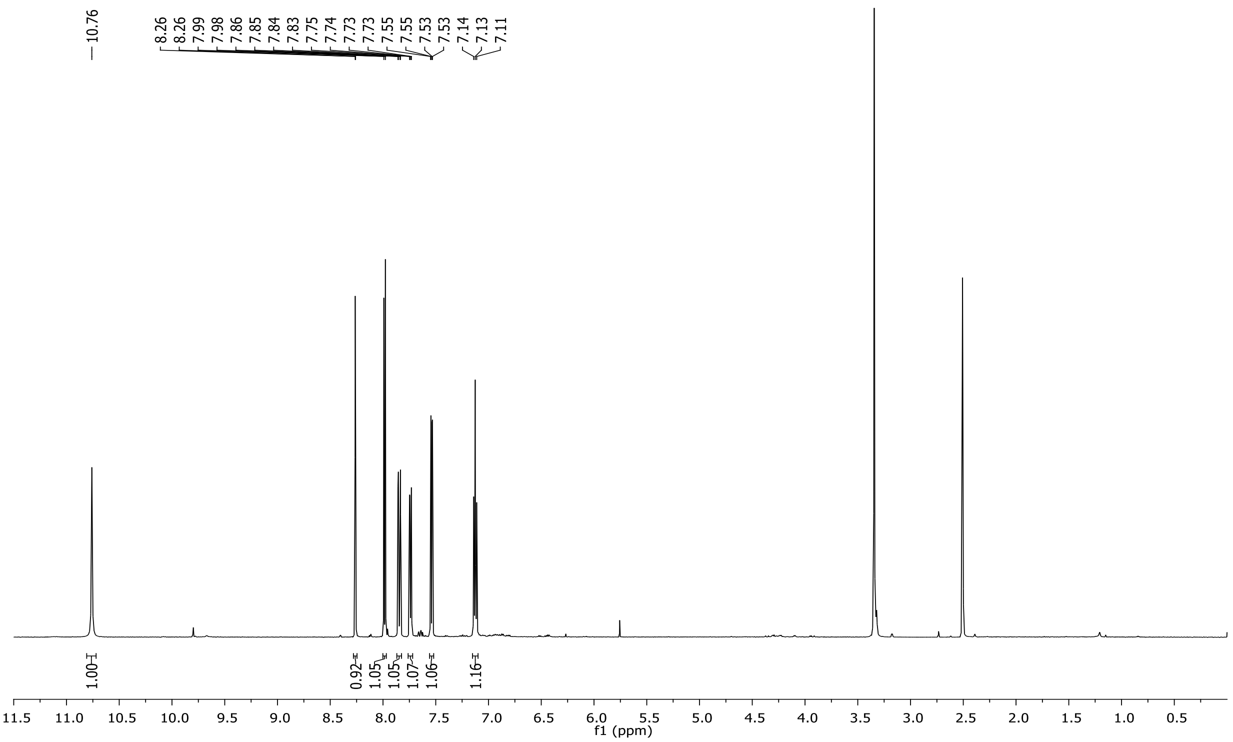

b)

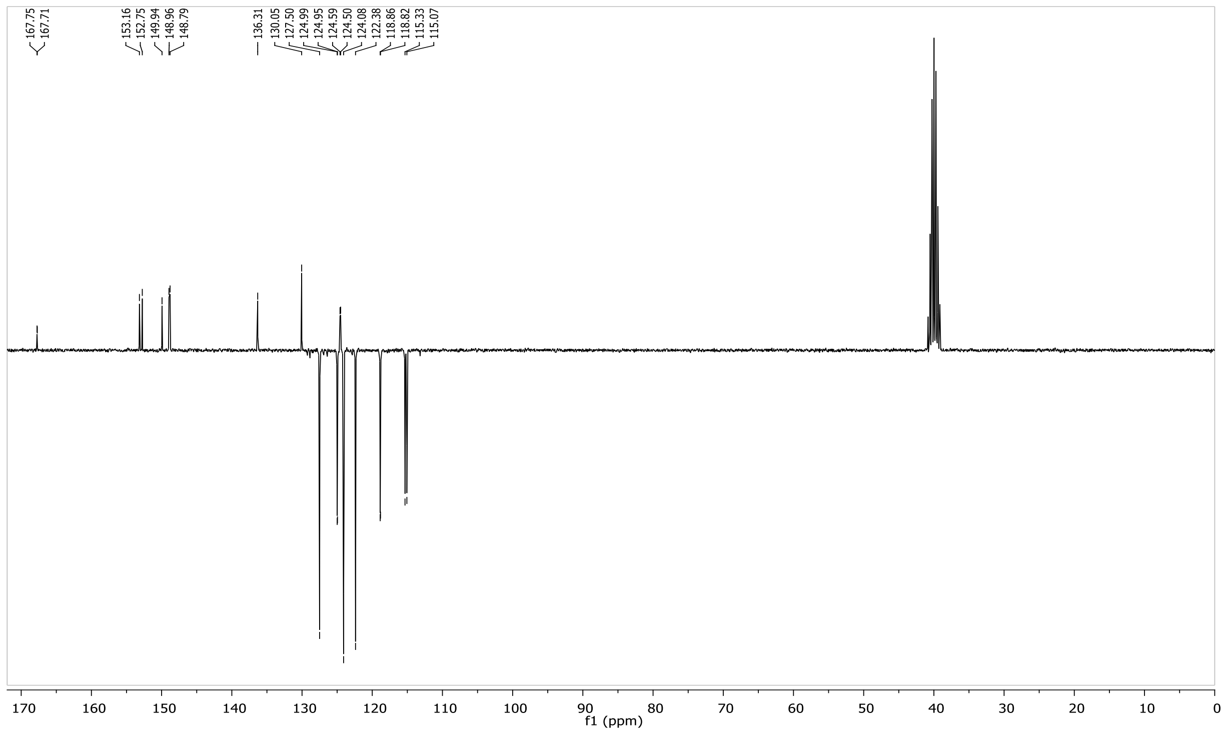

Fig. S3 a) ^{1}H NMR and b) ^{13}C NMR of compd. **9c**

a)

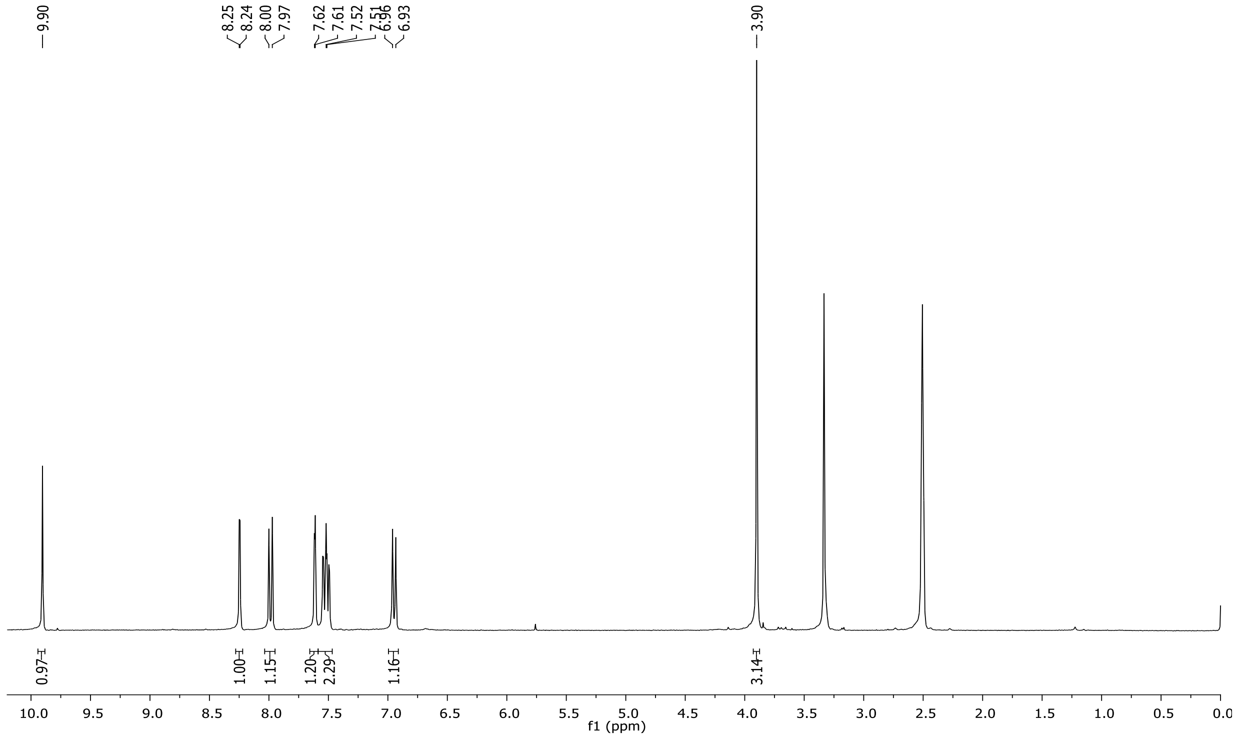

b)

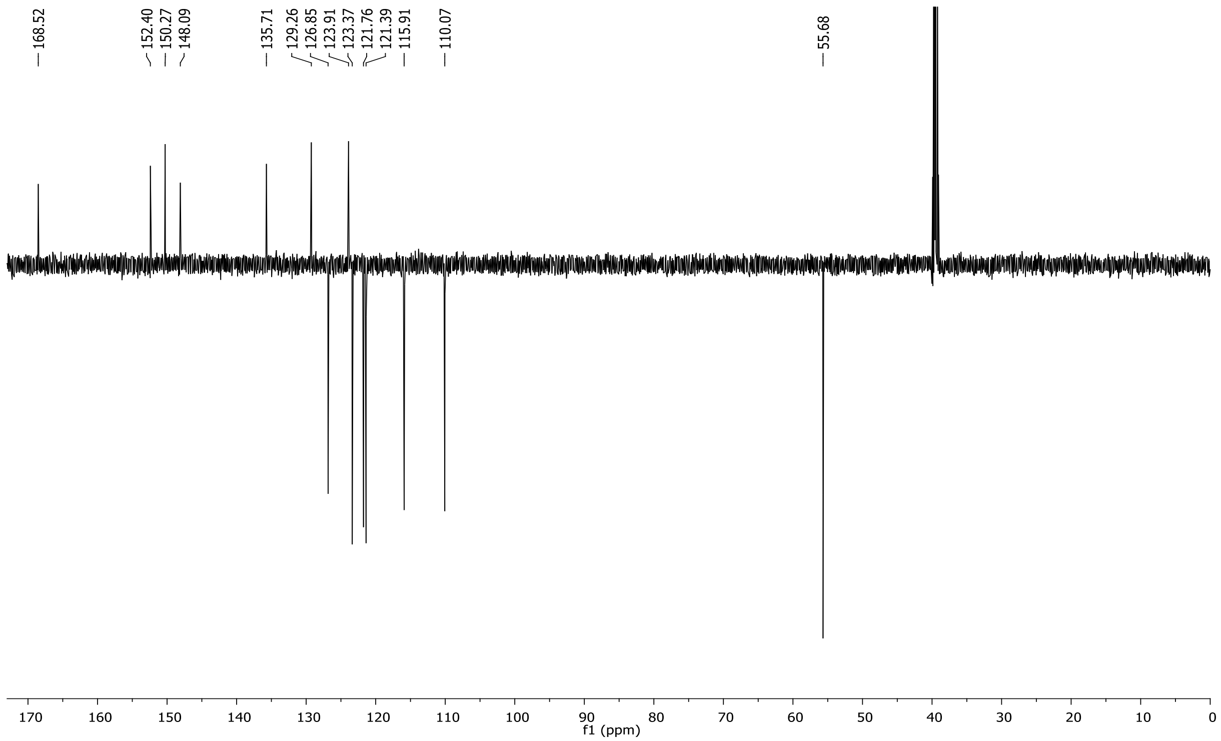

Fig. S4 a) ^{1}H NMR and b) ^{13}C NMR of compd. **10a**

a)

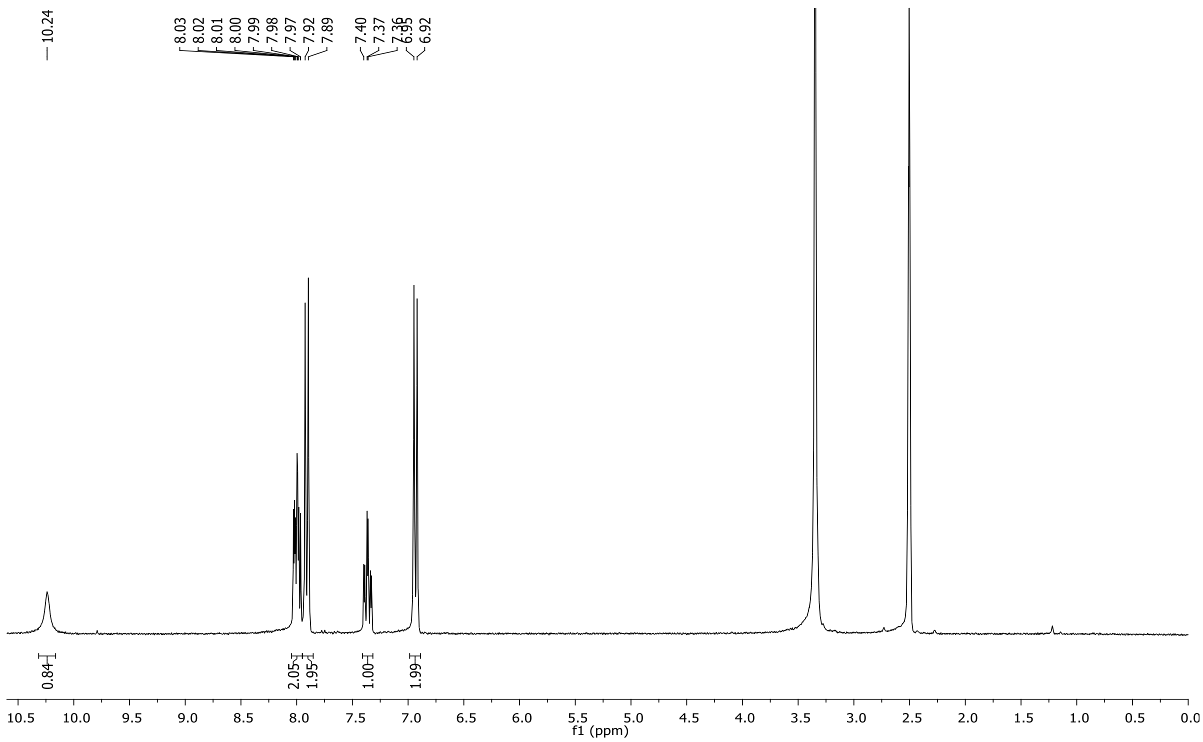

b)

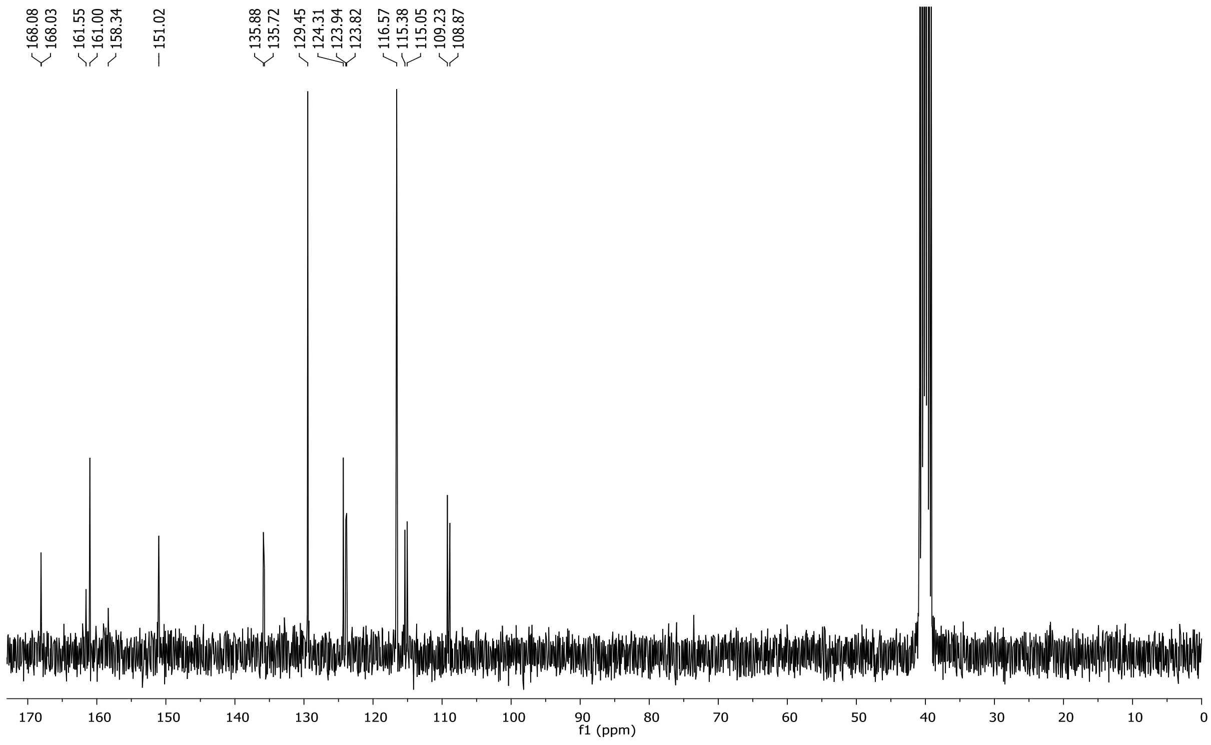

Fig. S5 a) ^{1}H NMR and b) ^{13}C NMR of compd. **10b**

a)

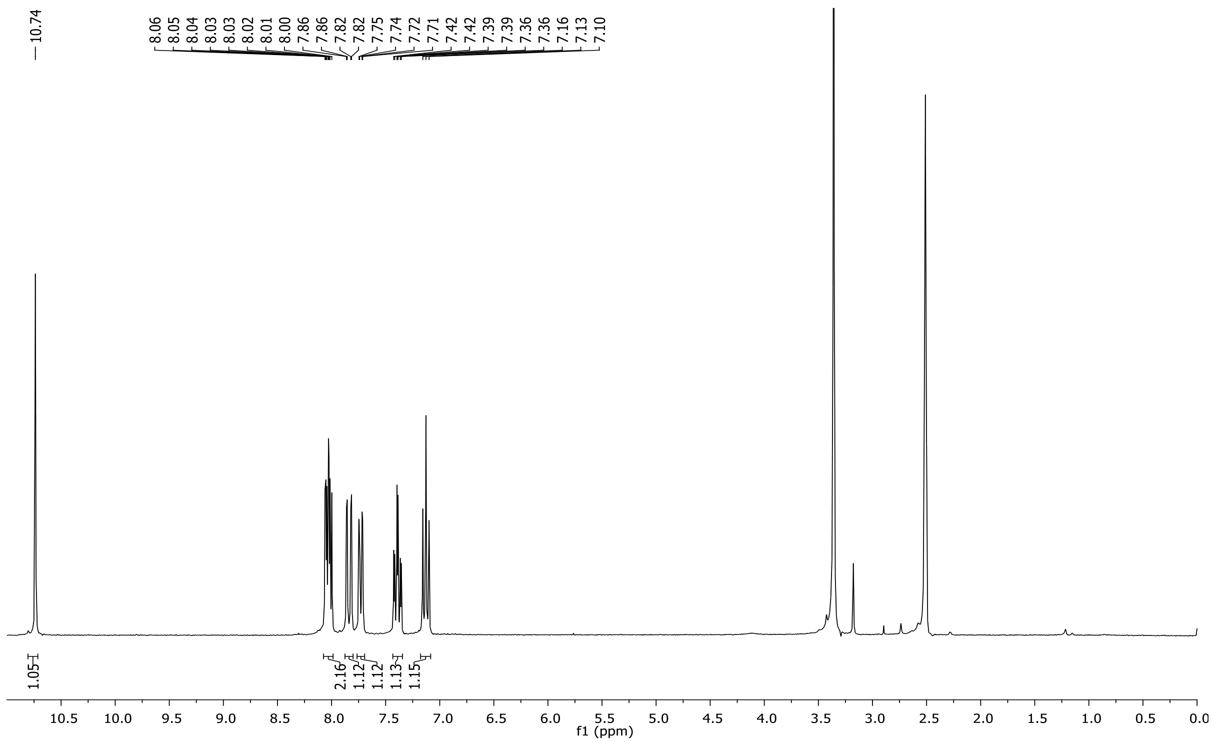

b)

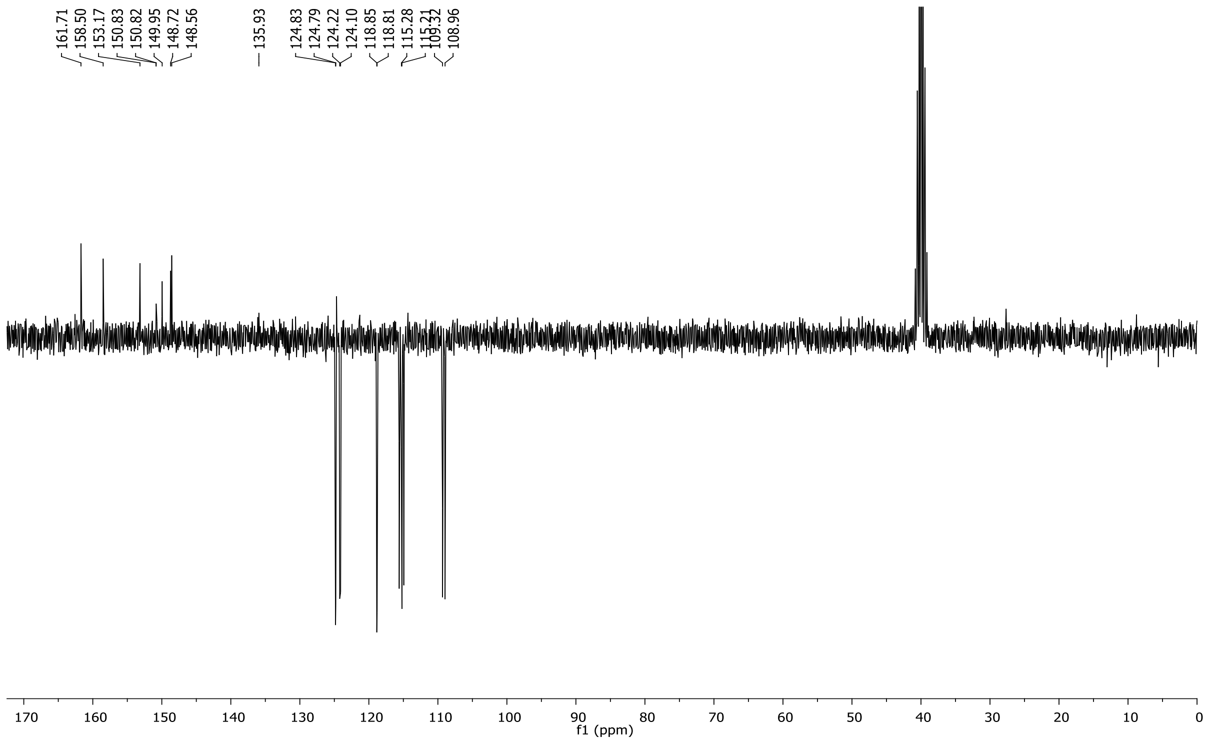

Fig. S6 a) ^{1}H NMR and b) ^{13}C NMR of compd. **10c**

a)

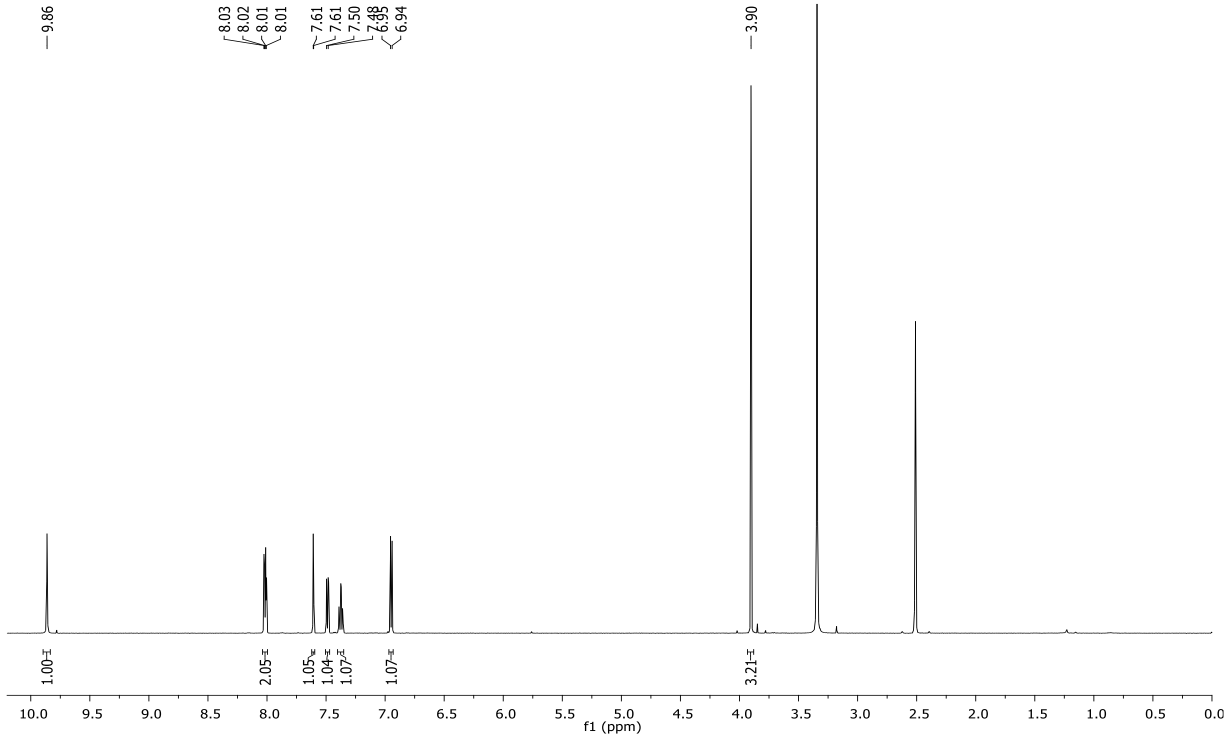

b)

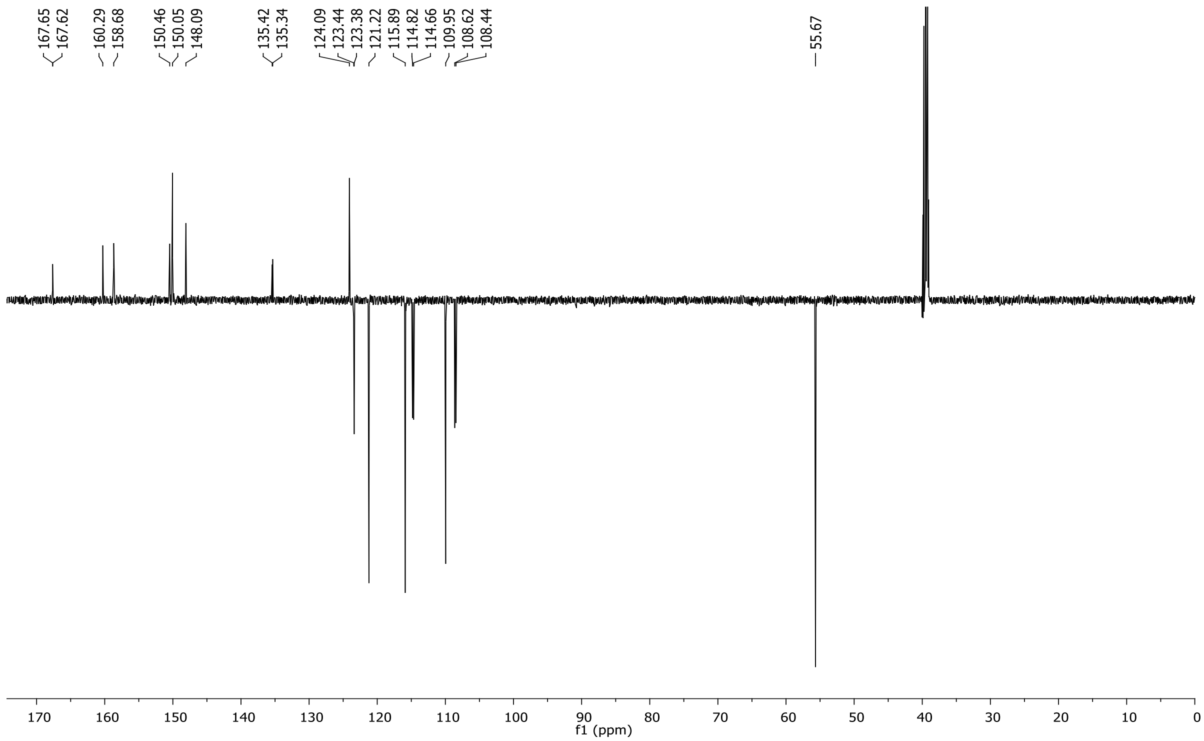

Fig. S7 a) ^{1}H NMR and b) ^{13}C NMR of compd. **11a**

a)

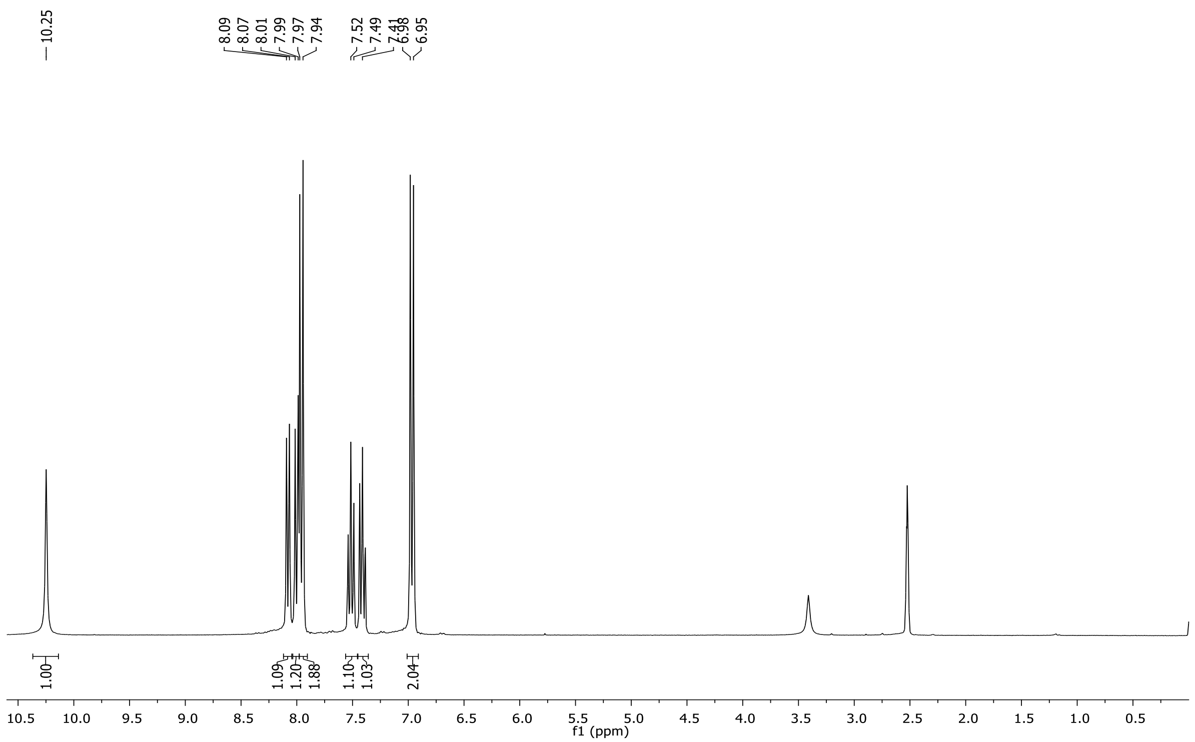

b)

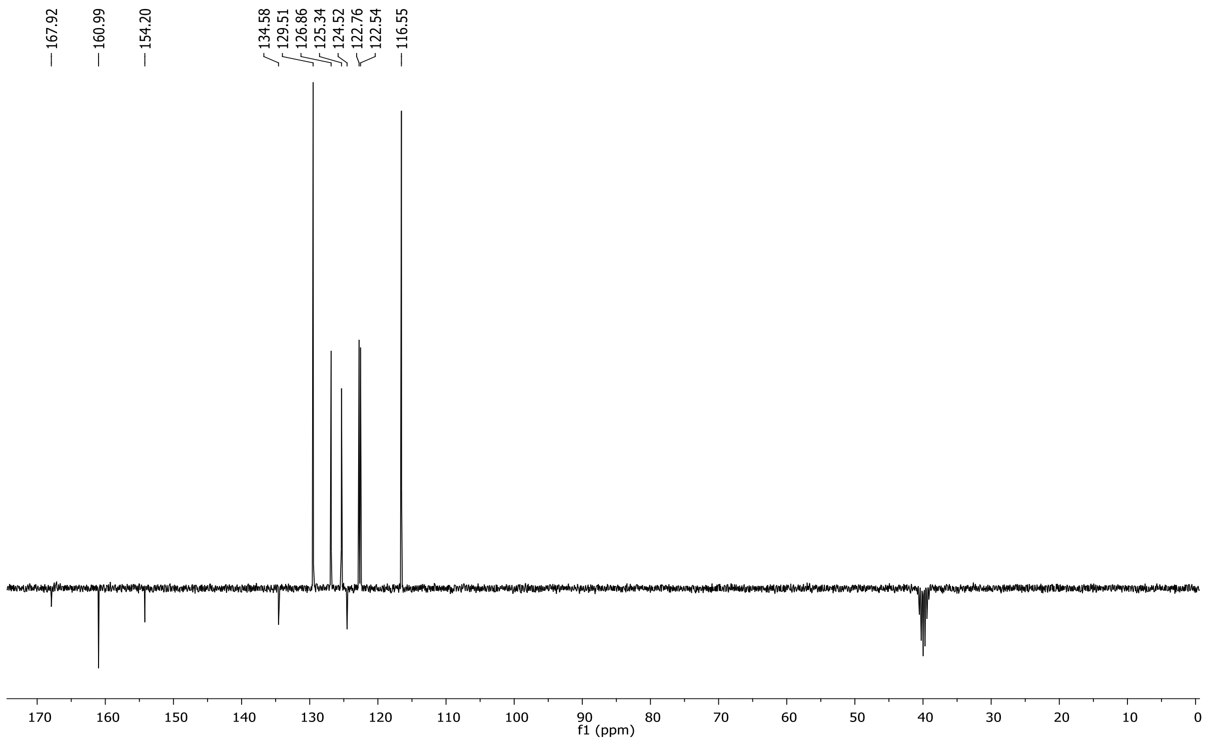

Fig. S8 a) ^{1}H NMR and b) ^{13}C NMR of compd. **11b**

a)

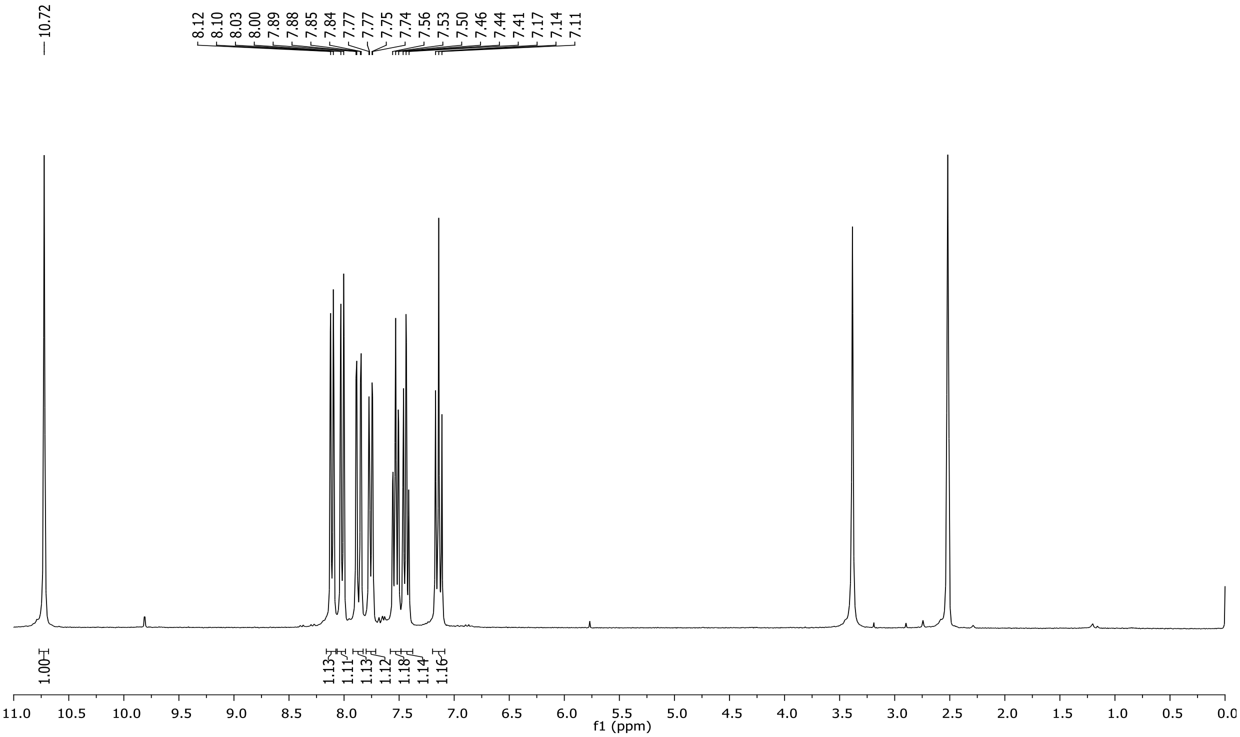

b)

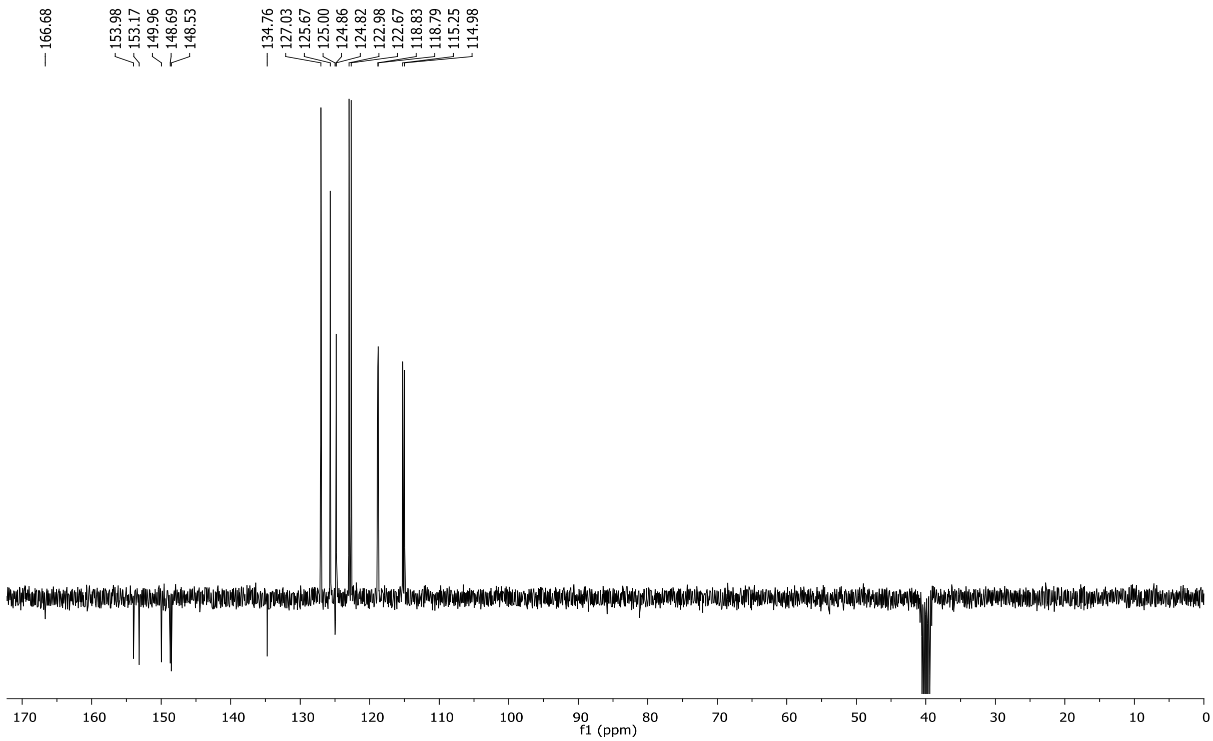

Fig. S9 a) ^{1}H NMR and b) ^{13}C NMR of compd. **11c**

a)

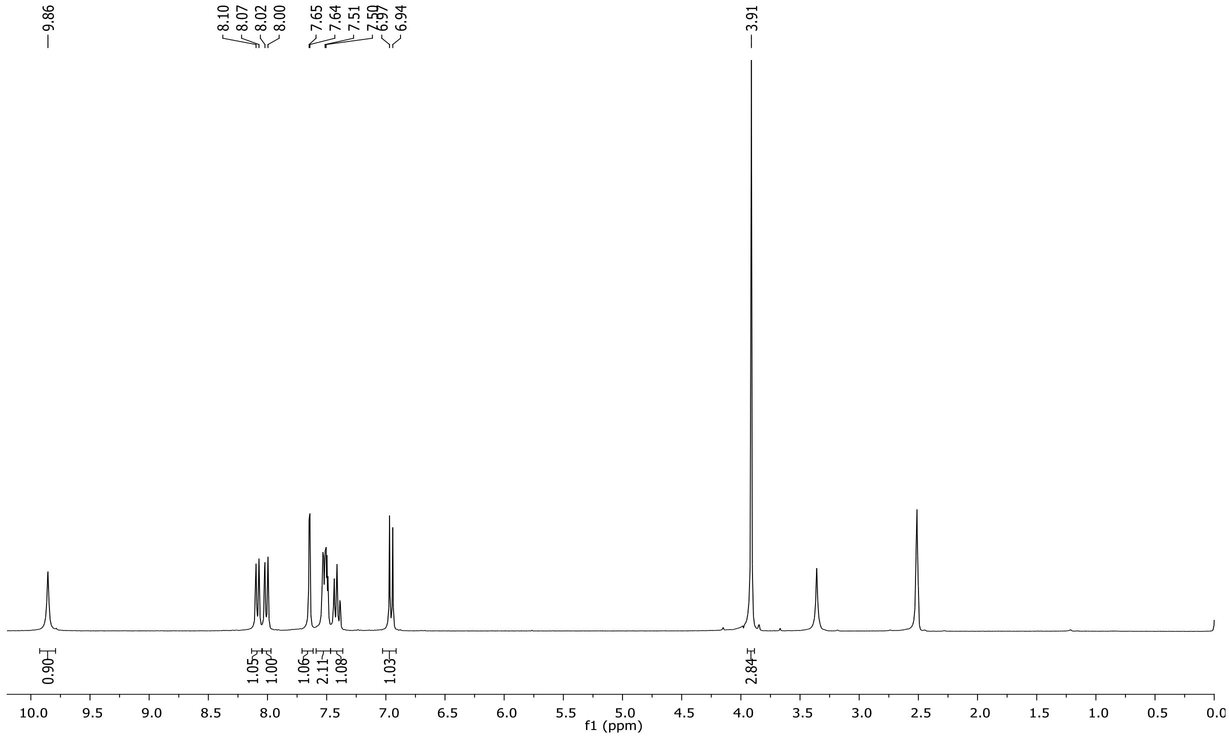

b)

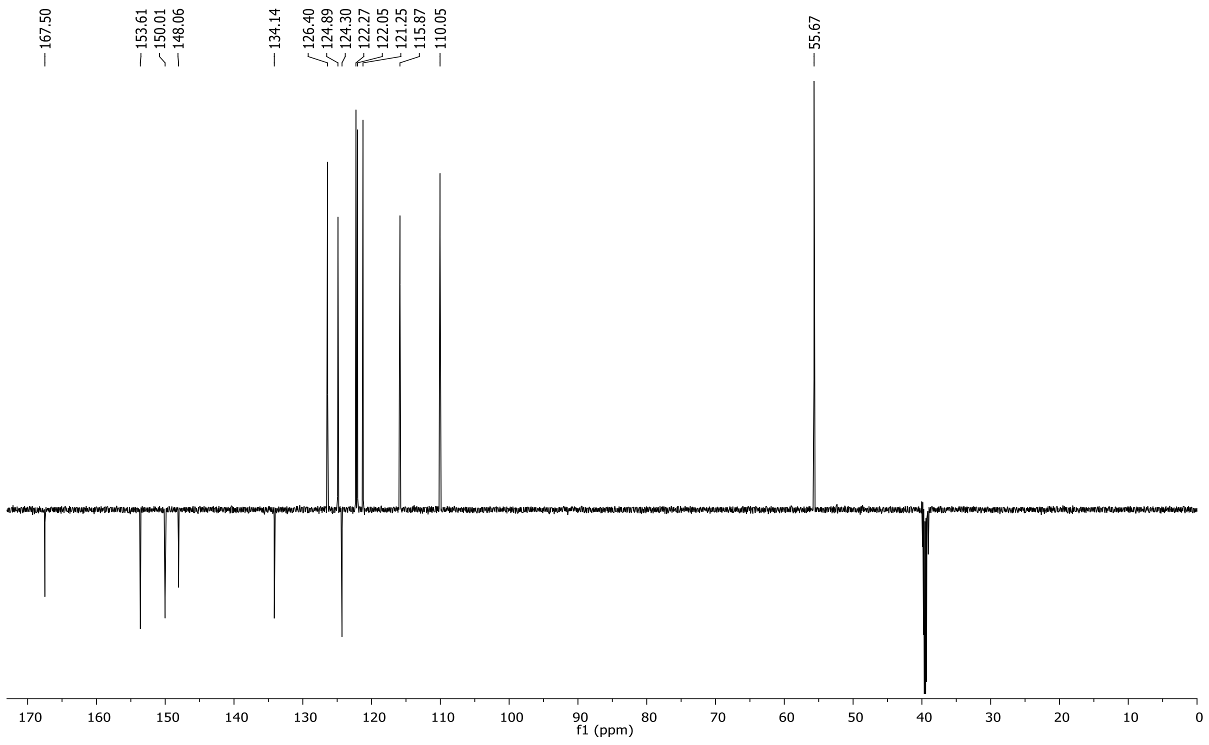

Fig. S10 a) ^{1}H NMR and b) ^{13}C NMR of compd. **12a**

a)

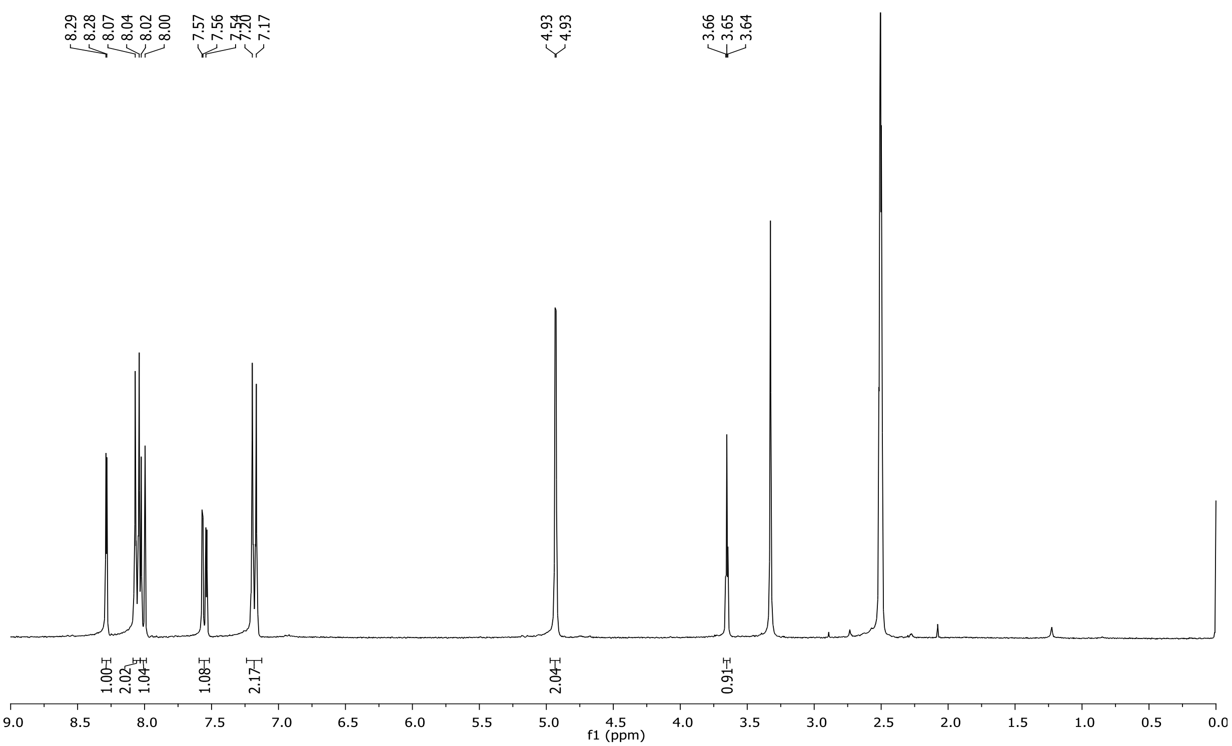

b)

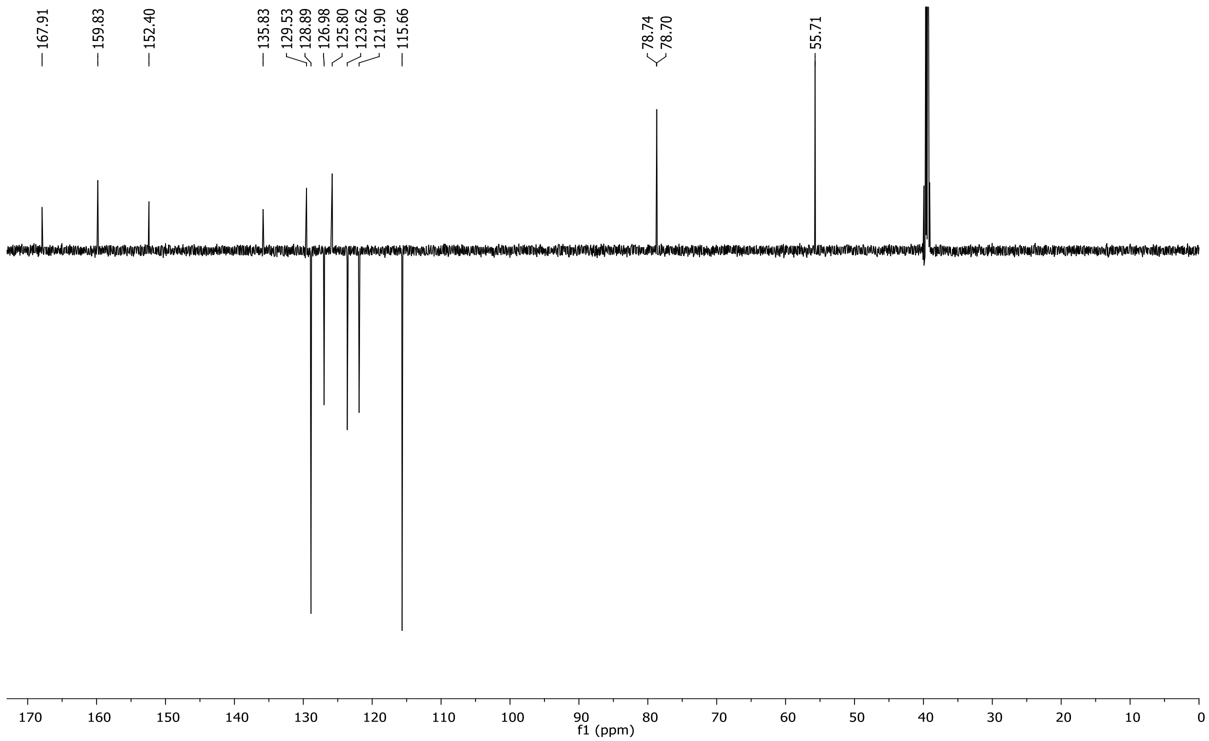

Fig. S11 a) ^{1}H NMR and b) ^{13}C NMR of compd. **12b**

a)

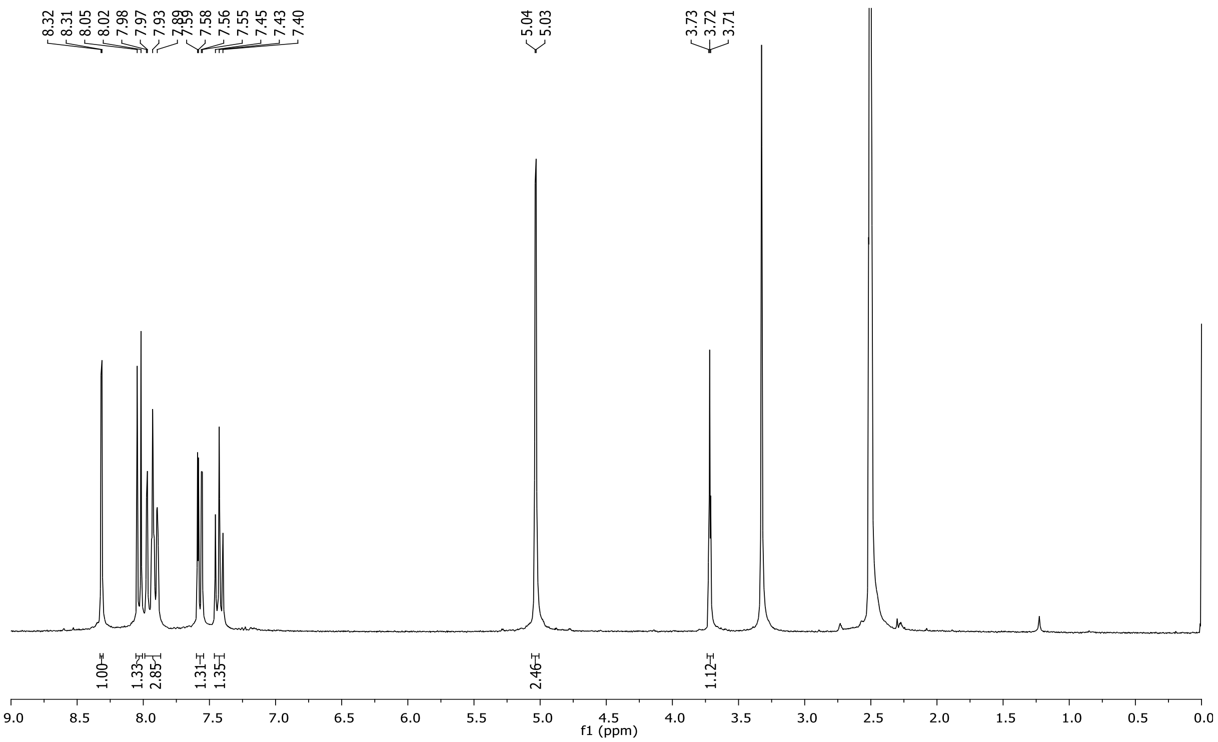

b)

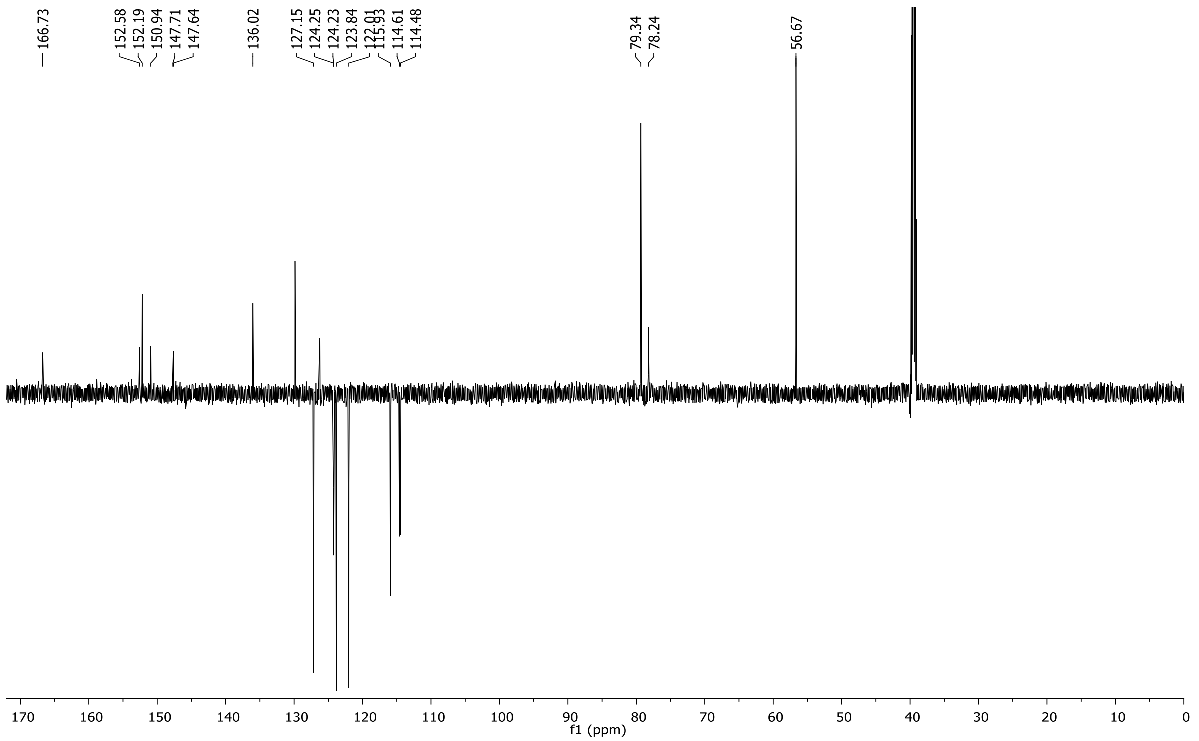

Fig. S12 a) ^{1}H NMR and b) ^{13}C NMR of compd. **12c**

a)

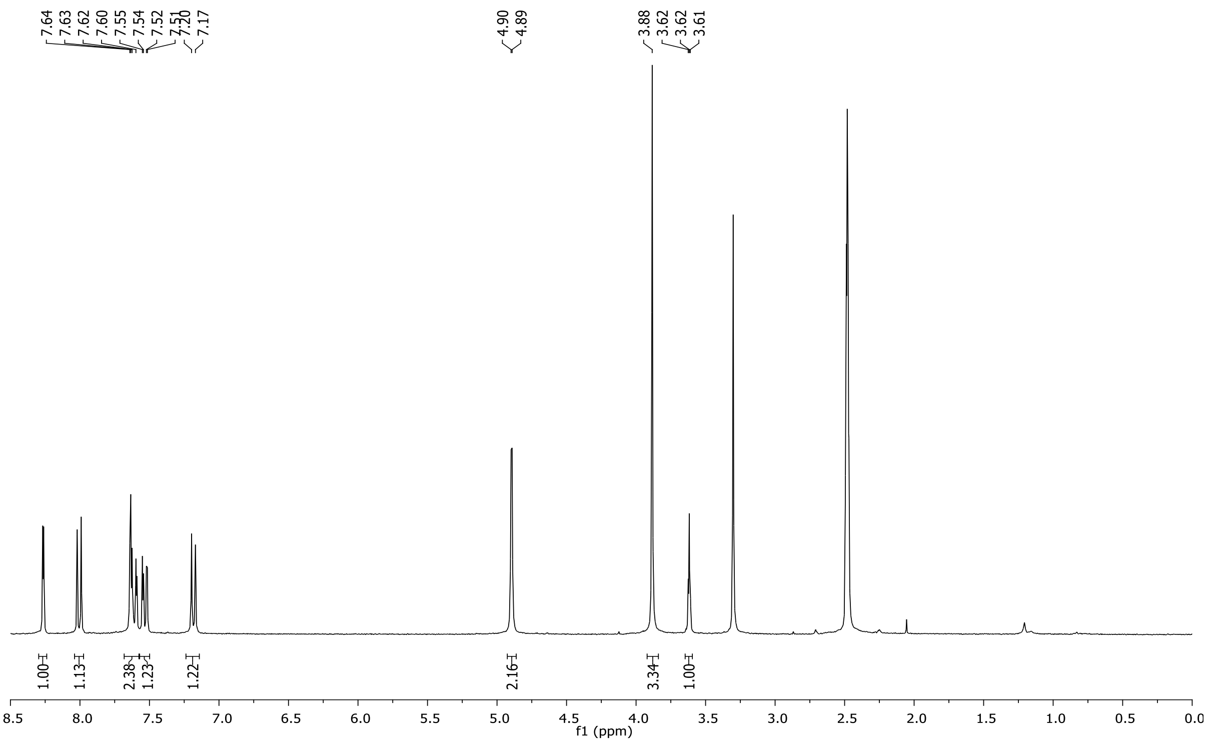

b)

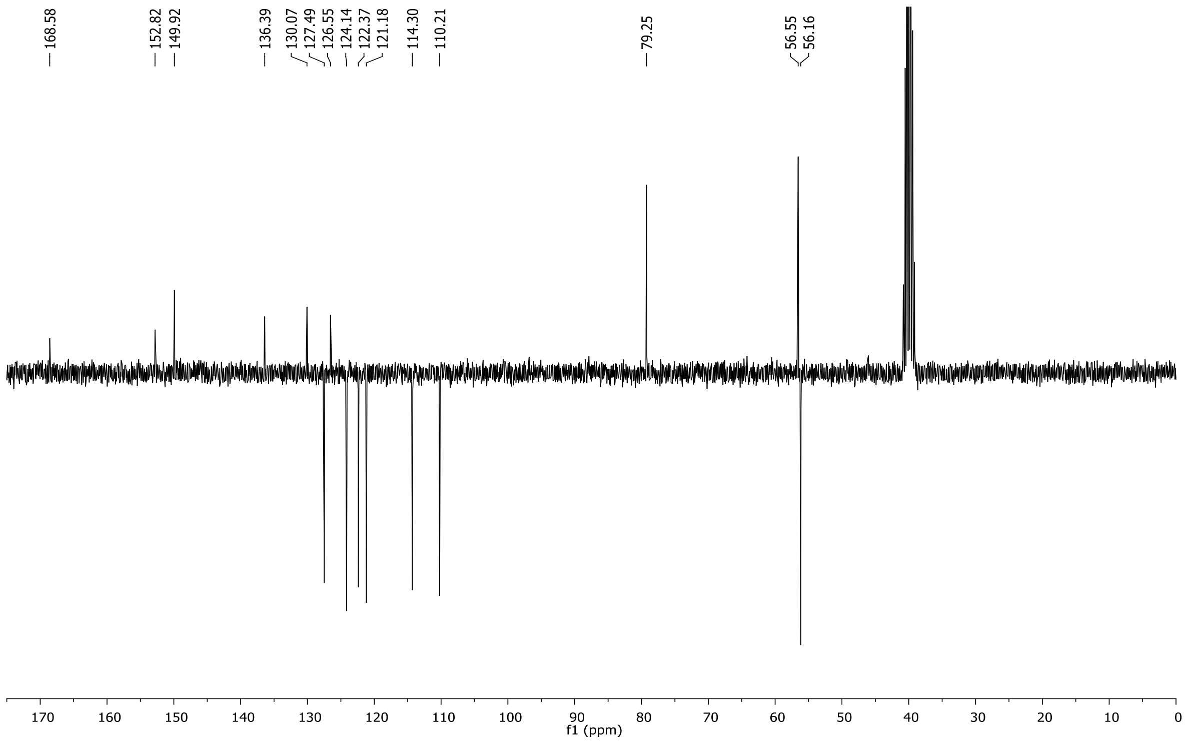

Fig. S13 a) ^{1}H NMR and b) ^{13}C NMR of compd. **13a**

a)

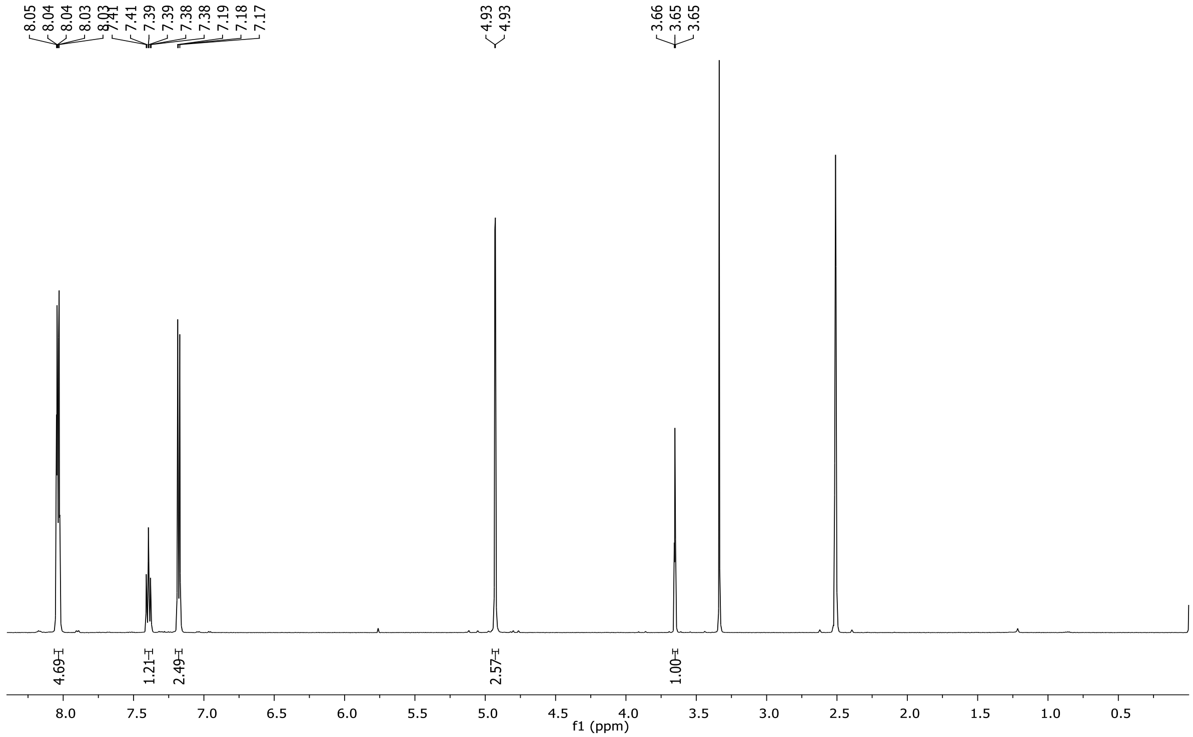

b)

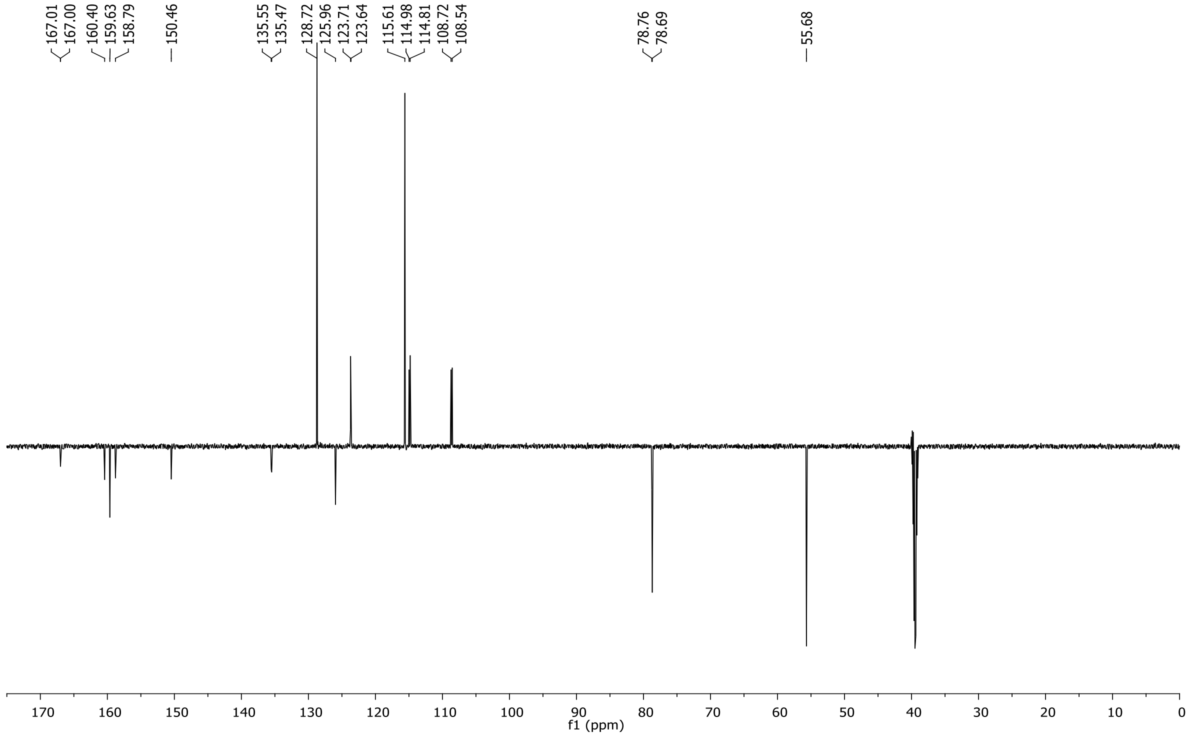

Fig. S14 a) ^{1}H NMR and b) ^{13}C NMR of compd. **13b**

a)

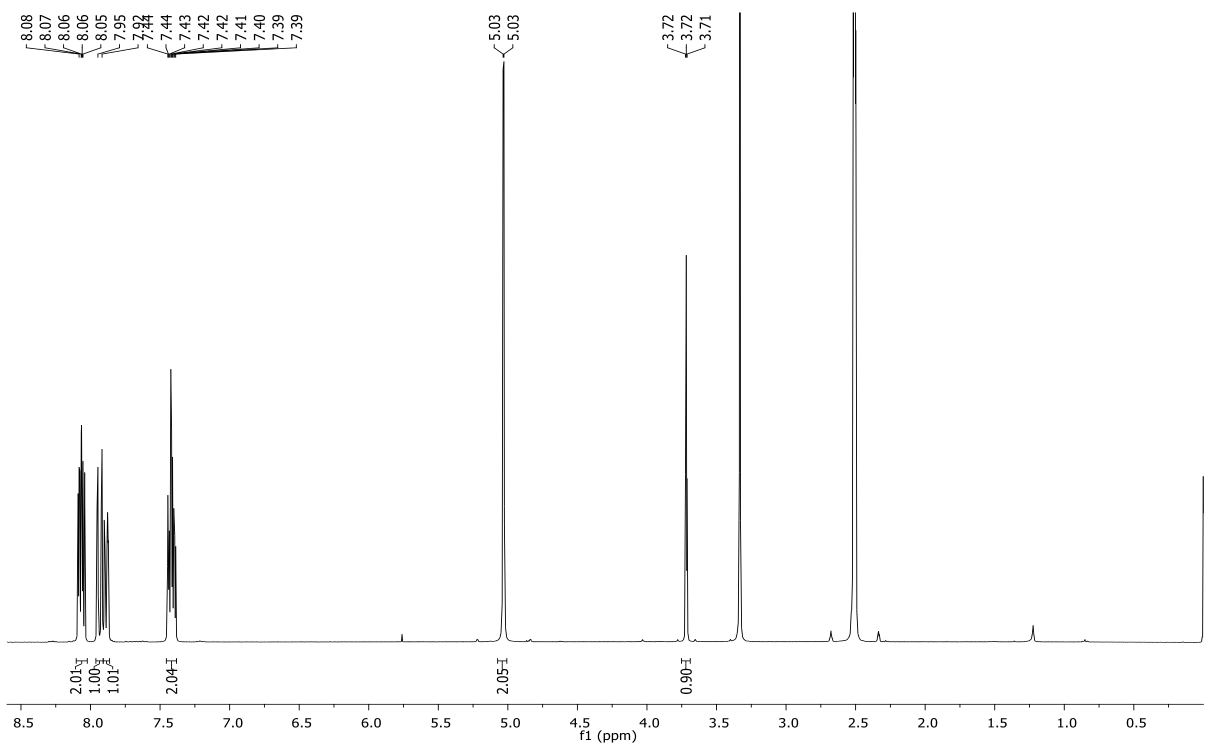

b)

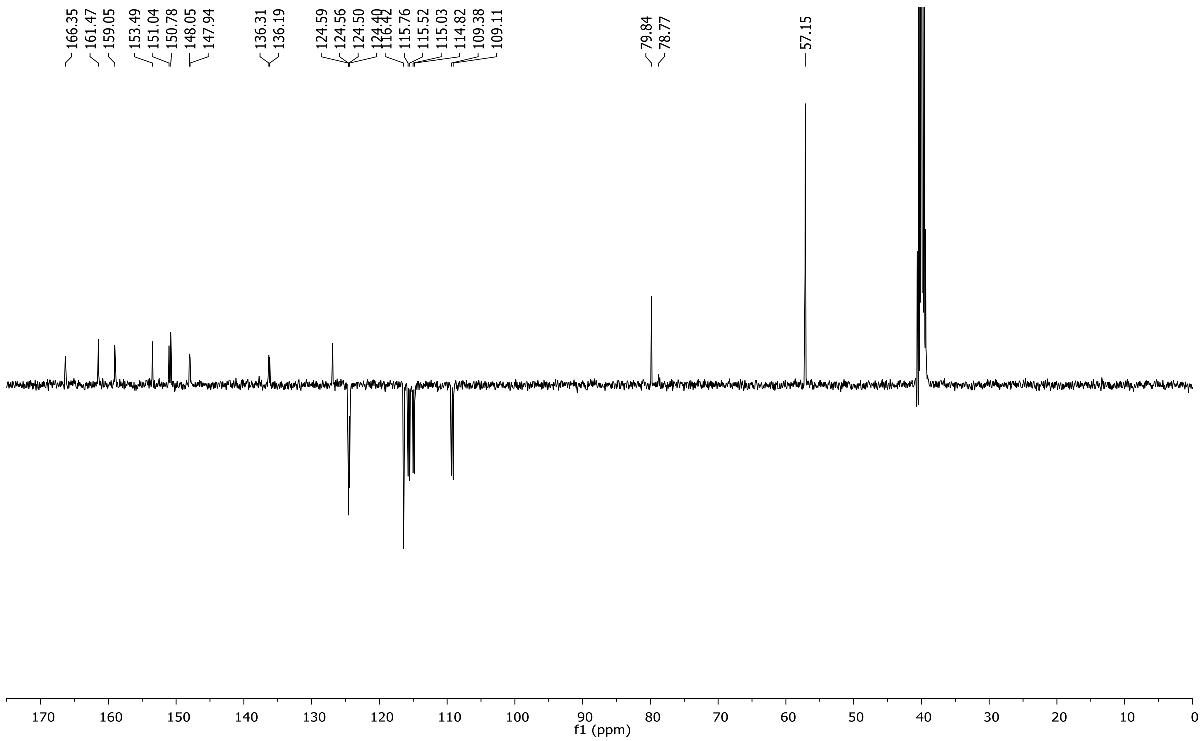

Fig. S15 a) ^{1}H NMR and b) ^{13}C NMR of compd. **13c**

a)

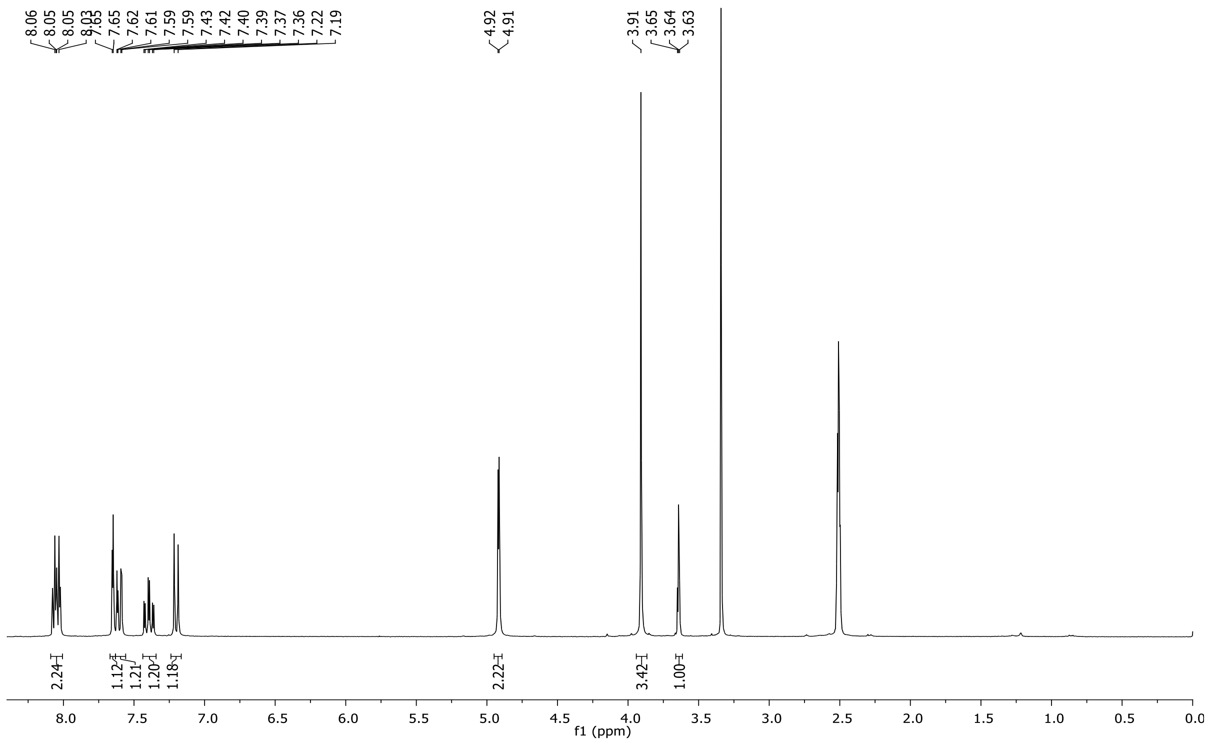

b)

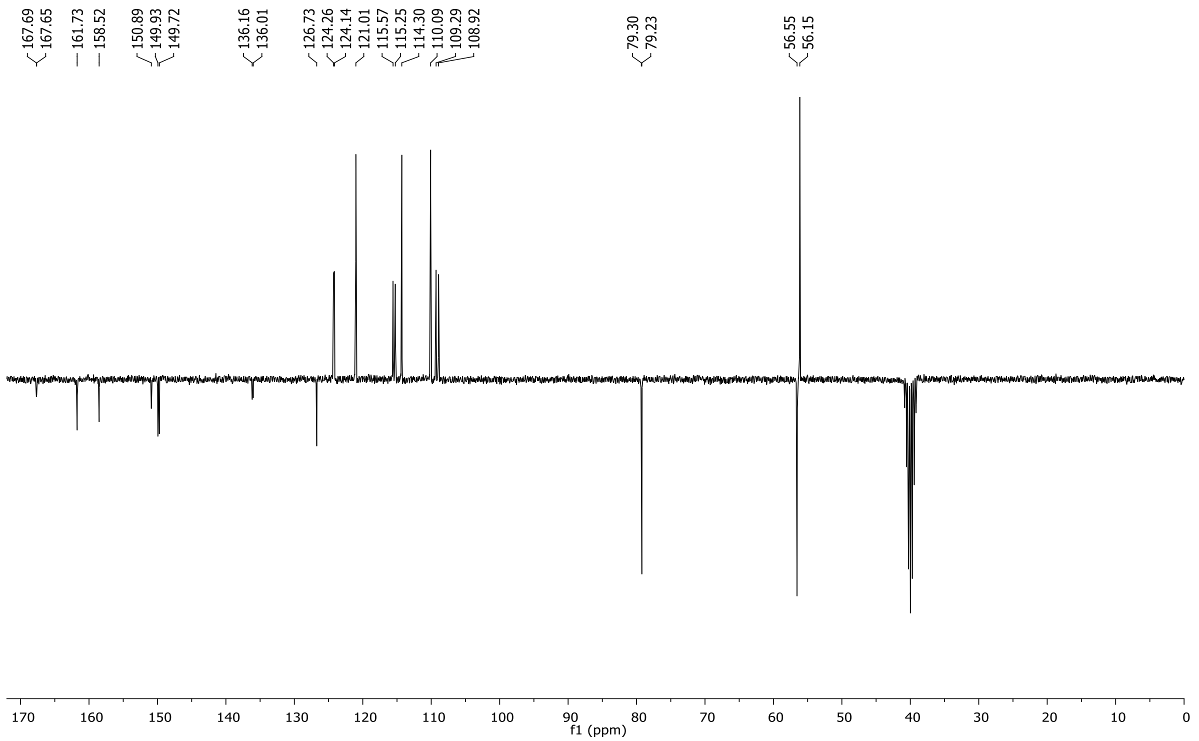

Fig. S16 a) ^{1}H NMR and b) ^{13}C NMR of compd. **14a**

a)

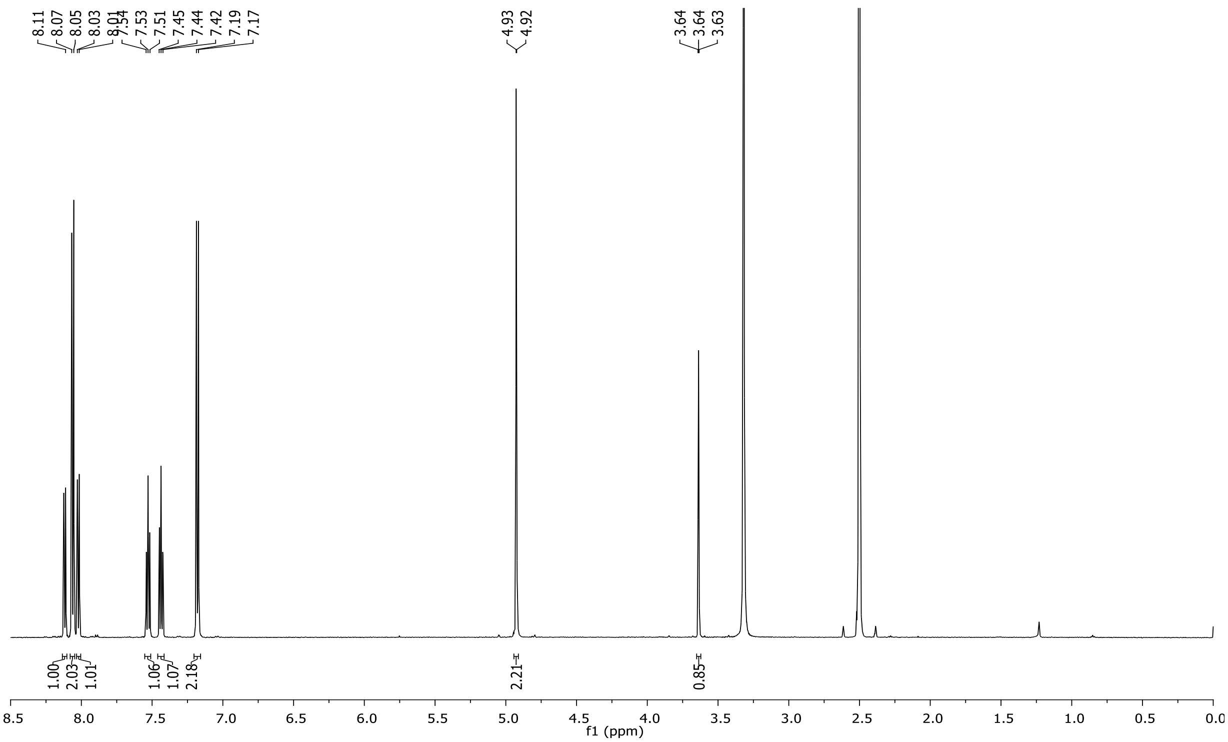

b)

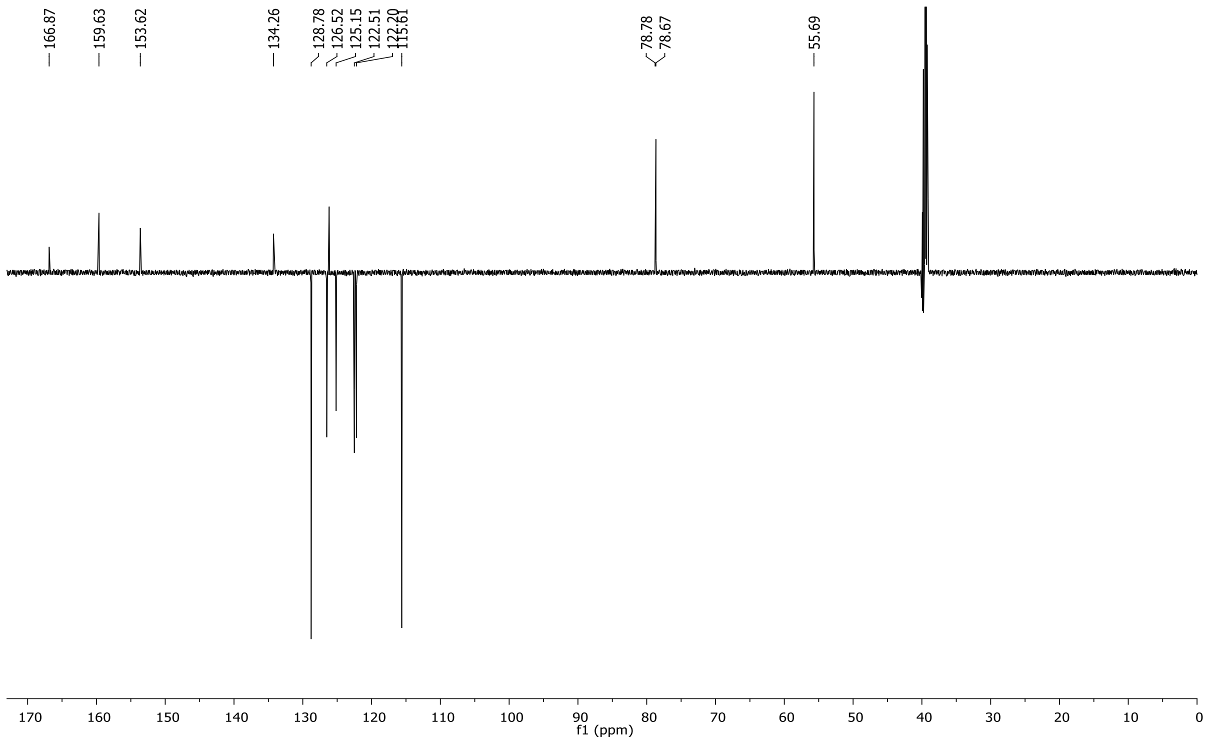

Fig. S17 a) ^{1}H NMR and b) ^{13}C NMR of compd. **14b**

a)

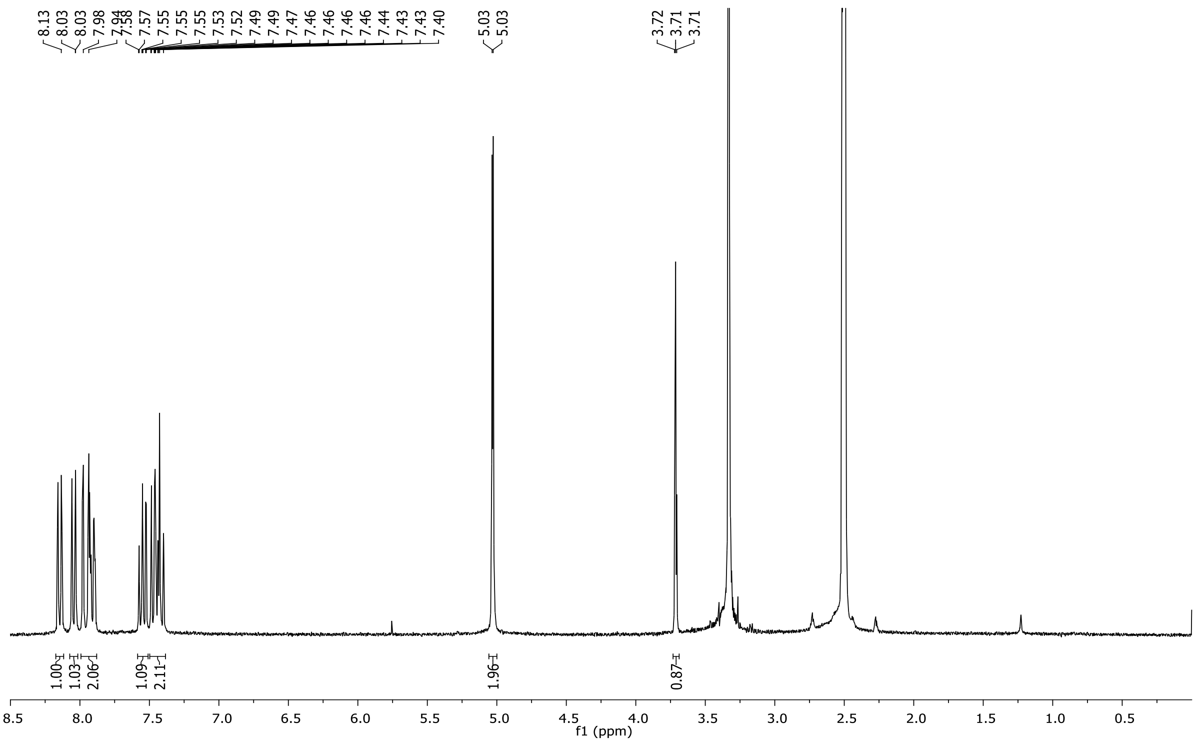

b)

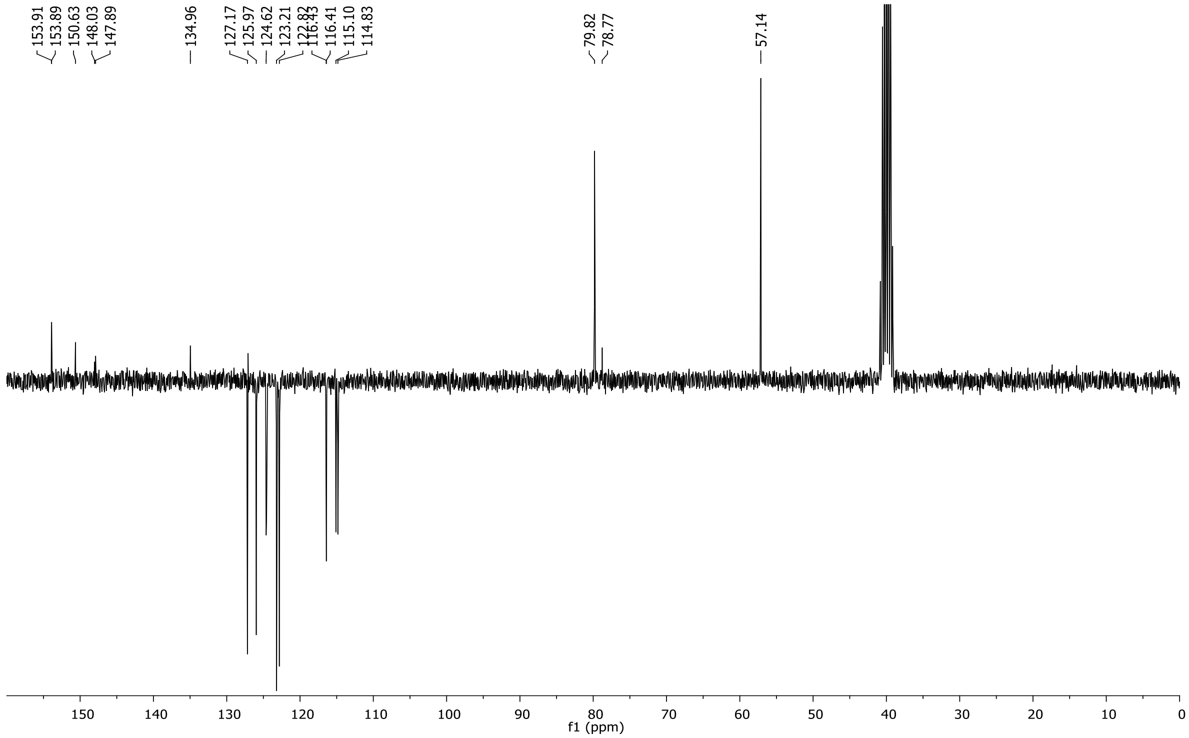

Fig. S18 a) ^{1}H NMR and b) ^{13}C NMR of compd. **14c**

a)

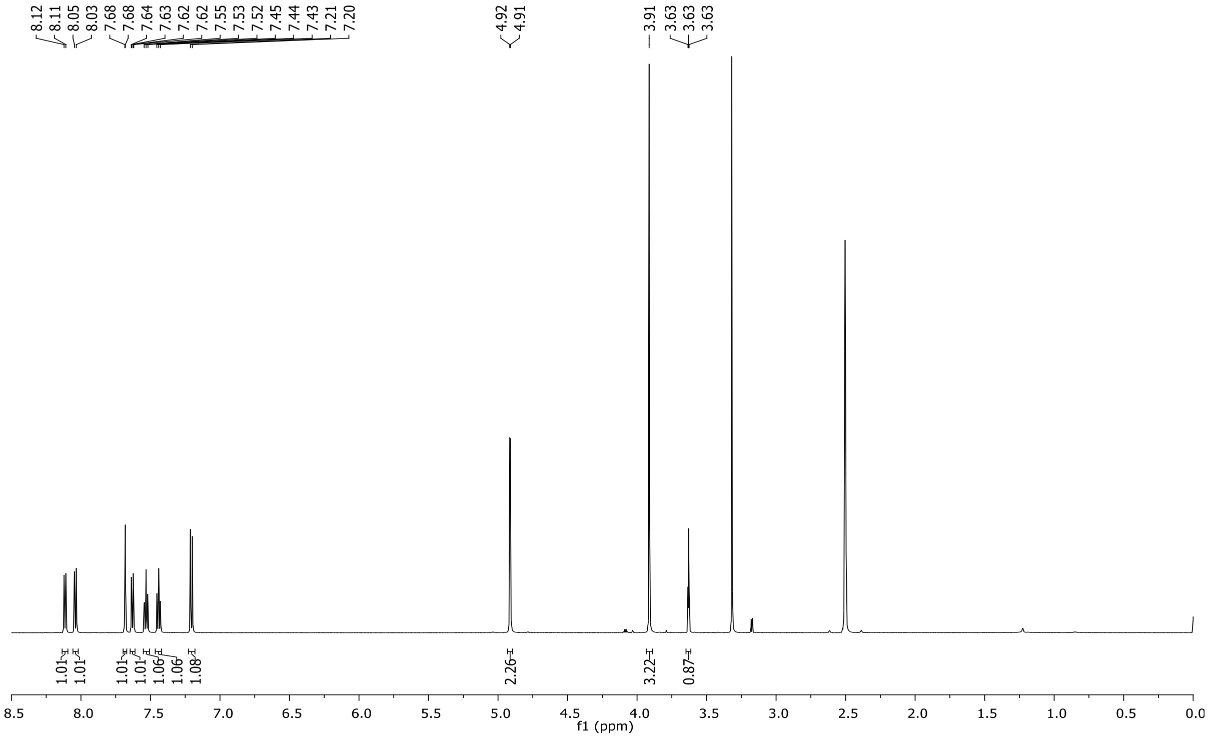

b)

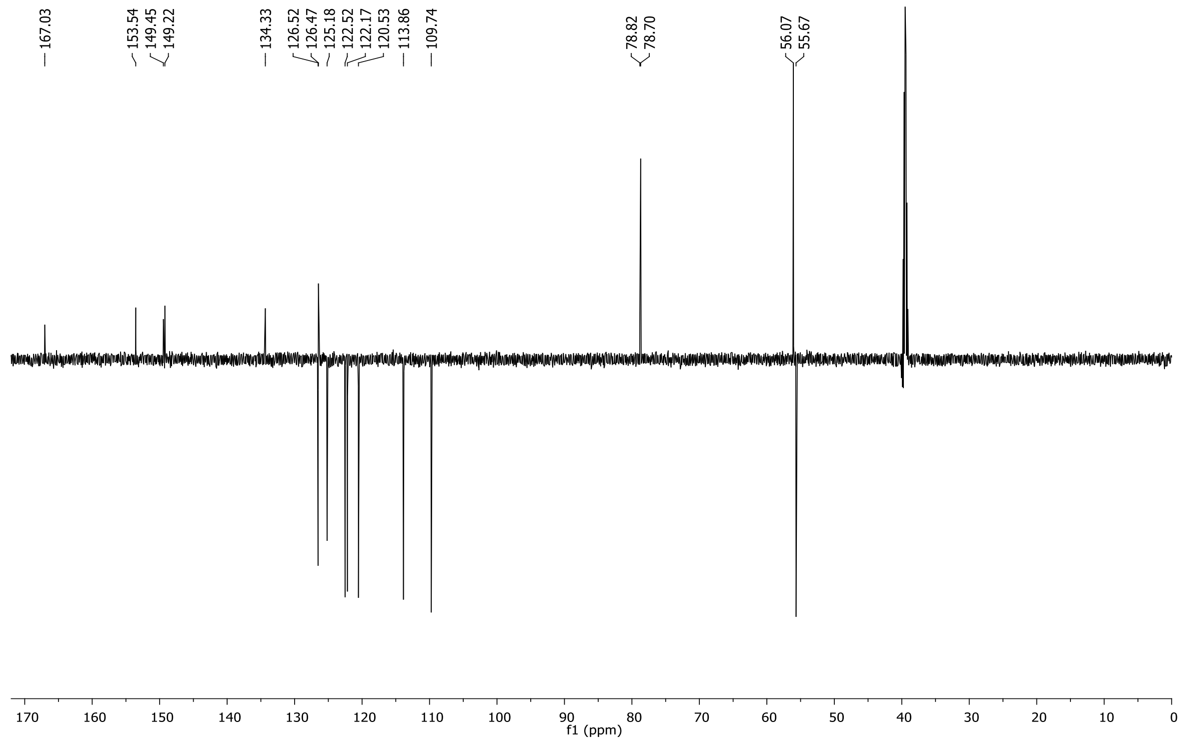

Fig. S19 a) ^{1}H NMR and b) ^{13}C NMR of compd. **15a**

a)

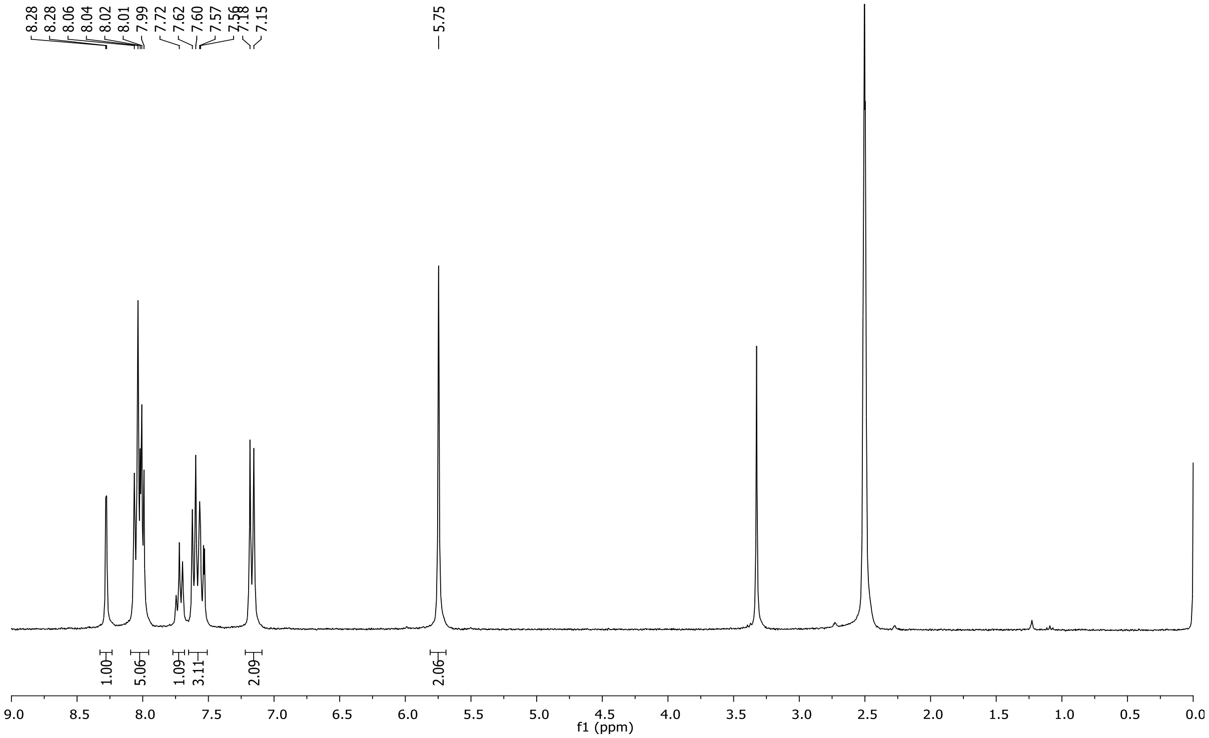

b)

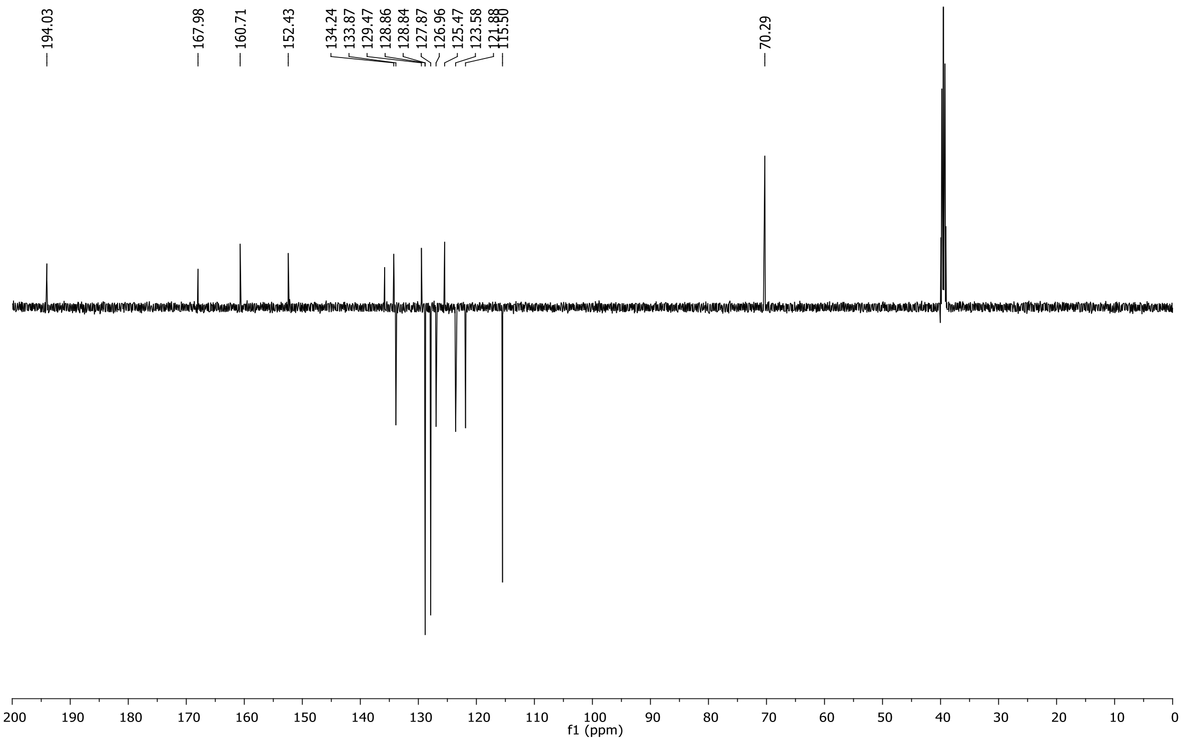

Fig. S20 a) ^{1}H NMR and b) ^{13}C NMR of compd. **15b**

a)

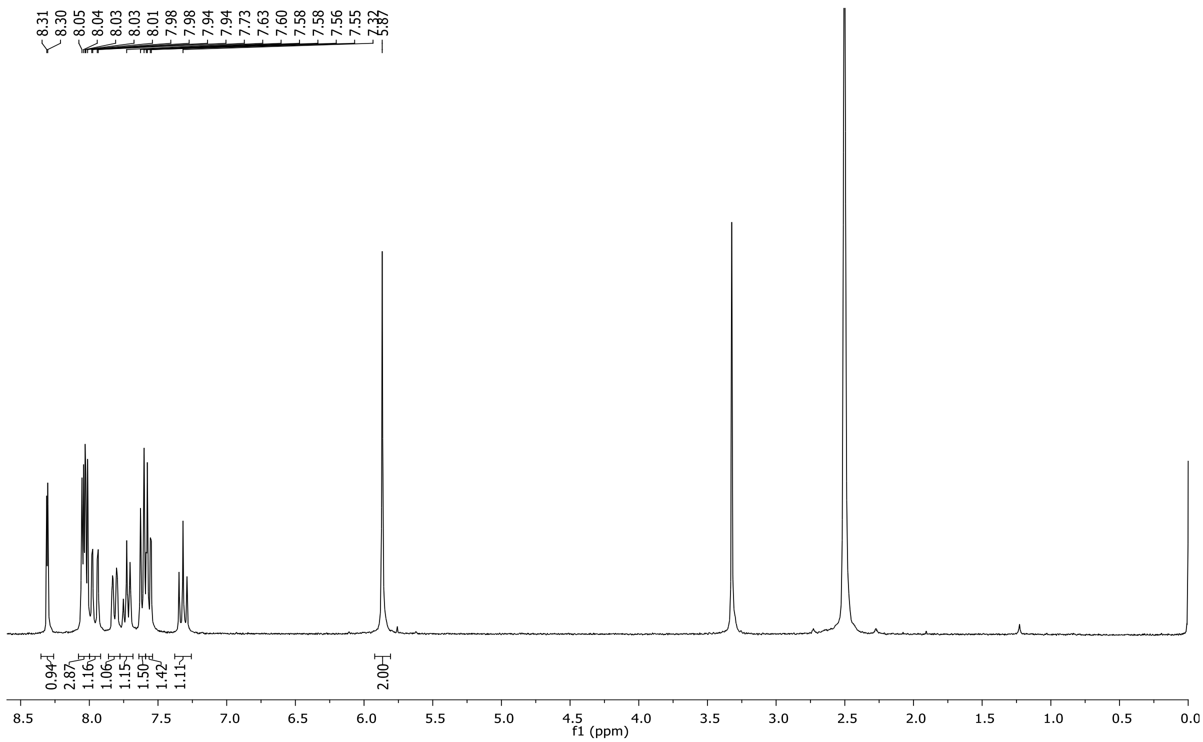

b)

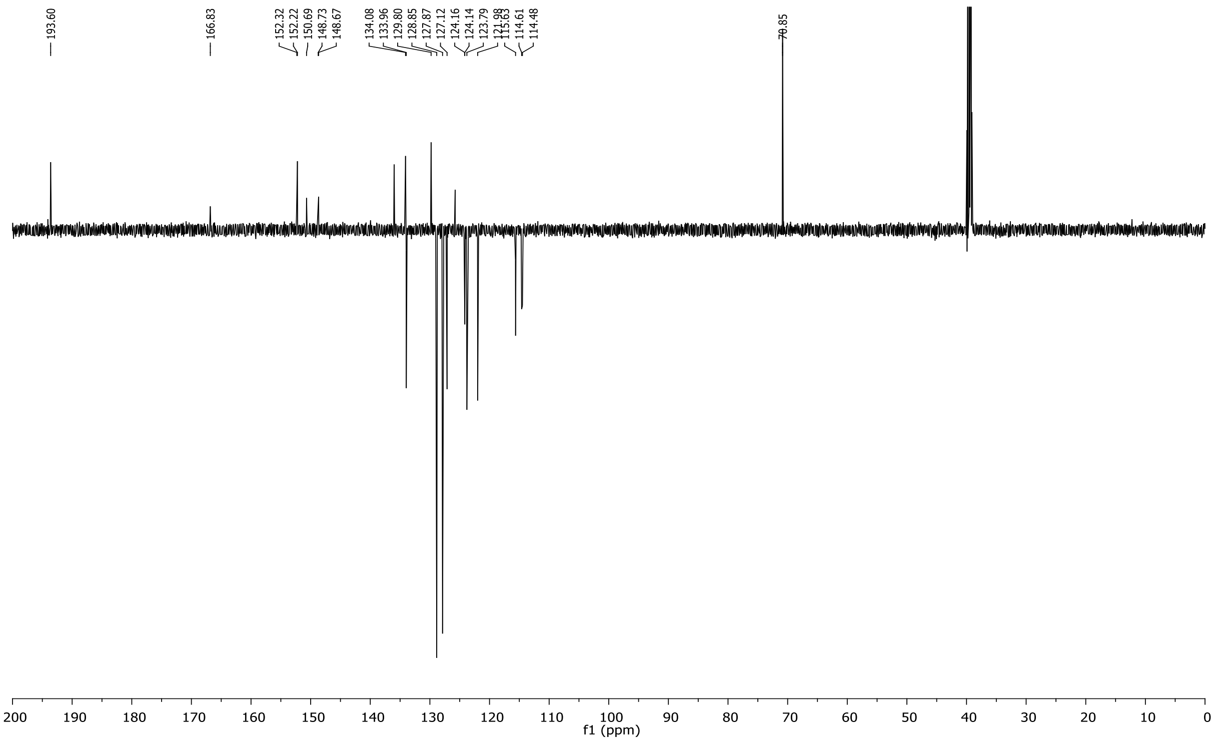

Fig. S21 a) ^{1}H NMR and b) ^{13}C NMR of compd. **15c**

a)

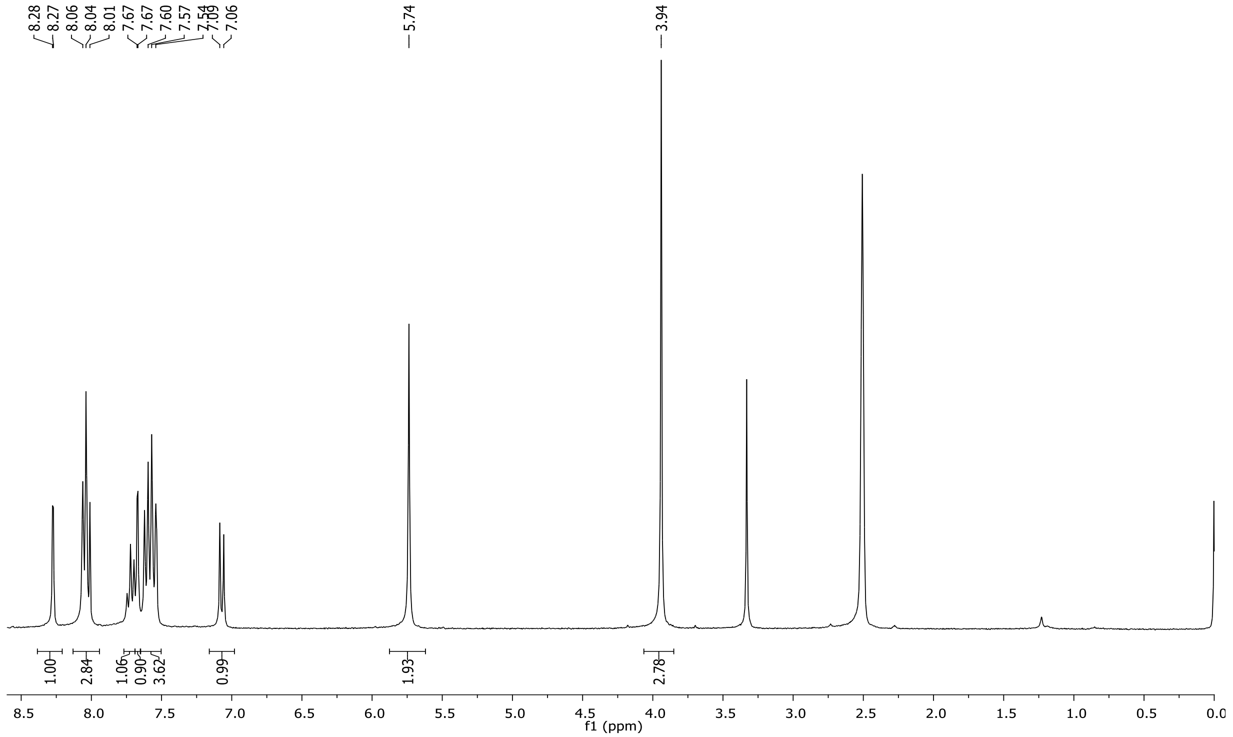

b)

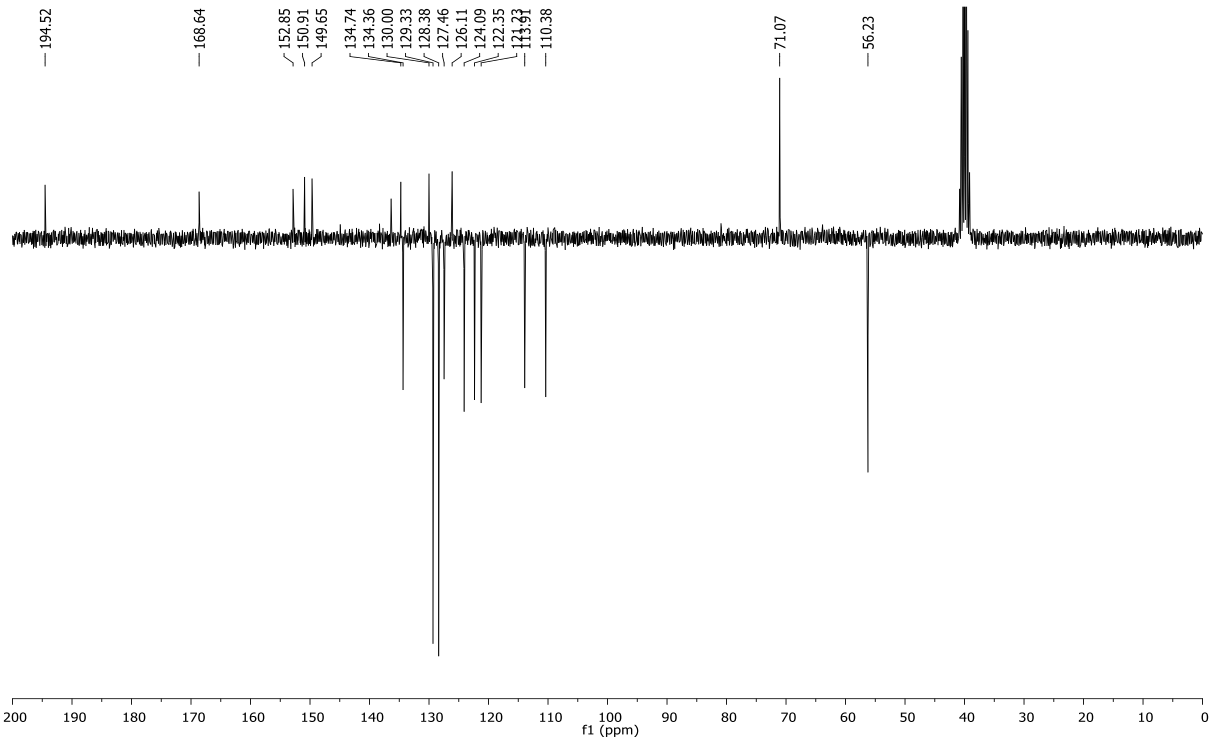

Fig. S22 a) ^{1}H NMR and b) ^{13}C NMR of compd. **16a**

a)

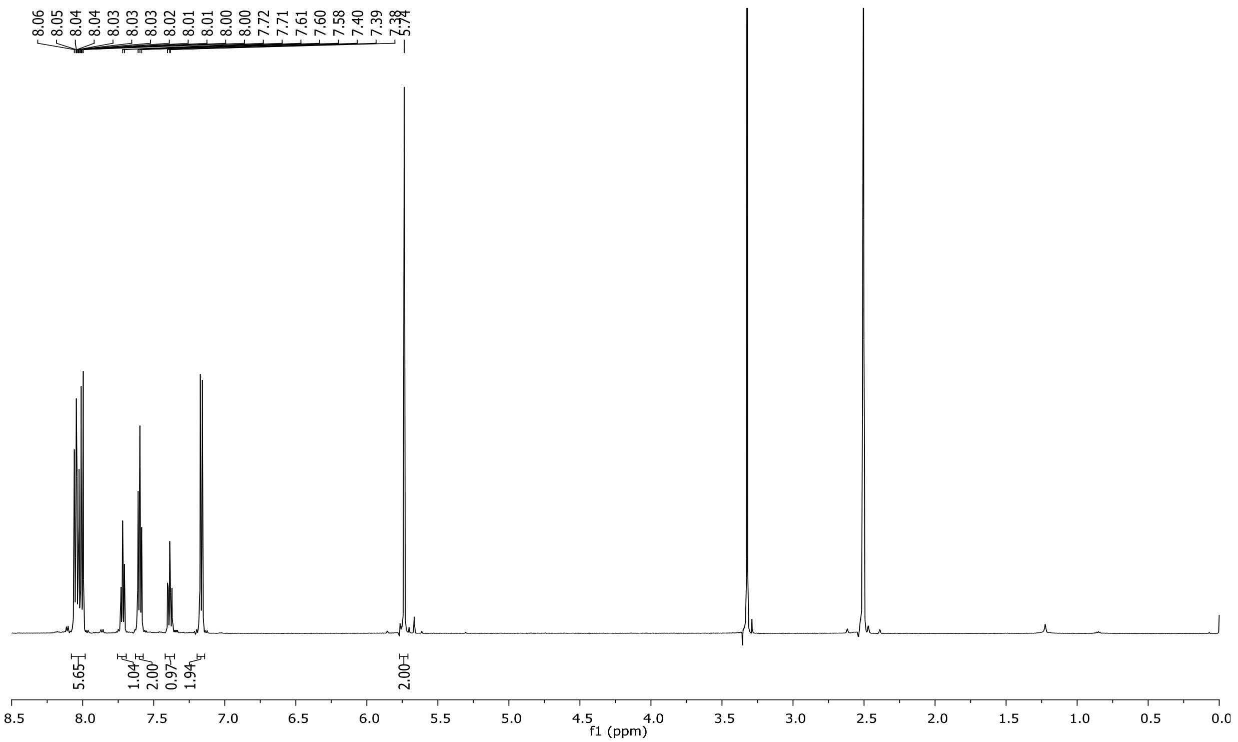

b)

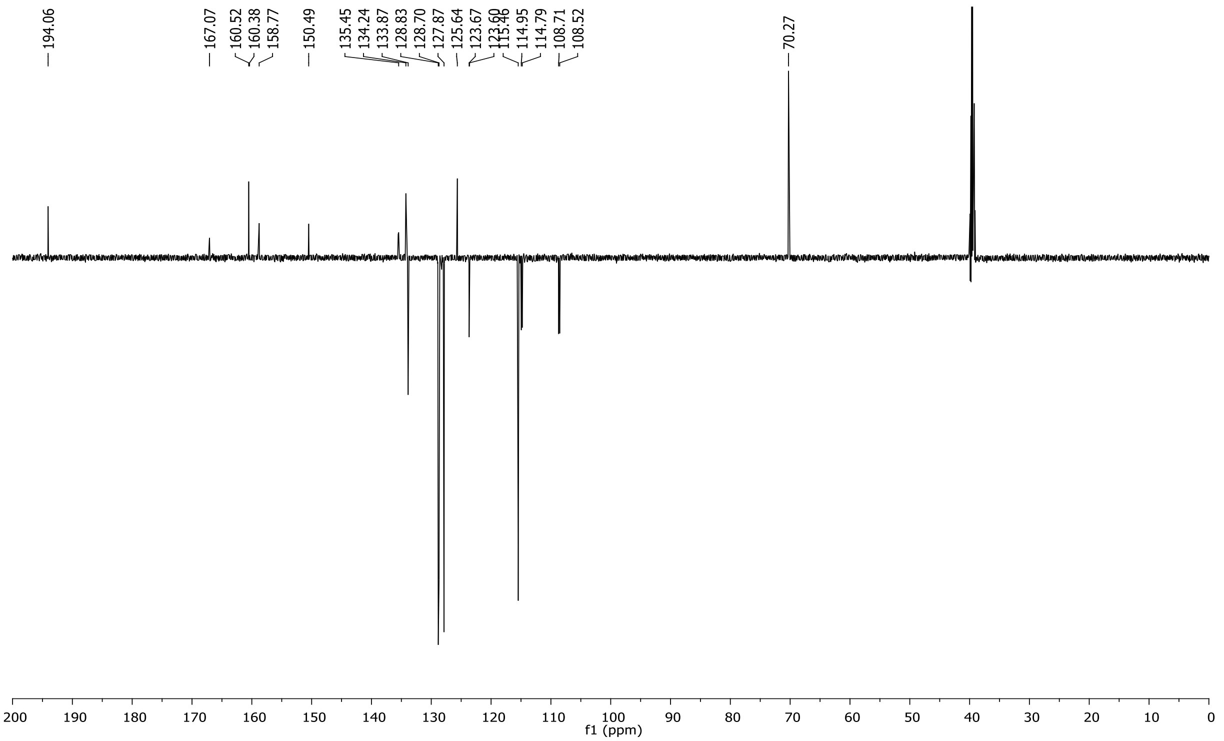

Fig. S23 a) ^{1}H NMR and b) ^{13}C NMR of compd. **16b**

a)

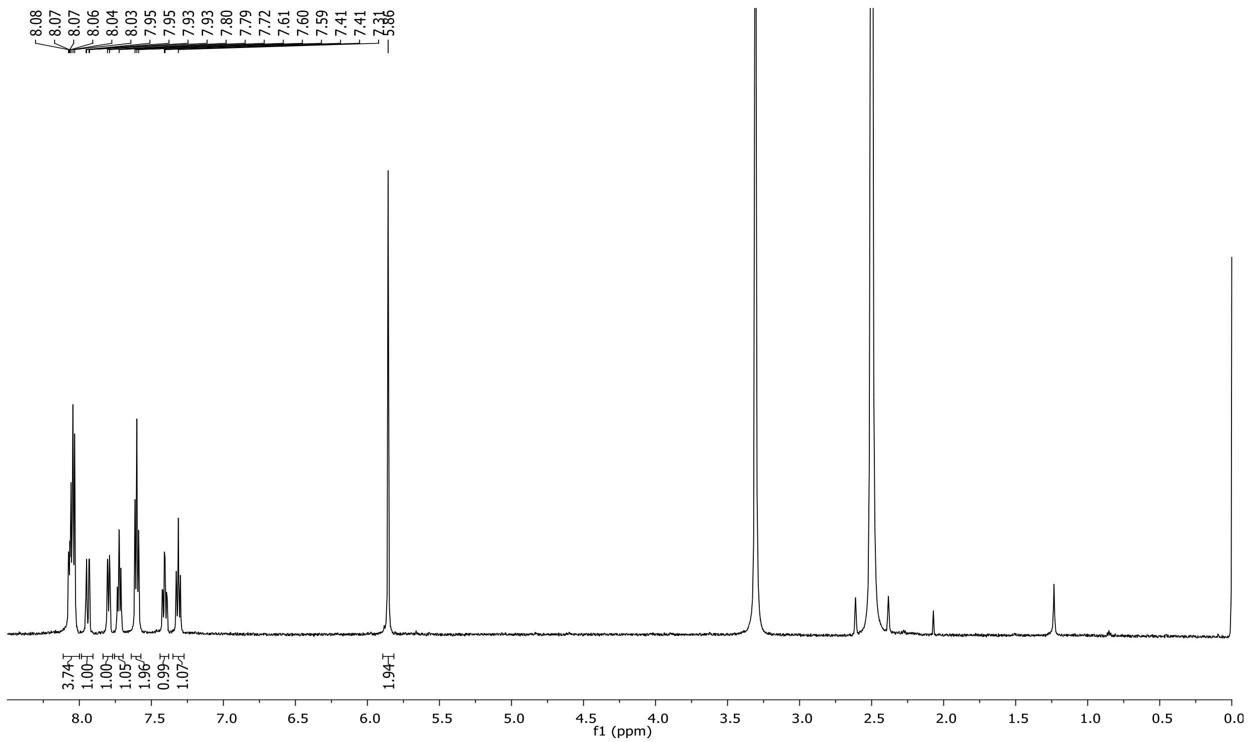

b)

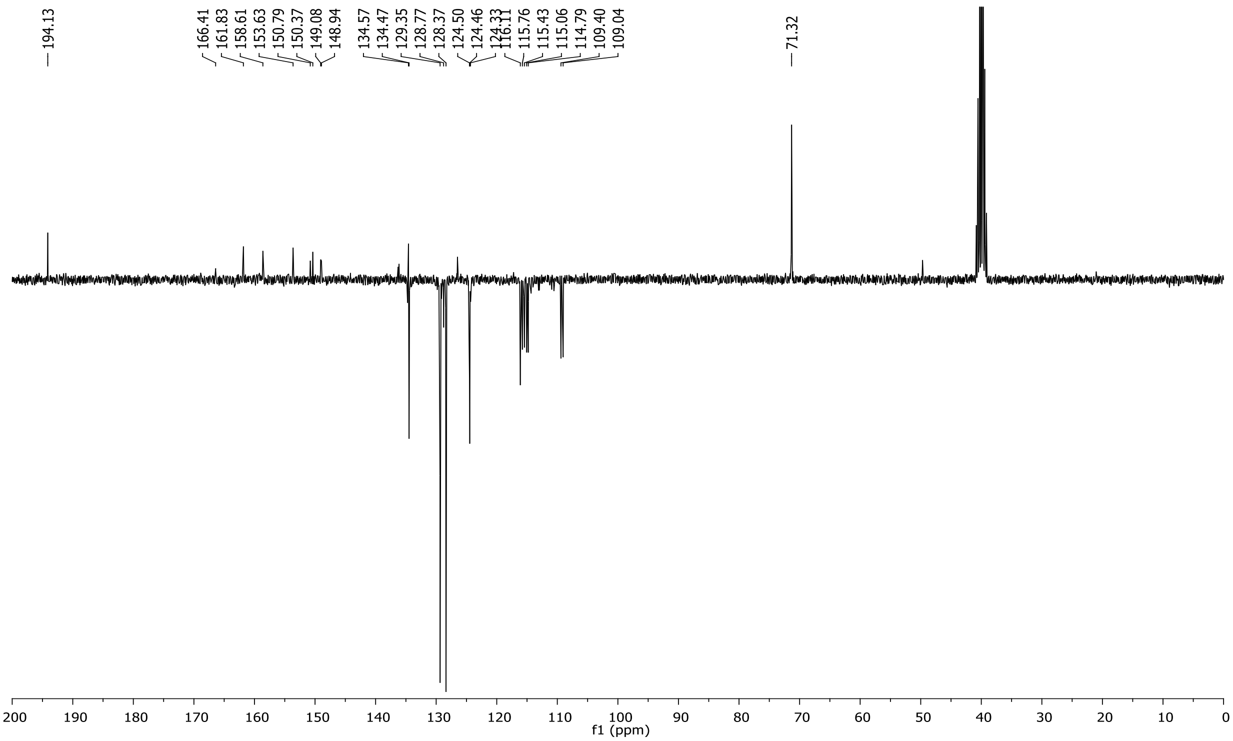

Fig. S24 a) ^{1}H NMR and b) ^{13}C NMR of compd. **16c**

a)

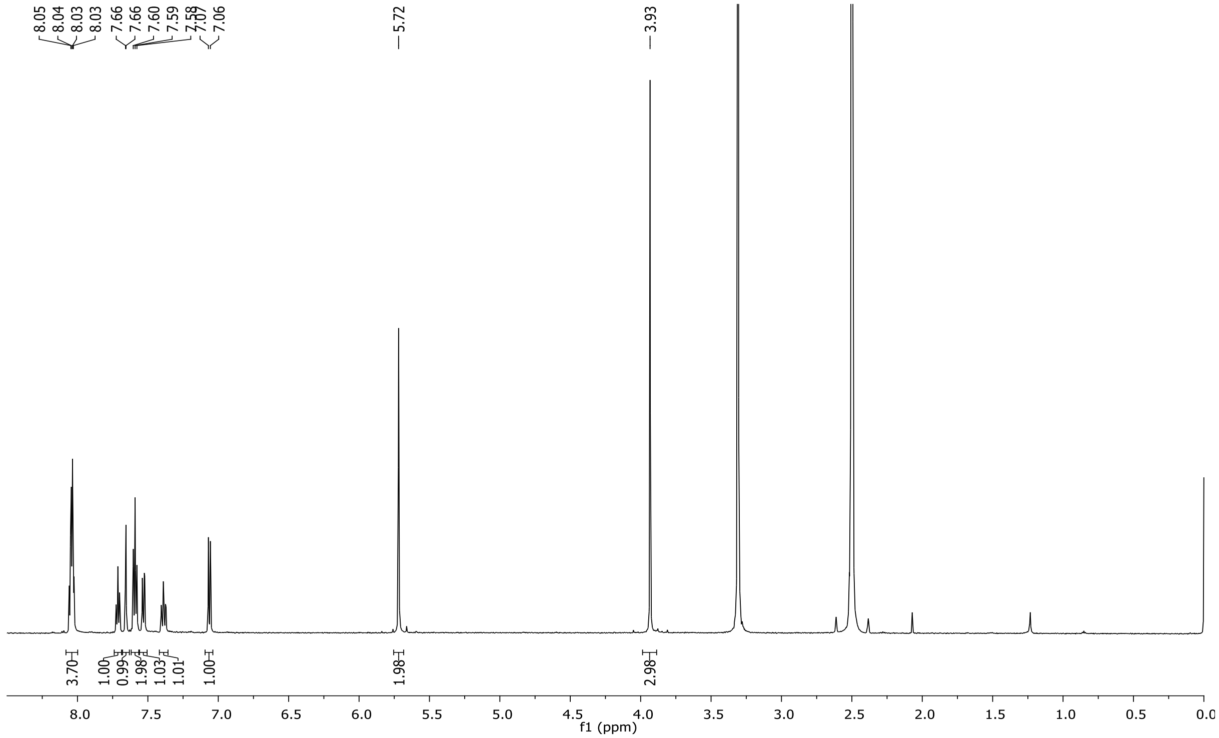

b)

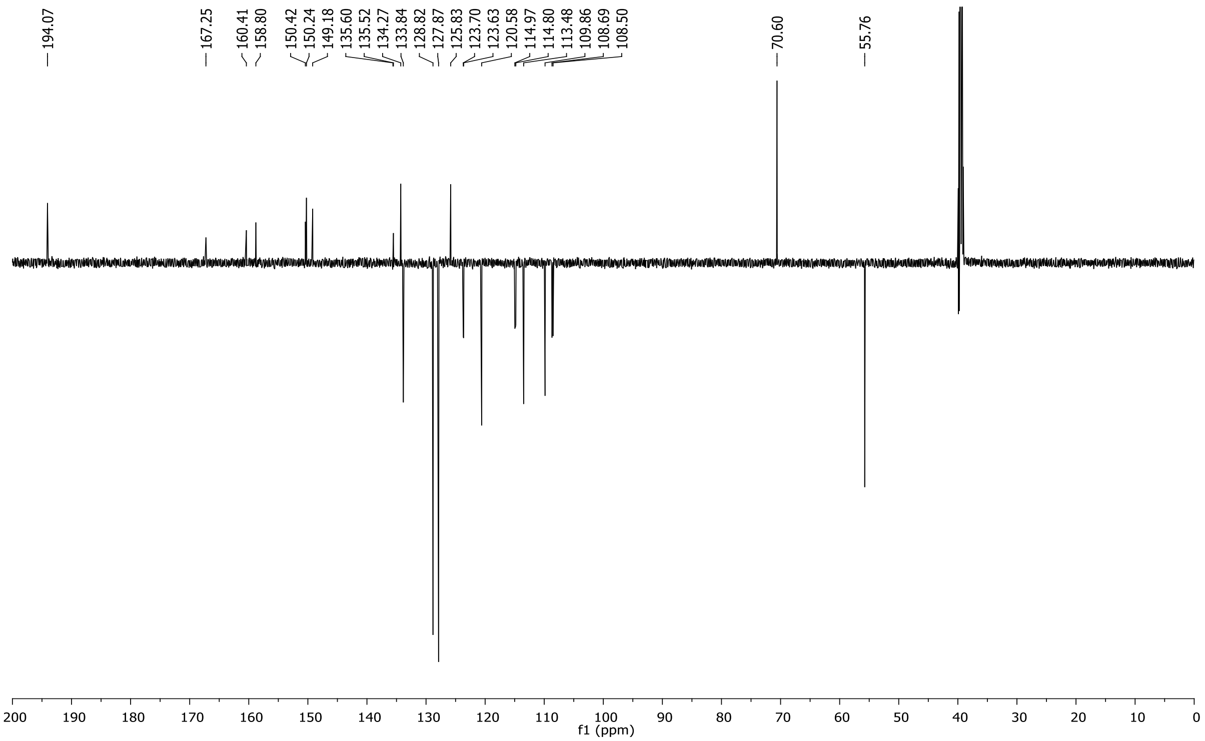

Fig. S25 a) ^{1}H NMR and b) ^{13}C NMR of compd. **17a**

a)

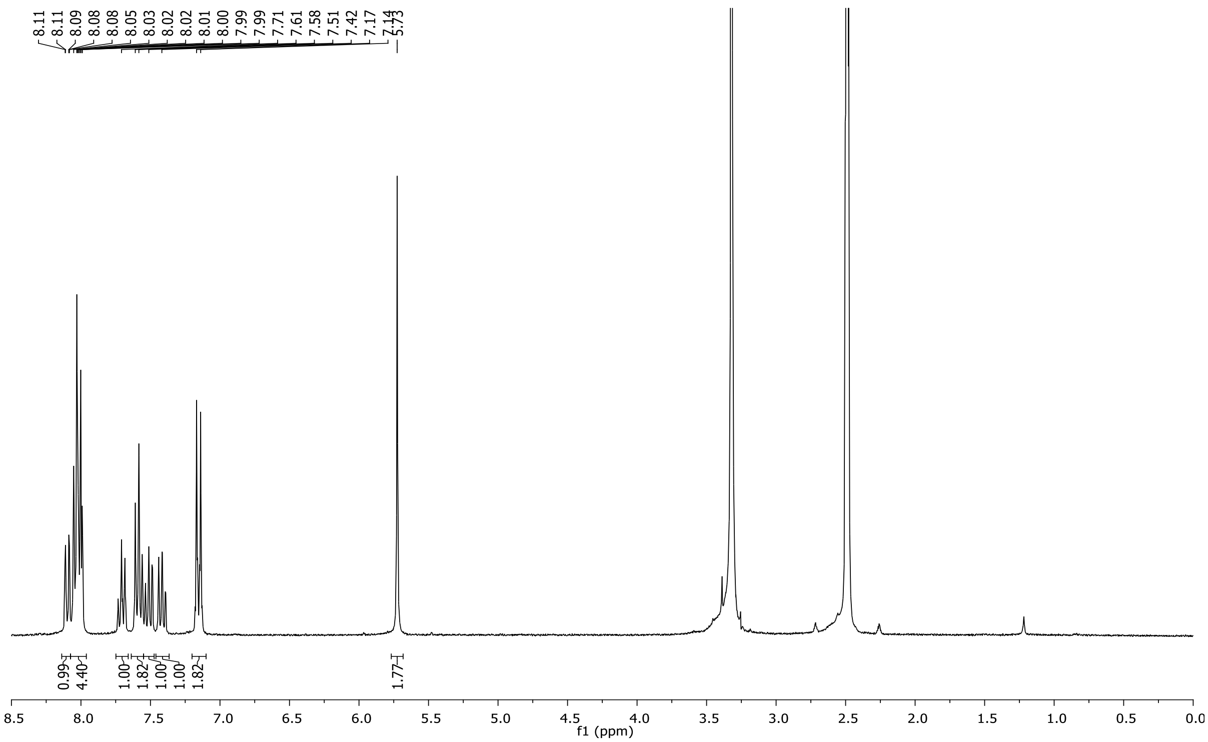

b)

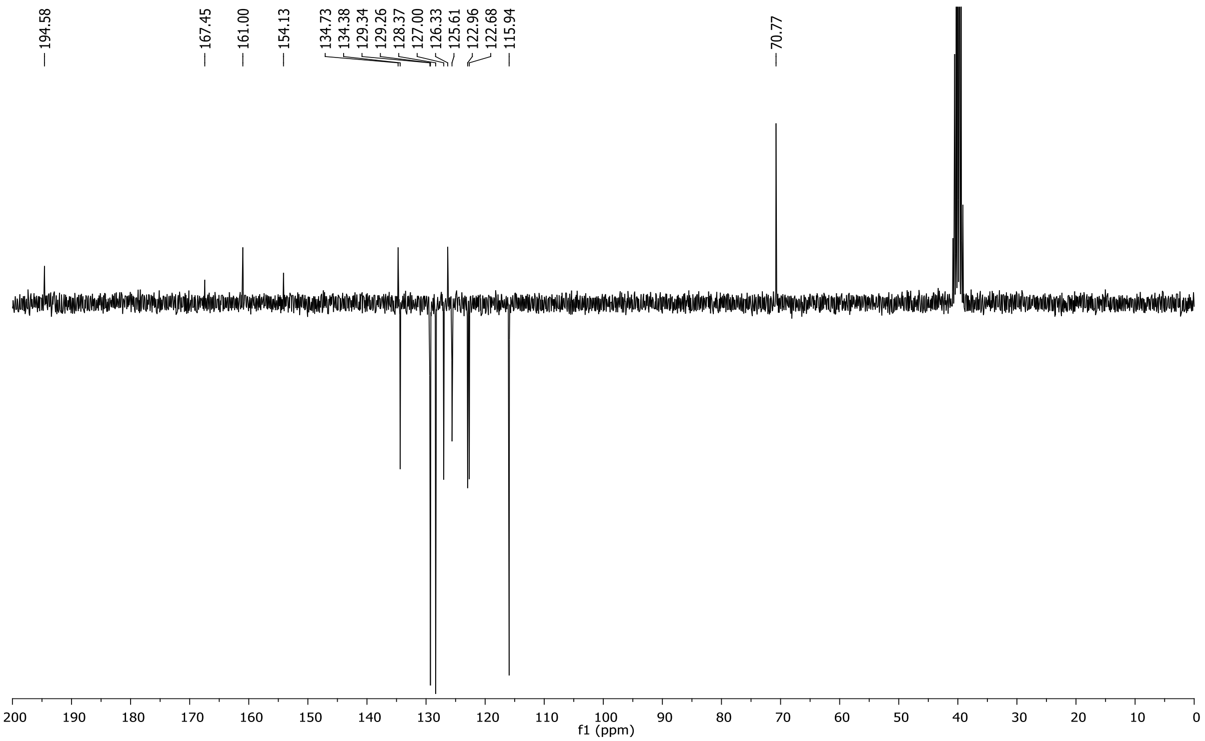

Fig. S26 a) ^{1}H NMR and b) ^{13}C NMR of compd. **17b**

a)

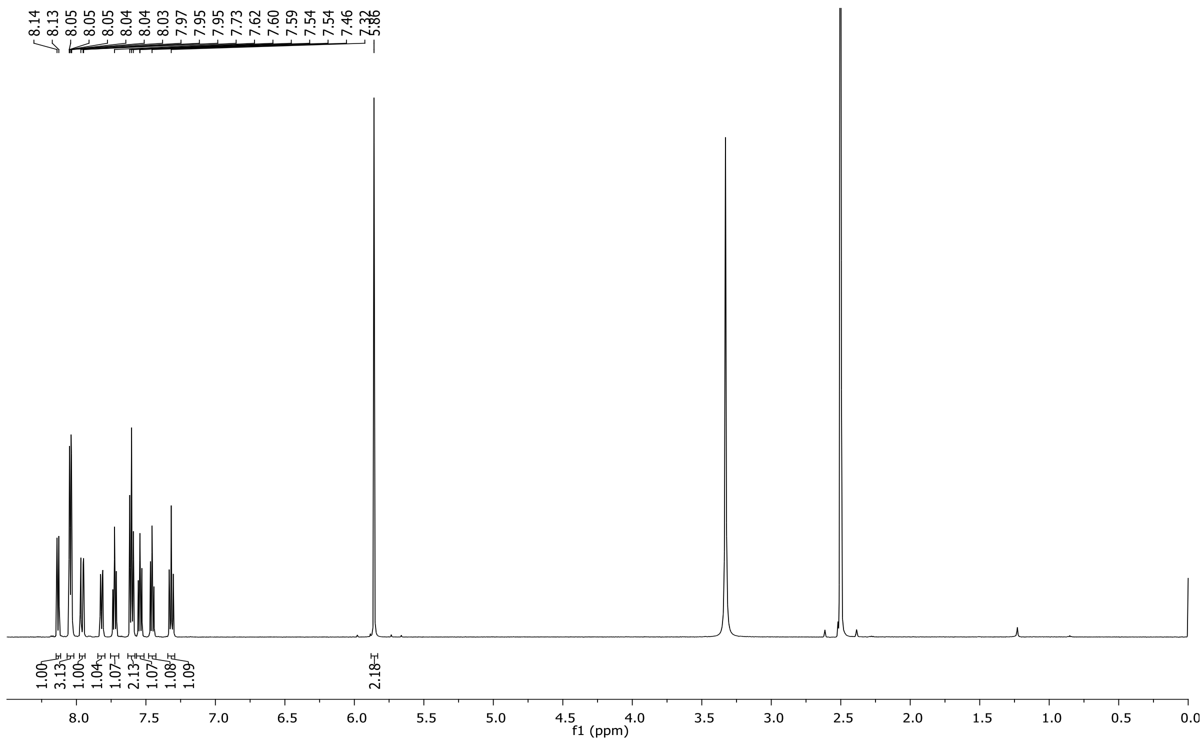

b)

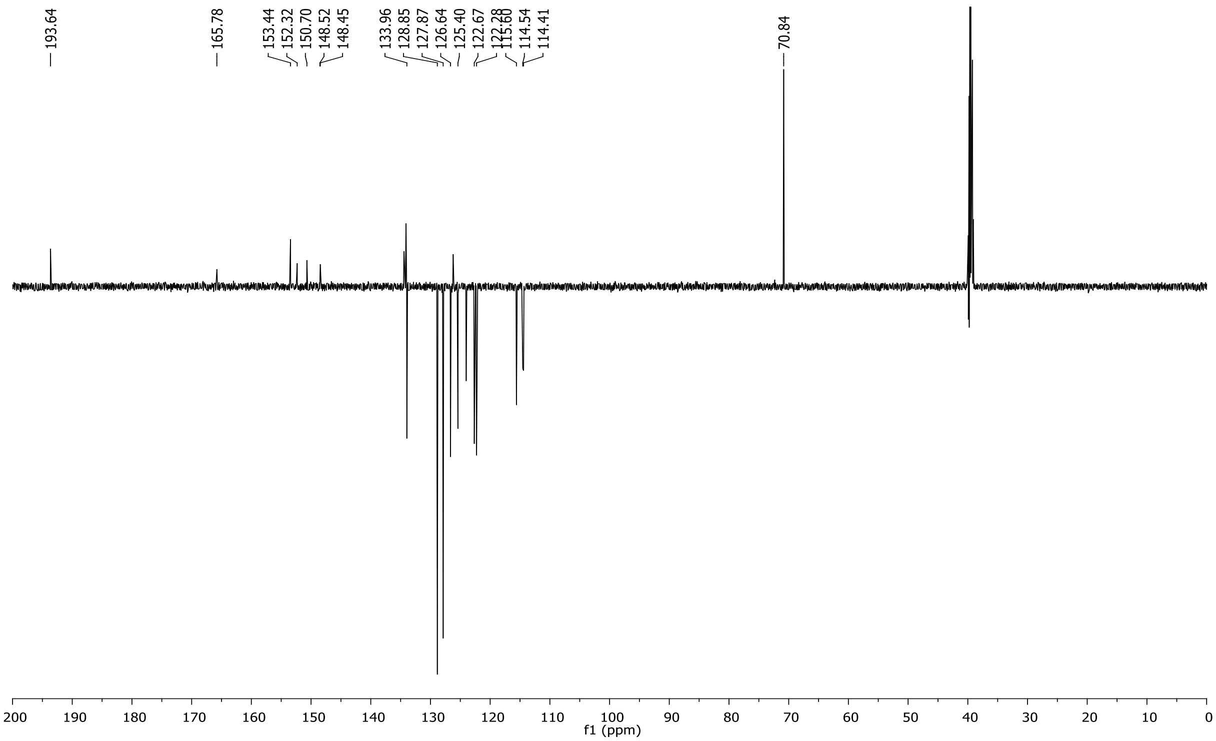

Fig. S27 a) ^{1}H NMR and b) ^{13}C NMR of compd. **17c**

a)

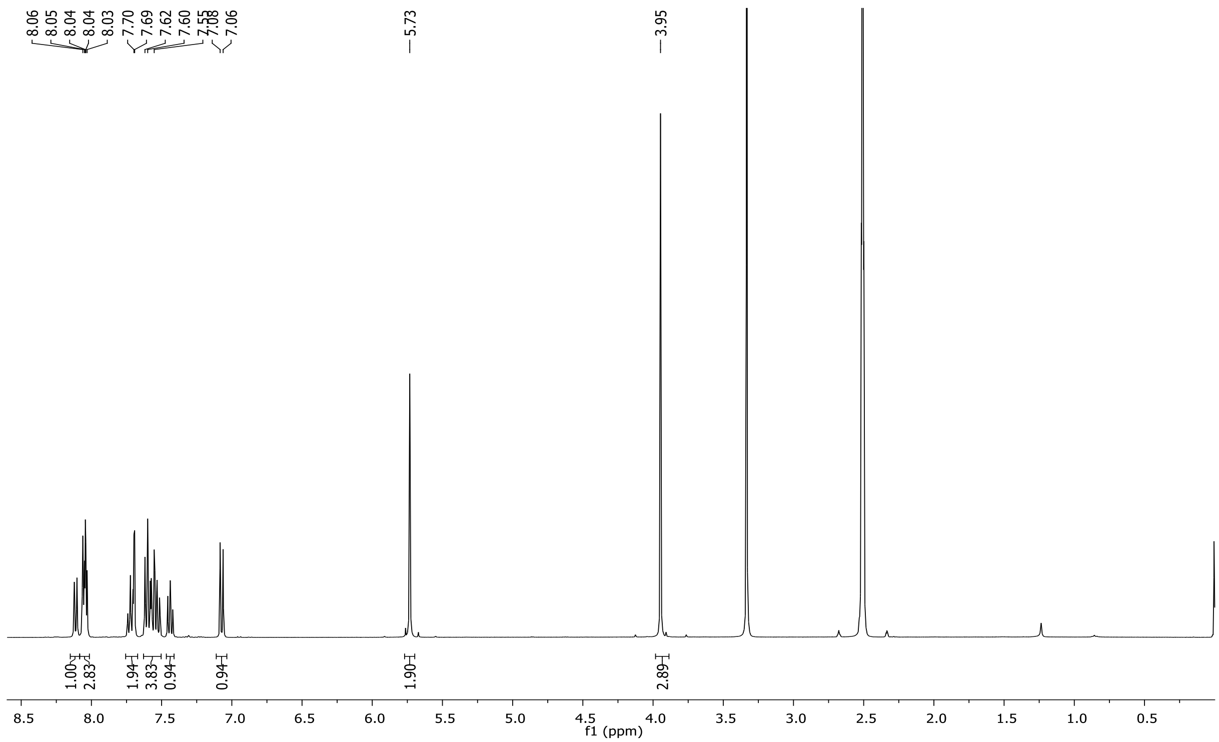

b)

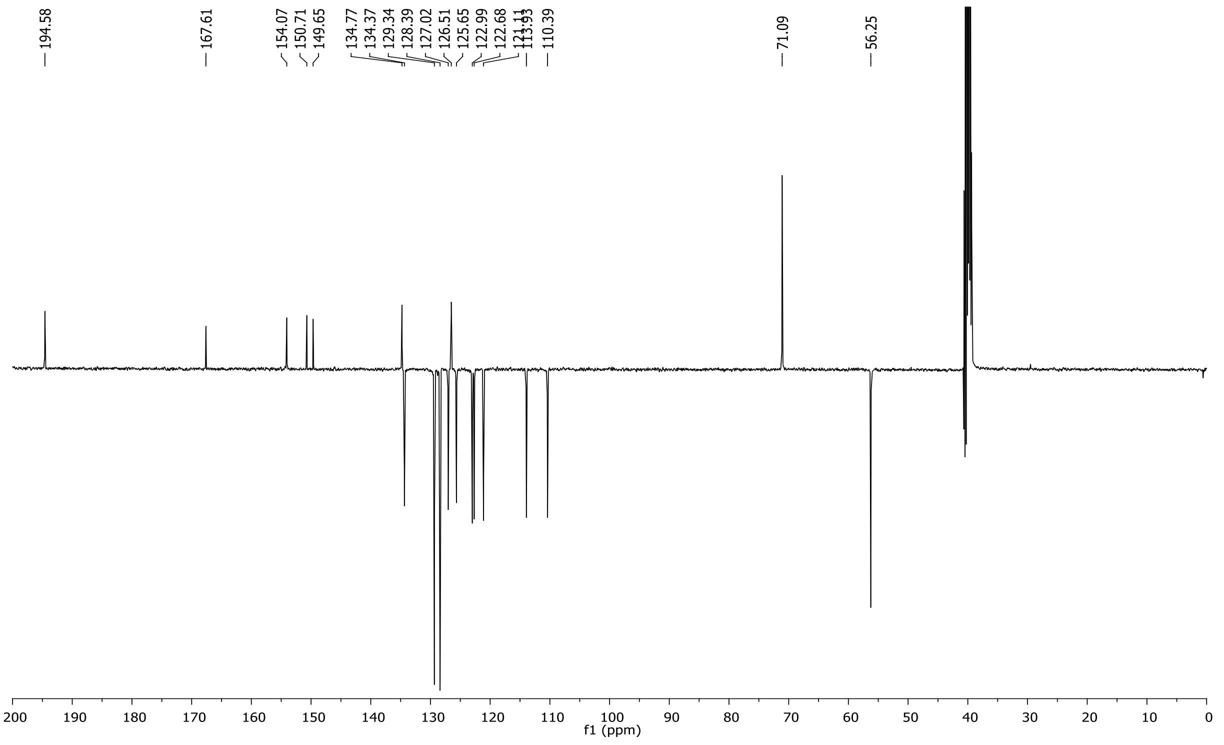

Fig. S28 a) ^{1}H NMR and b) ^{13}C NMR of compd. **18a**

a)

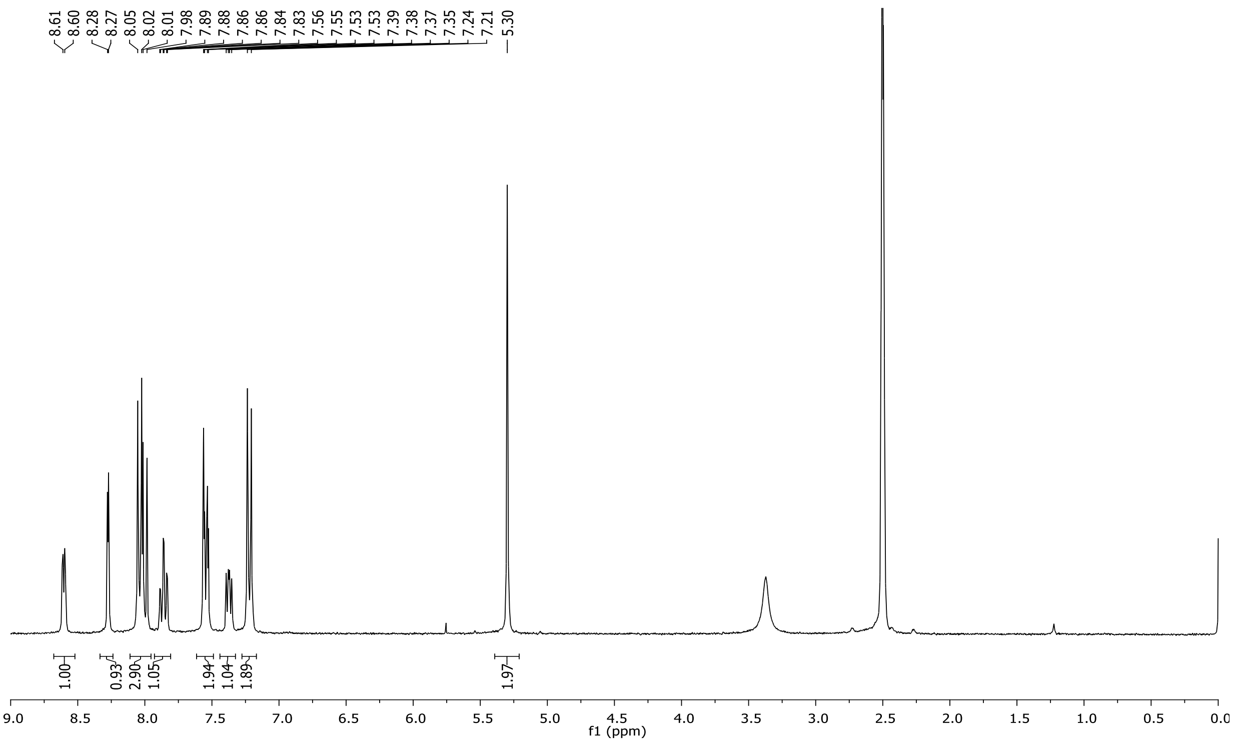

b)

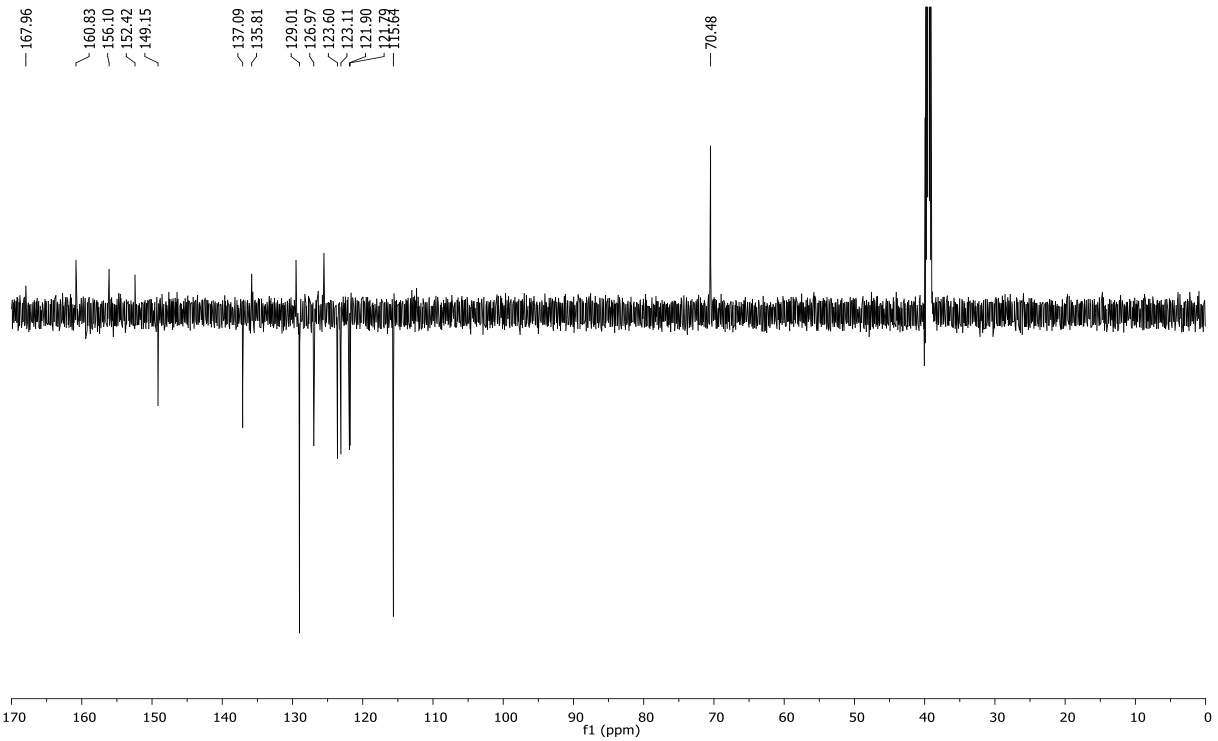

Fig. S29 a) ^{1}H NMR and b) ^{13}C NMR of compd. **18b**

a)

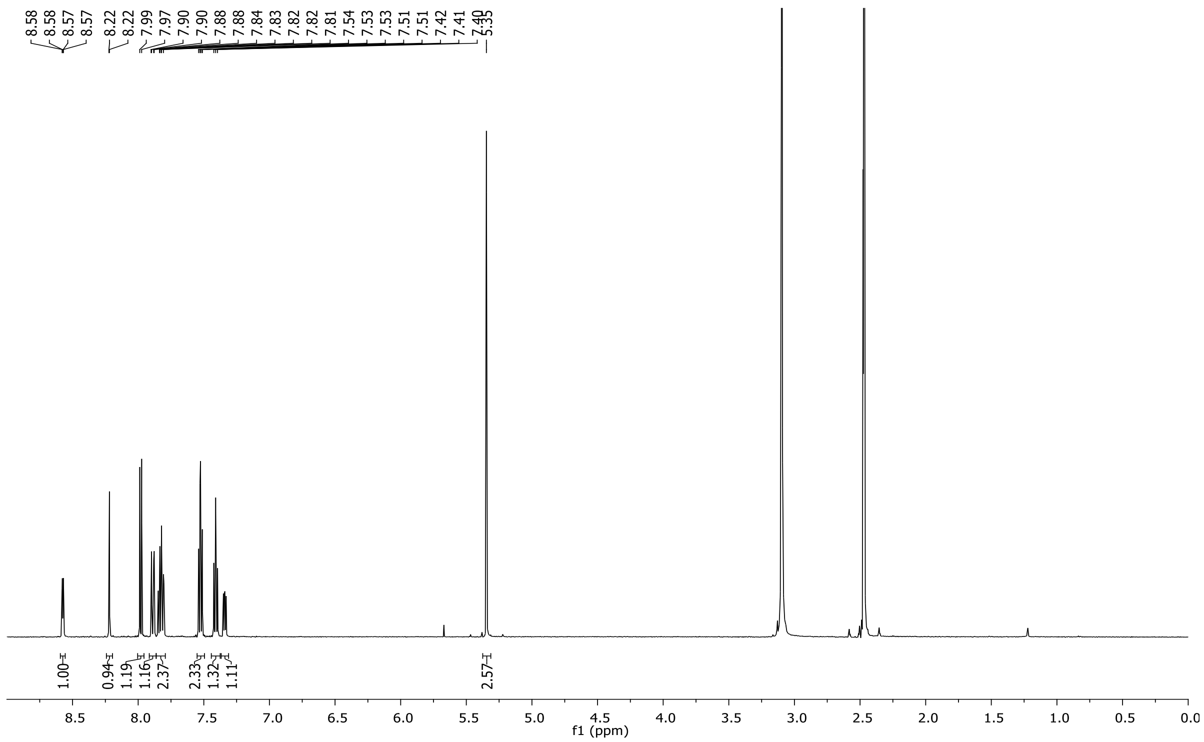

b)

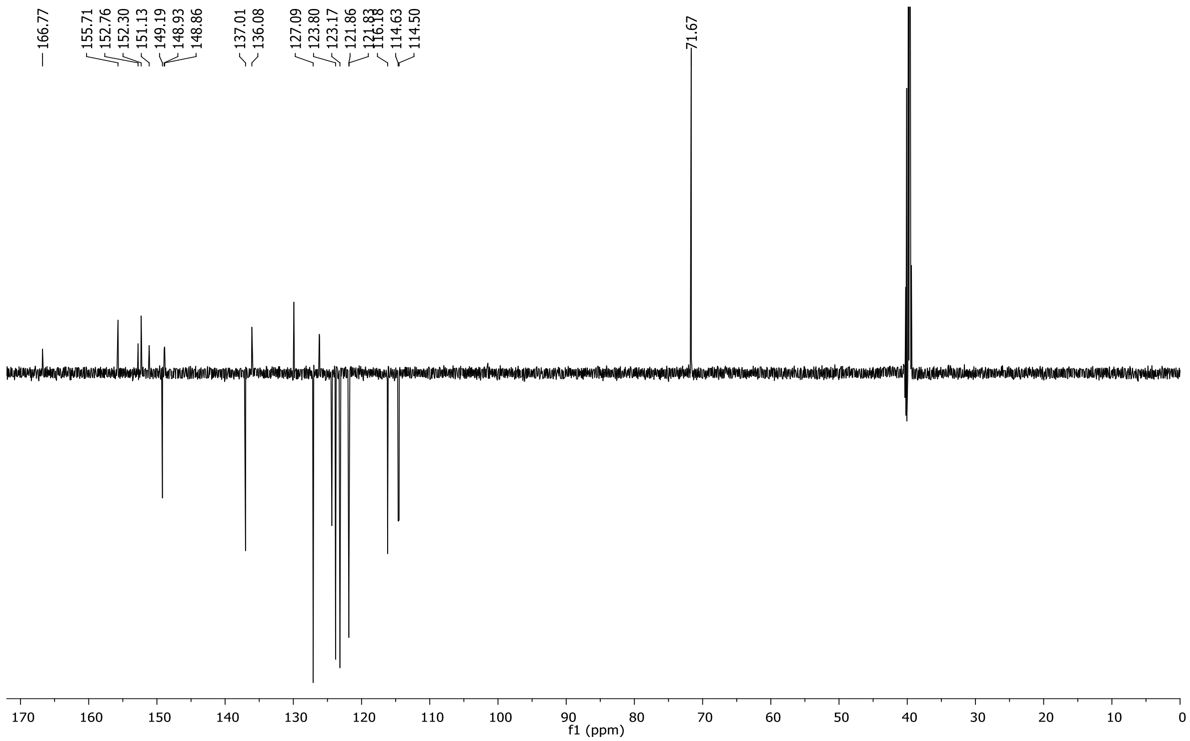

Fig. S30 a) ^{1}H NMR and b) ^{13}C NMR of compd. **18c**

a)

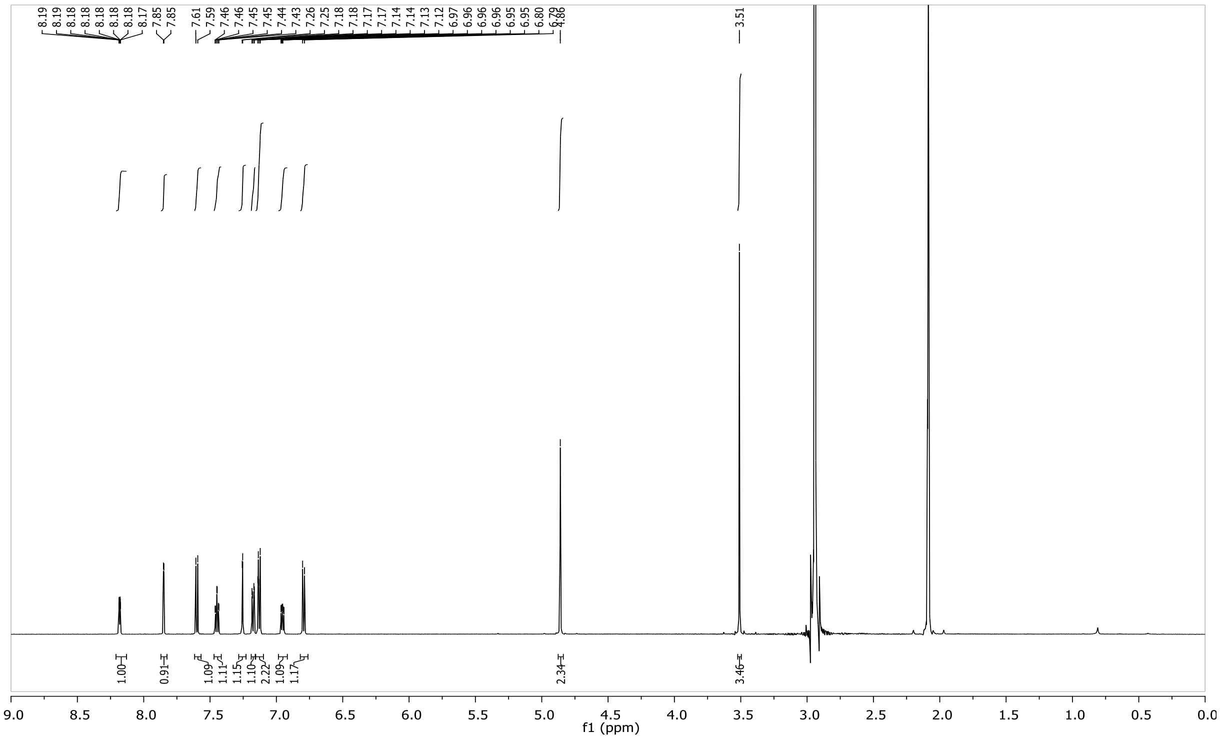

b)

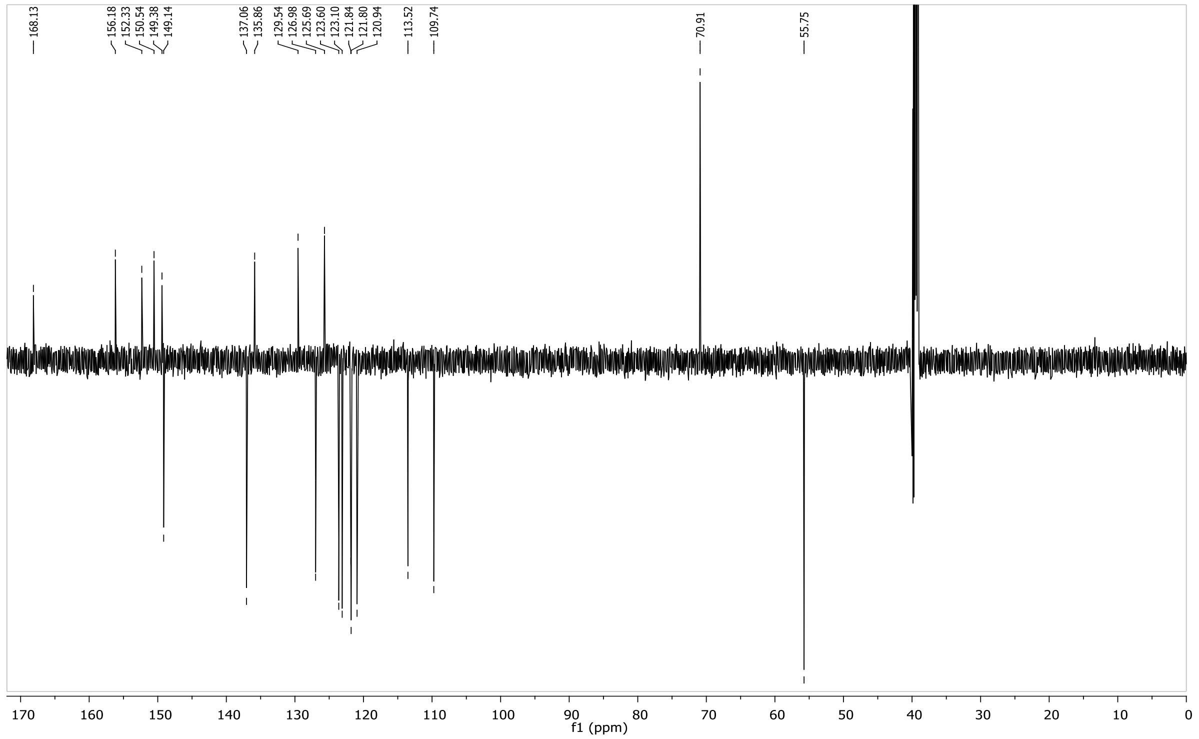

Fig. S31 a) ^{1}H NMR and b) ^{13}C NMR of compd. **19a**

a)

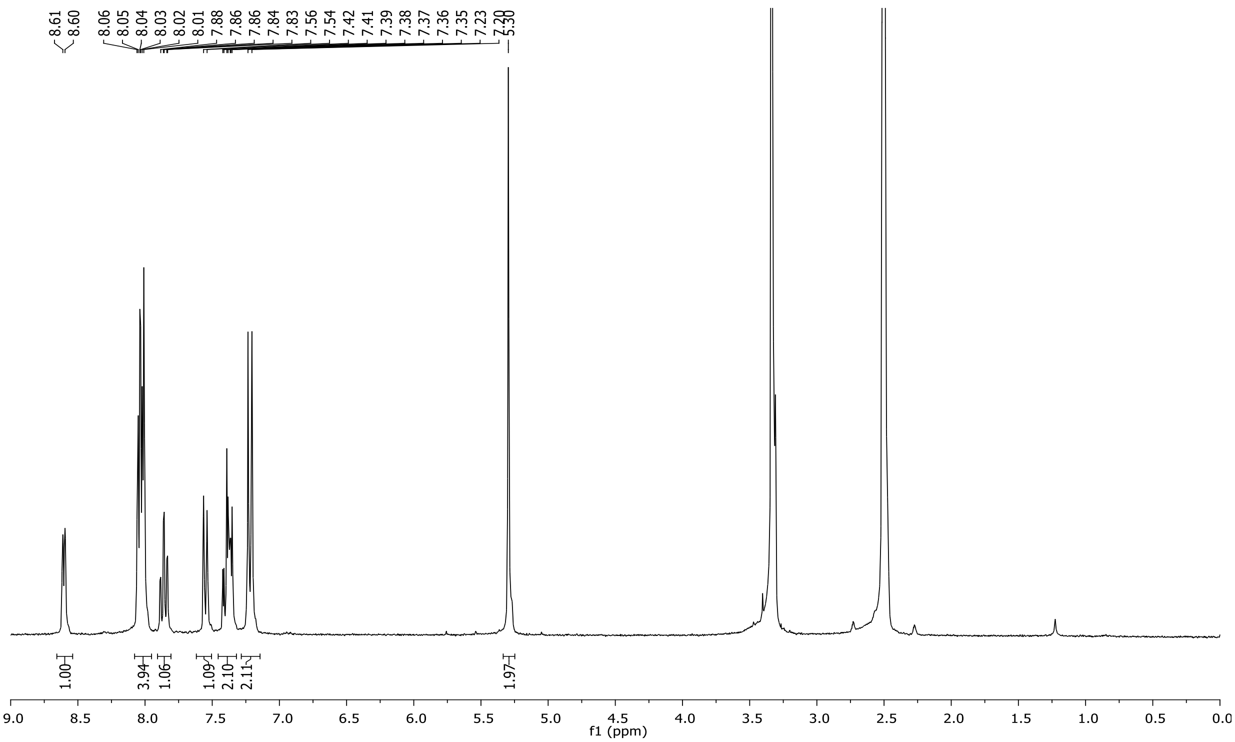

b)

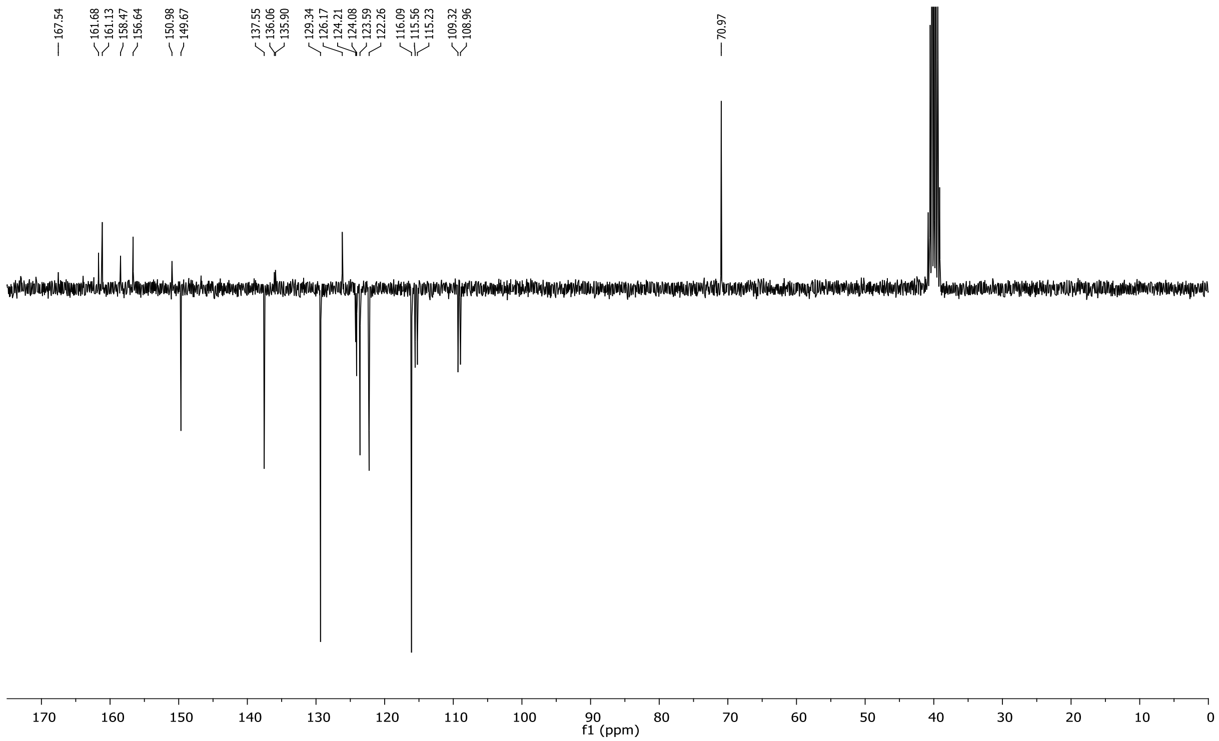

Fig. S32 a) ^{1}H NMR and b) ^{13}C NMR of compd. **19b**

a)

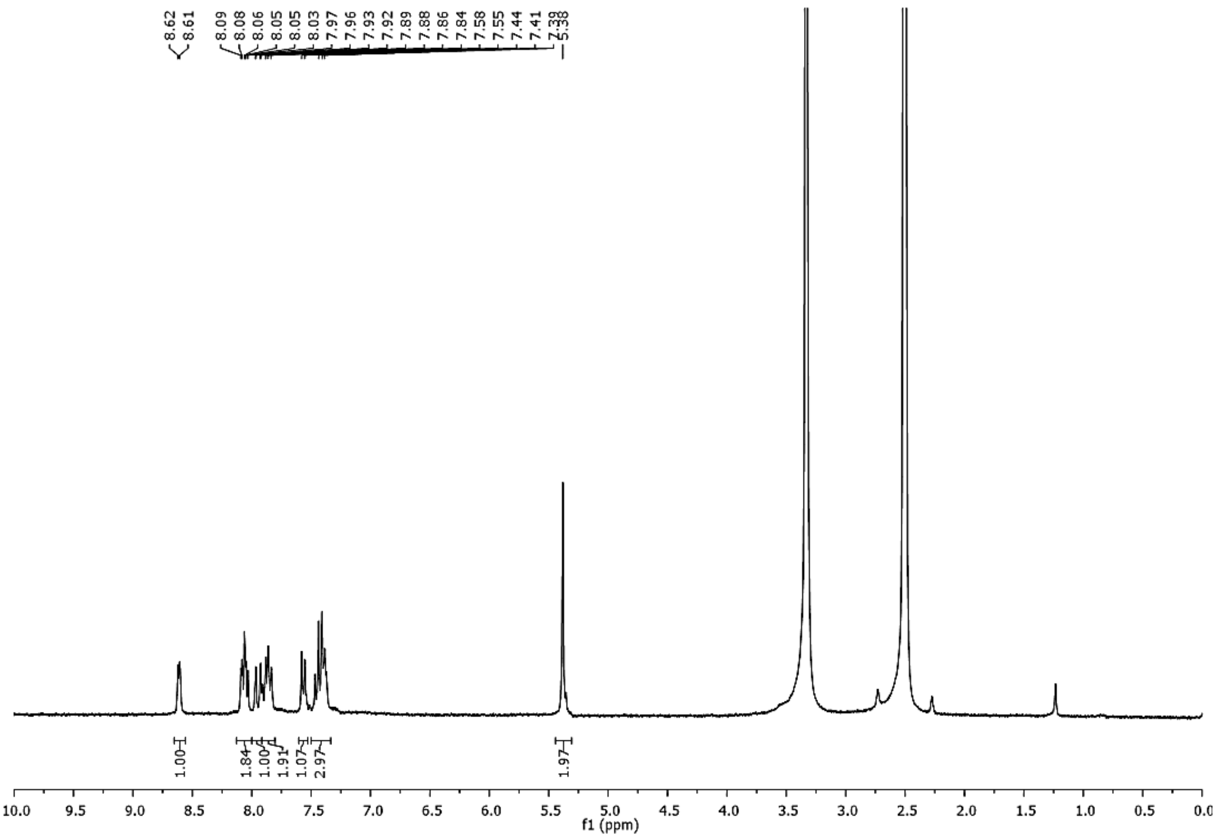

b)

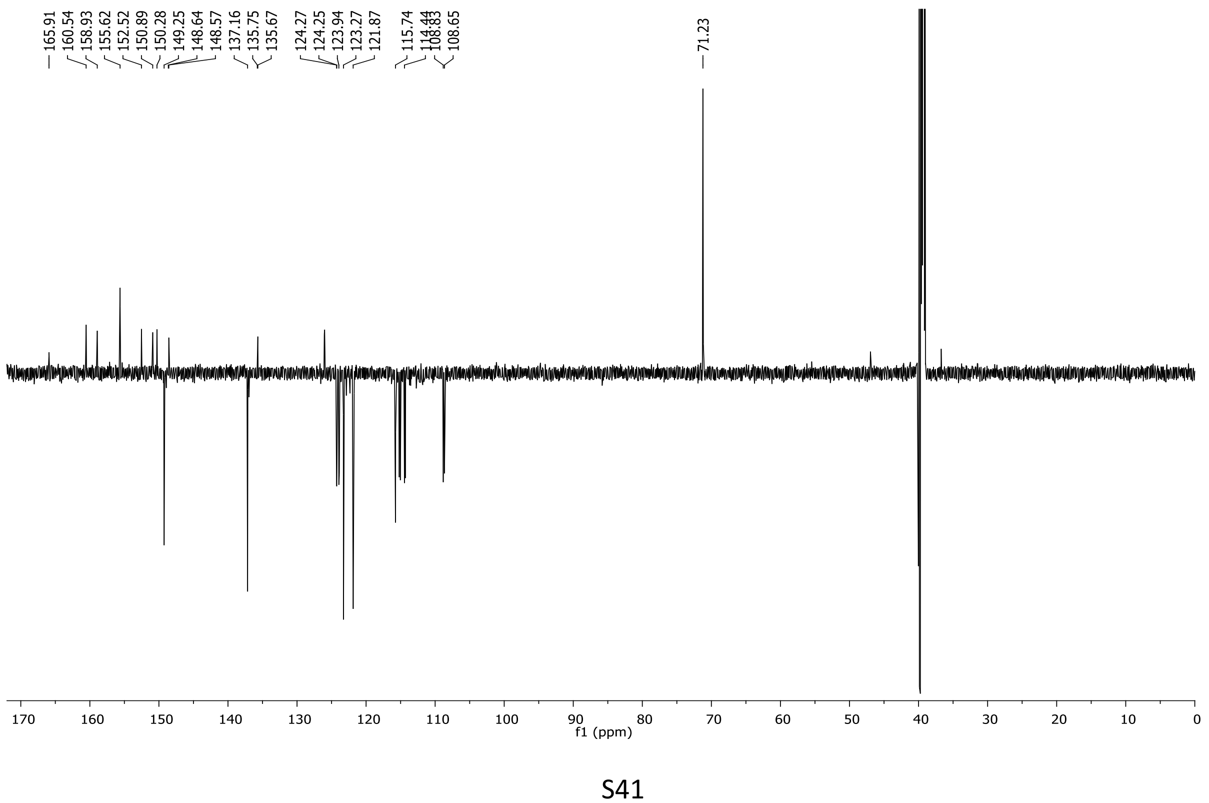

Fig. S33 a) ^{1}H NMR and b) ^{13}C NMR of compd. **19c**

a)

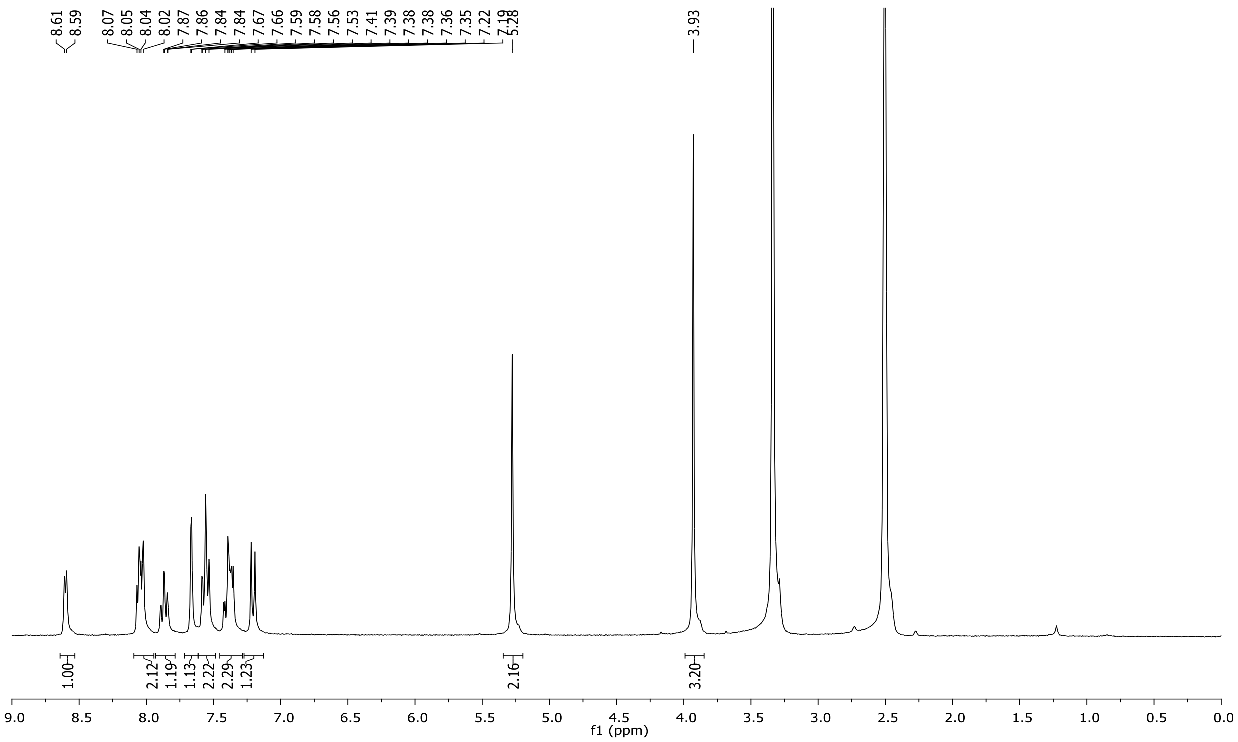

b)

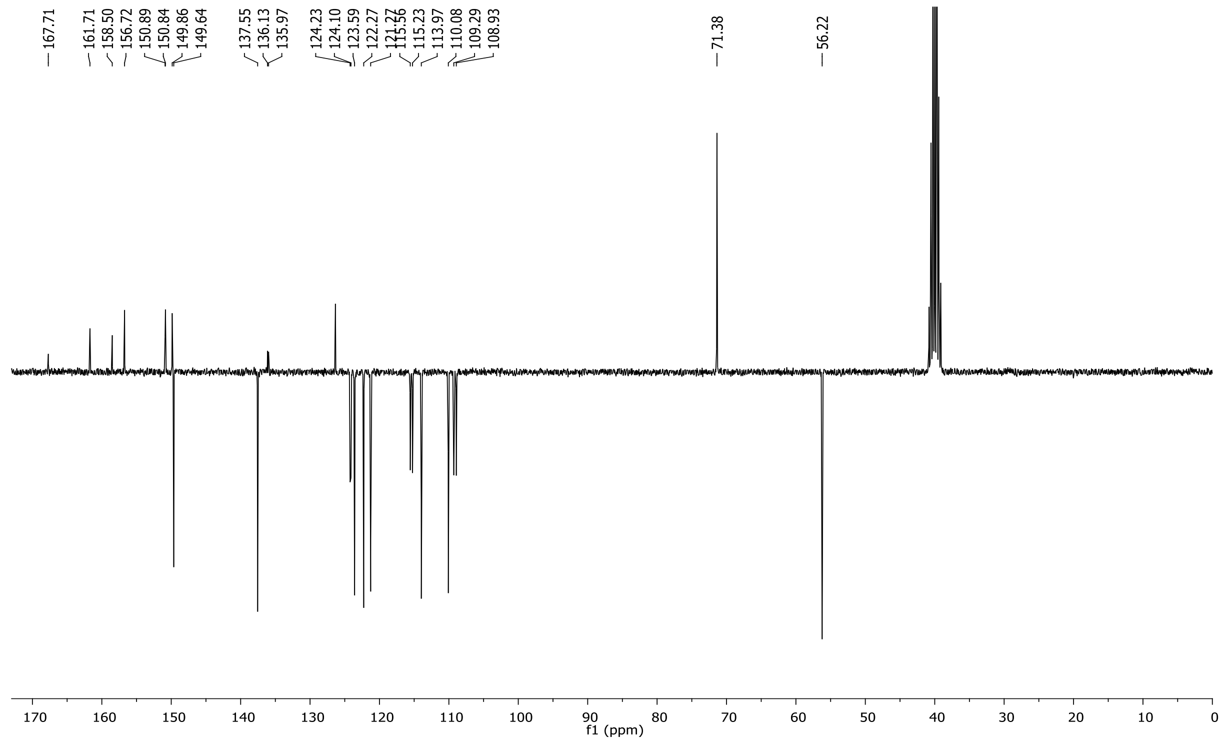

Fig. S34 a) ^{1}H NMR and b) ^{13}C NMR of compd. **20a**

a)

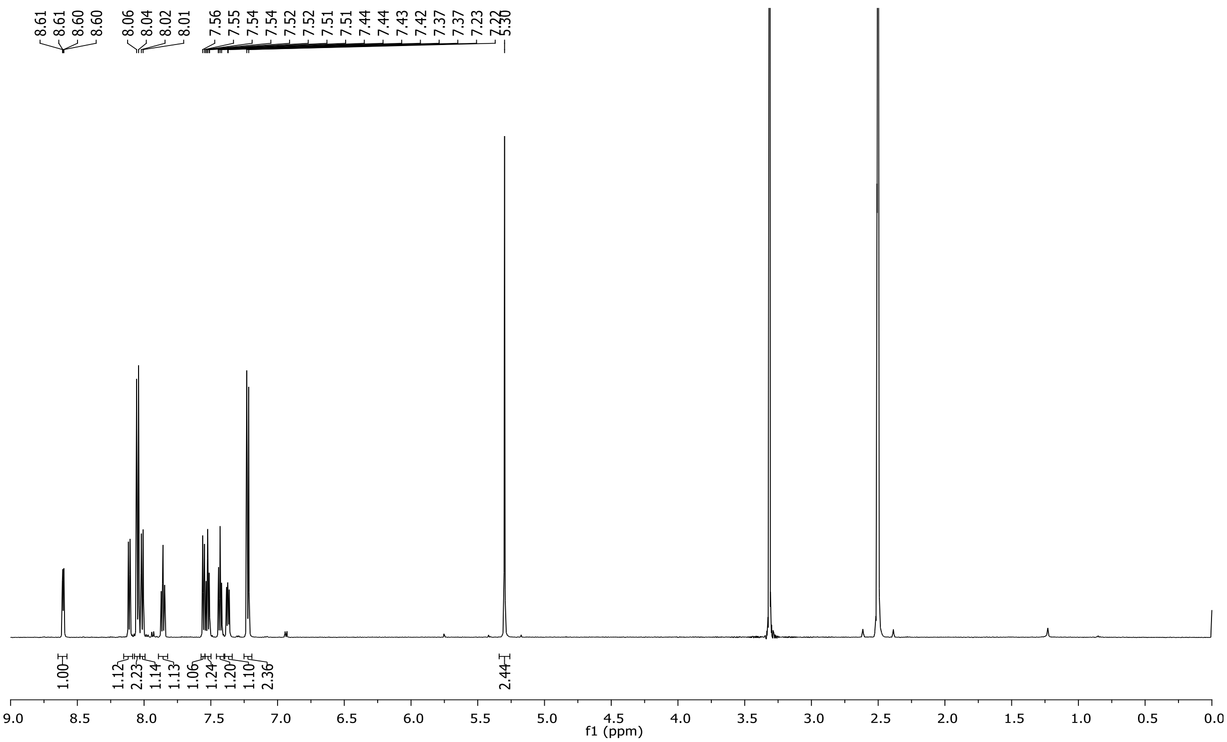

b)

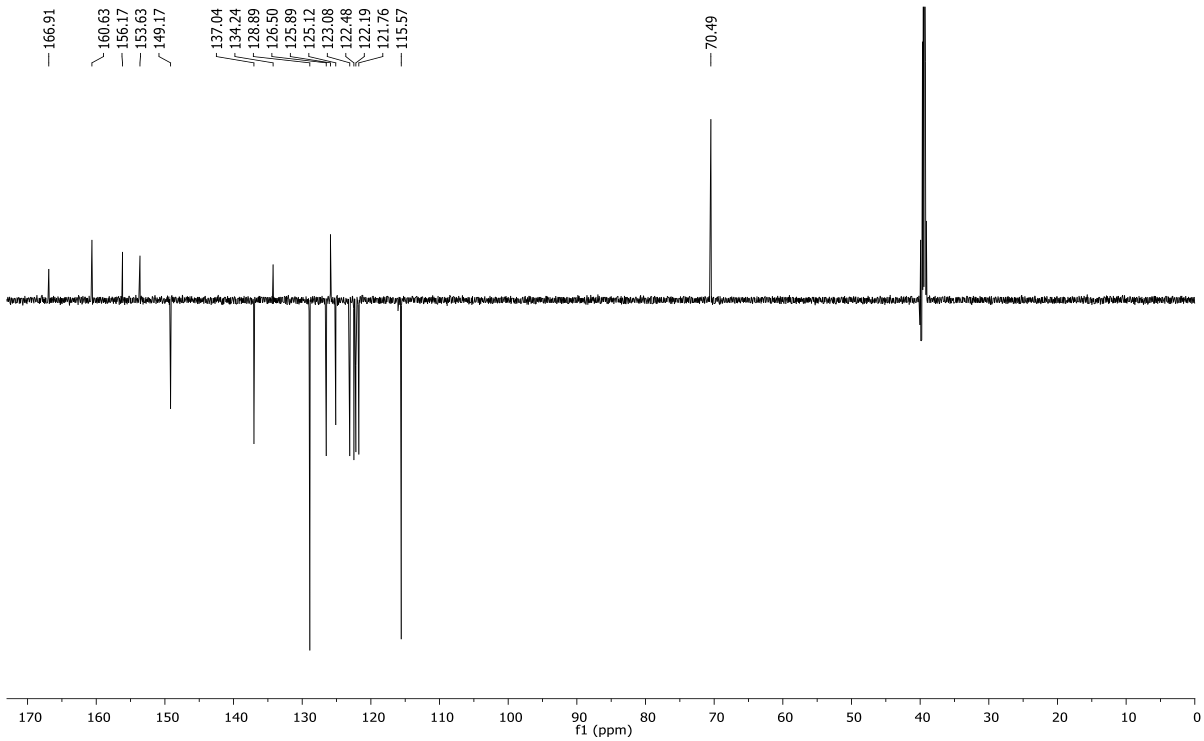

Fig. S35 a) ^{1}H NMR and b) ^{13}C NMR of compd. **20b**

a)

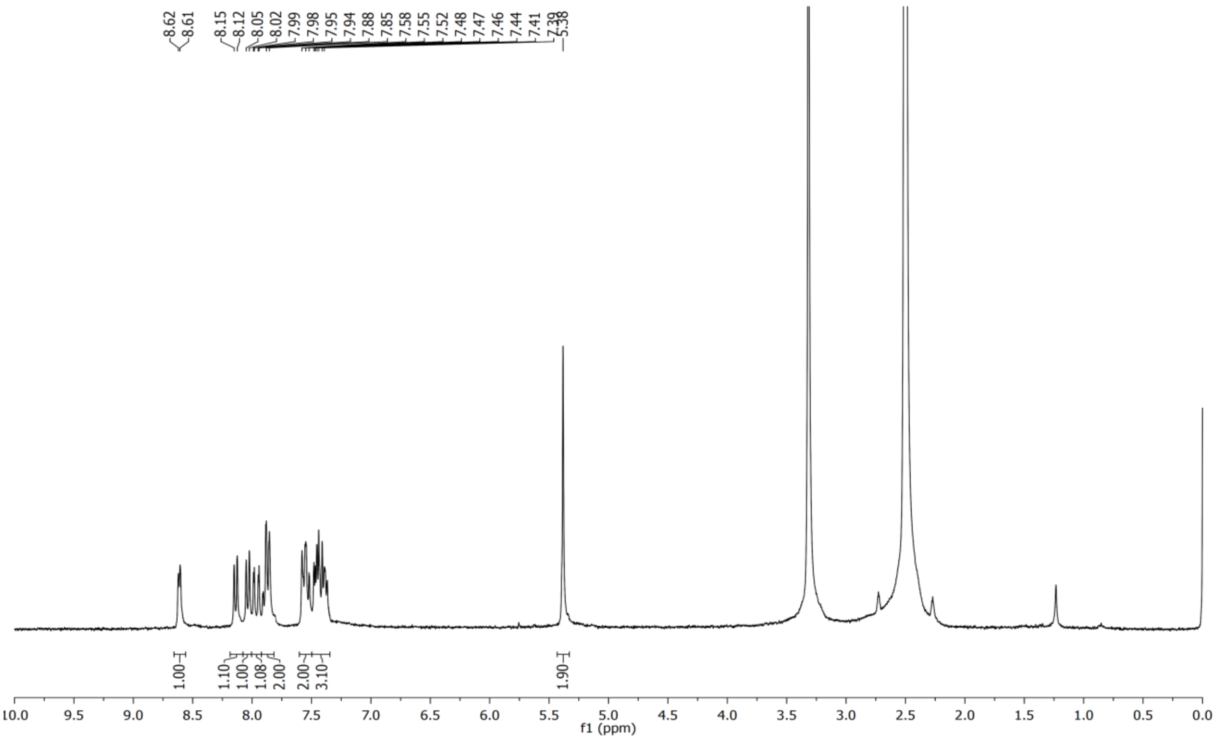

b)

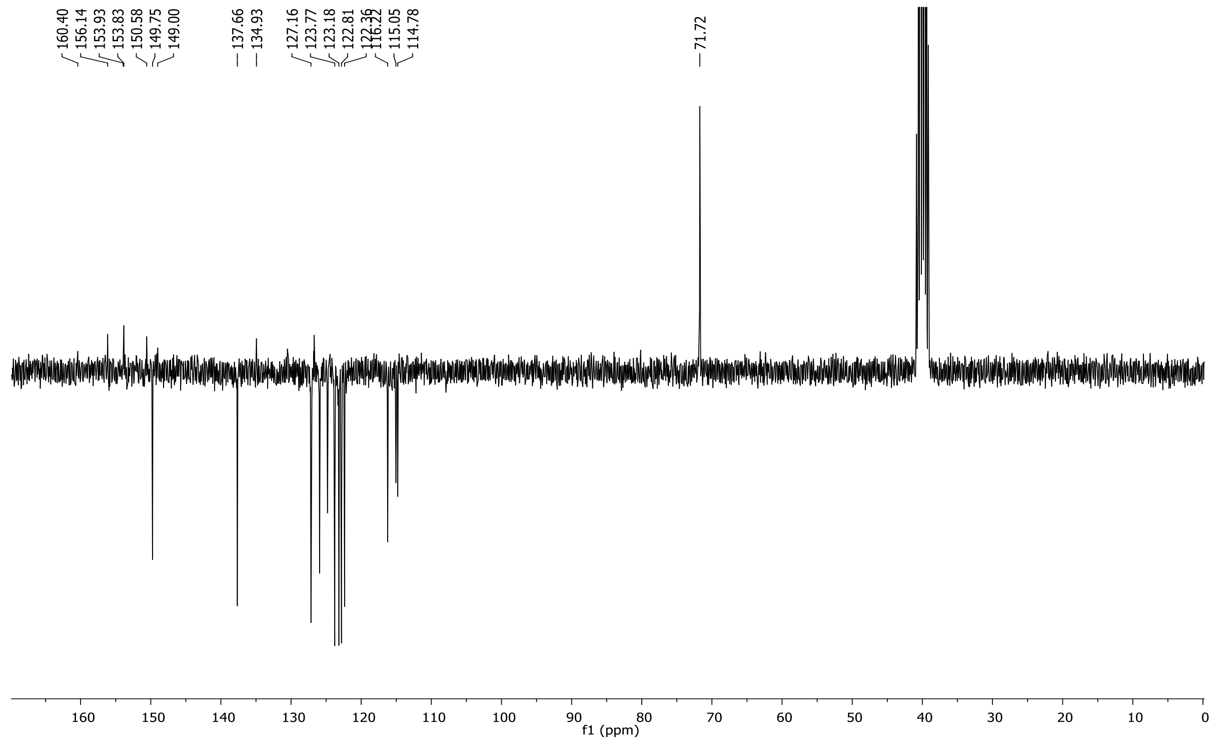

Fig. S36 a) ^{1}H NMR and b) ^{13}C NMR of compd. **20c**

a)

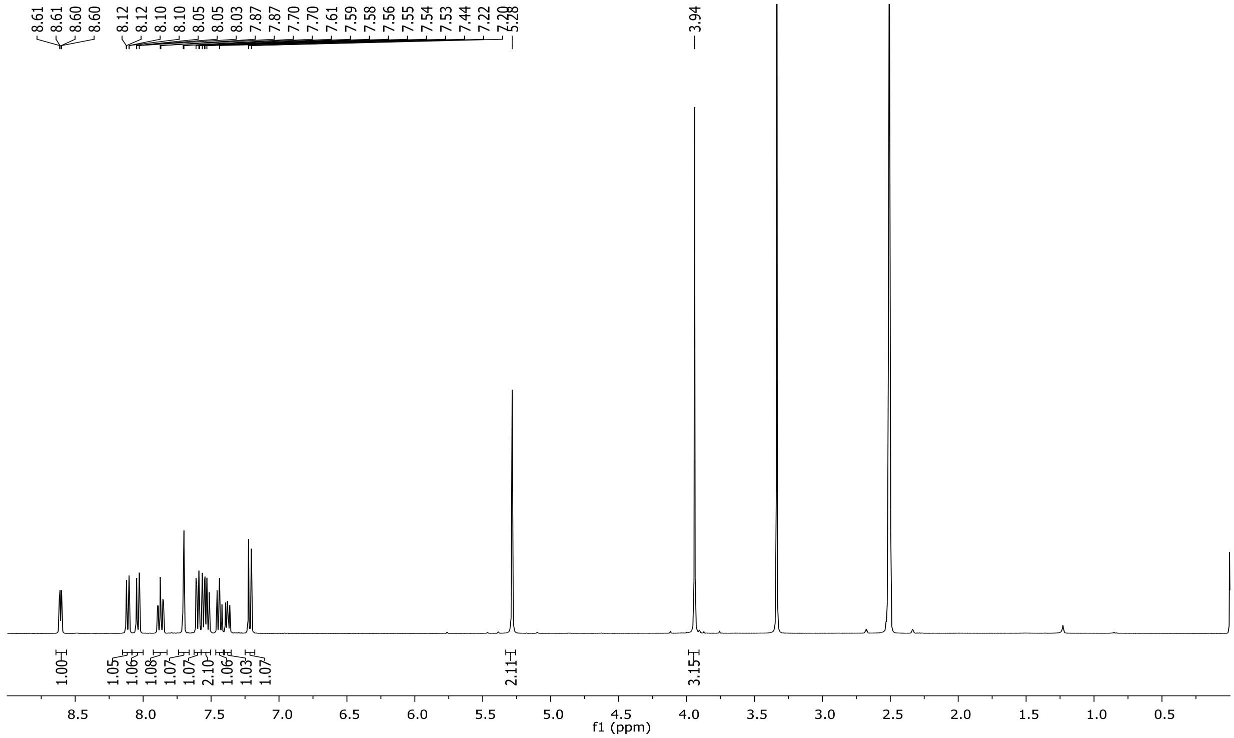

b)

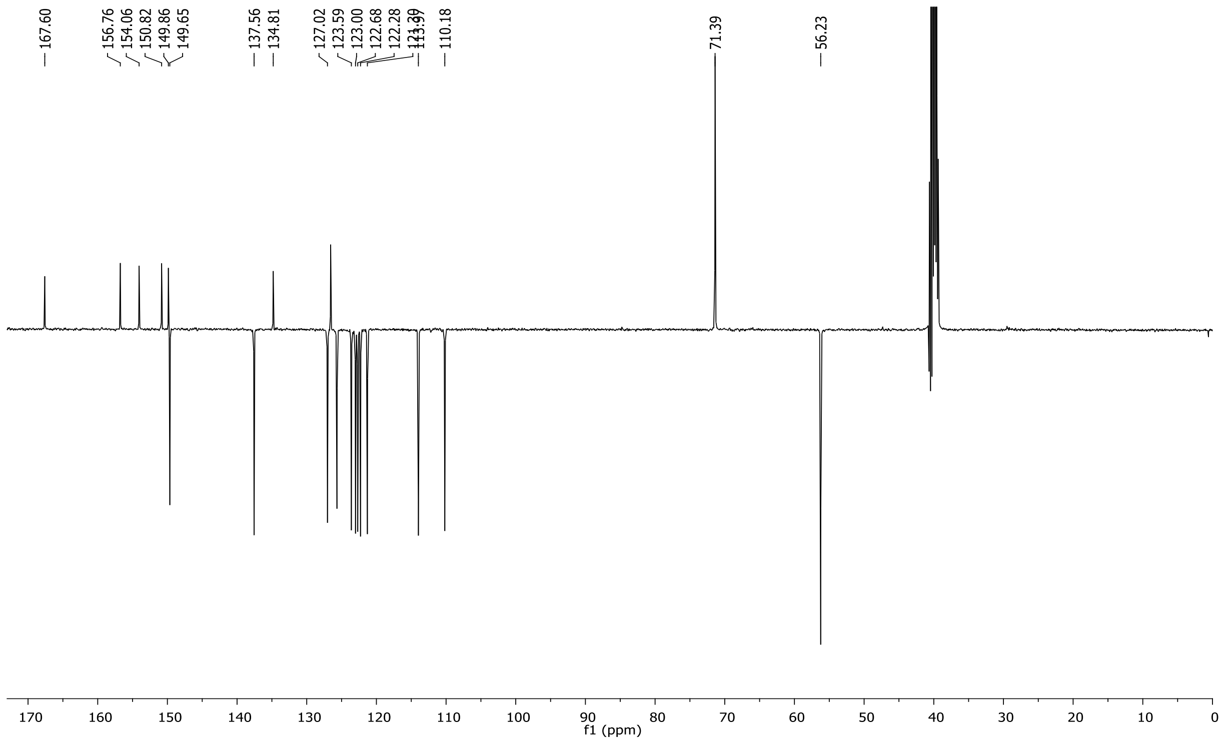

Fig. S37 a) ^{1}H NMR and b) ^{13}C NMR of compd. **21a**

a)

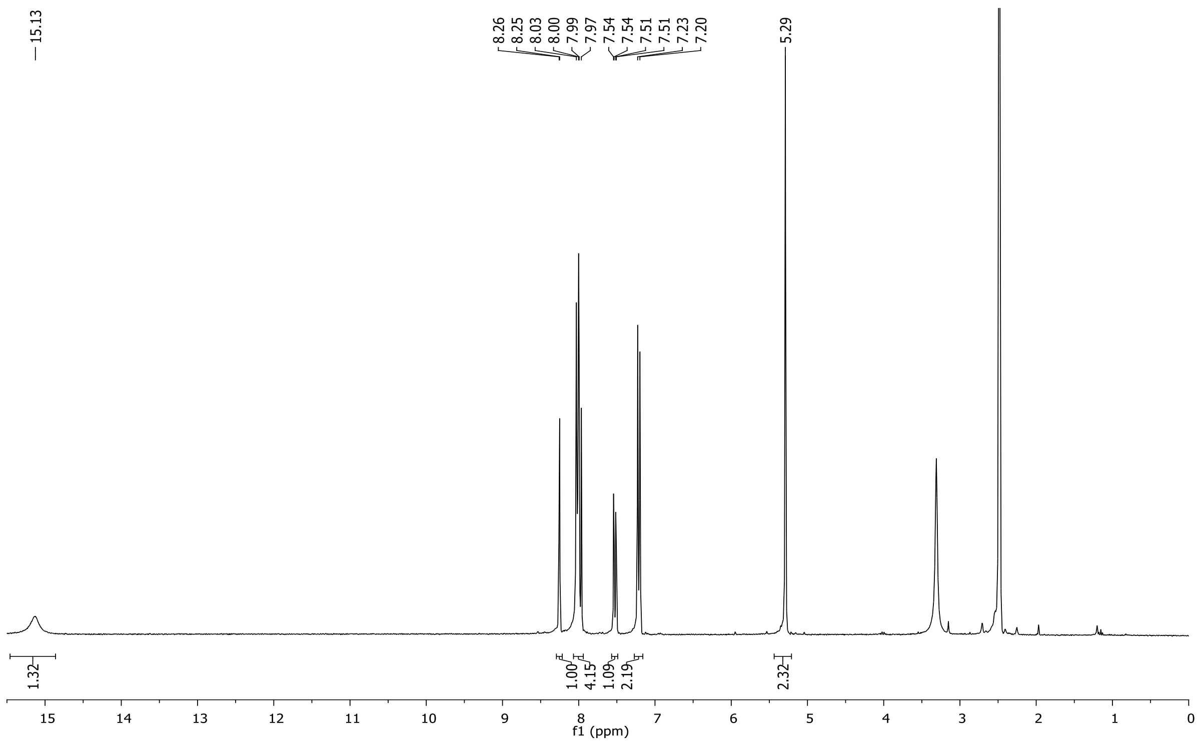

b)

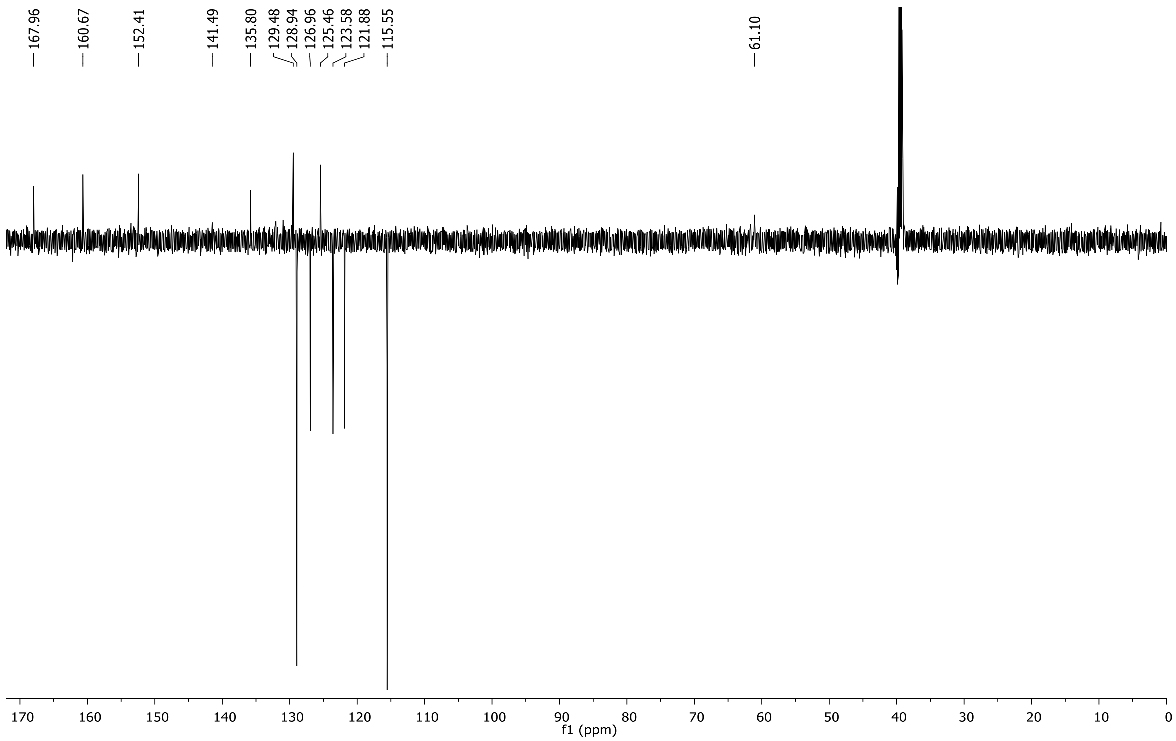

Fig. S38 a) ^{1}H NMR and b) ^{13}C NMR of compd. **21b**

a)

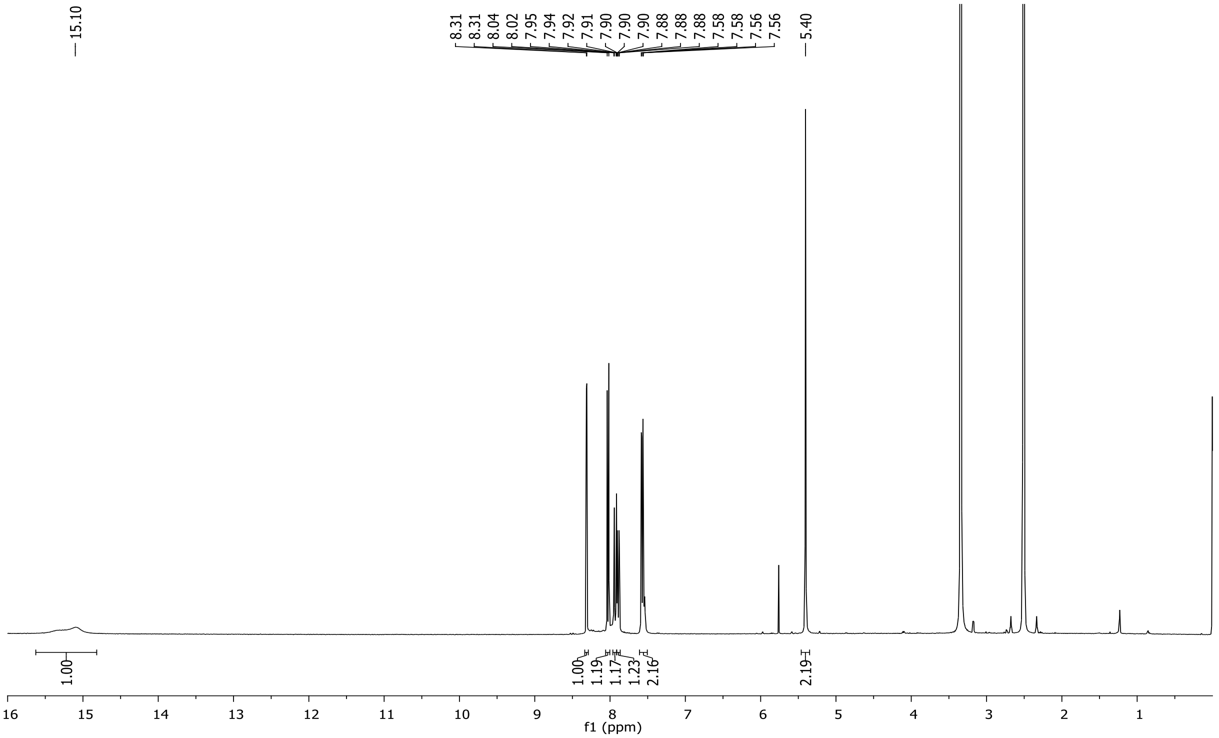

b)

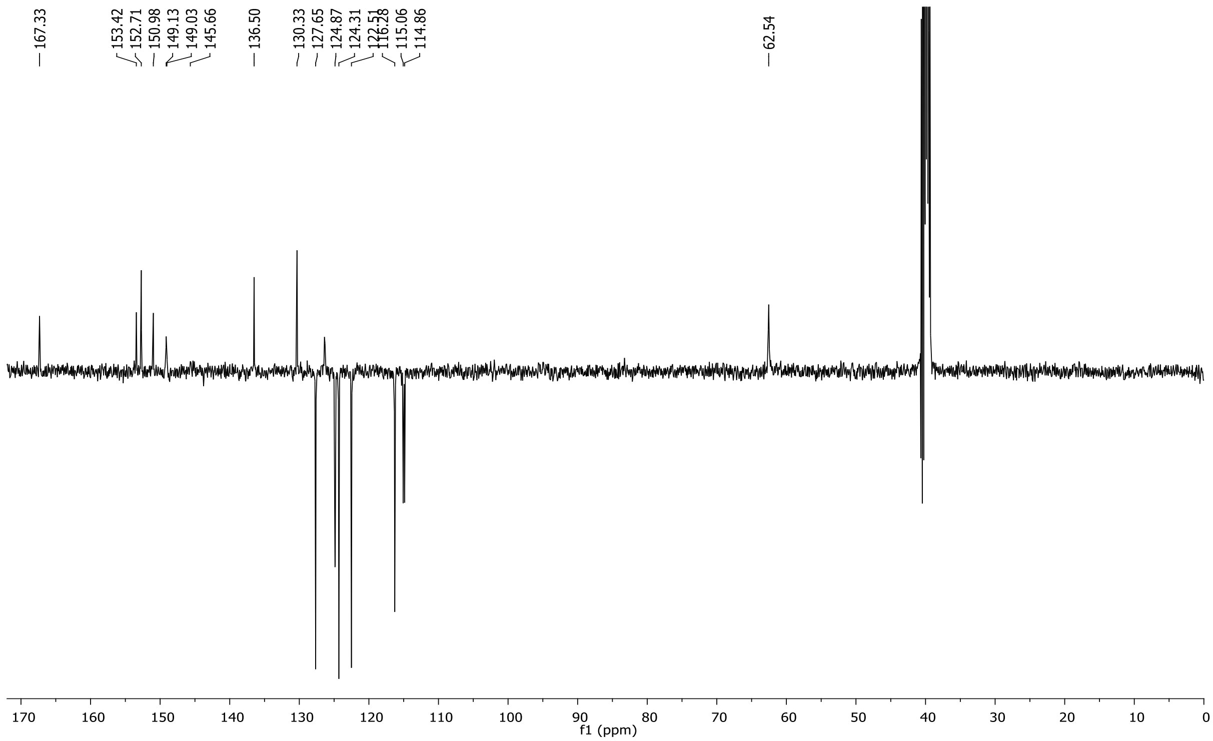

Fig. S39 a) ^{1}H NMR and b) ^{13}C NMR of compd. **21c**

a)

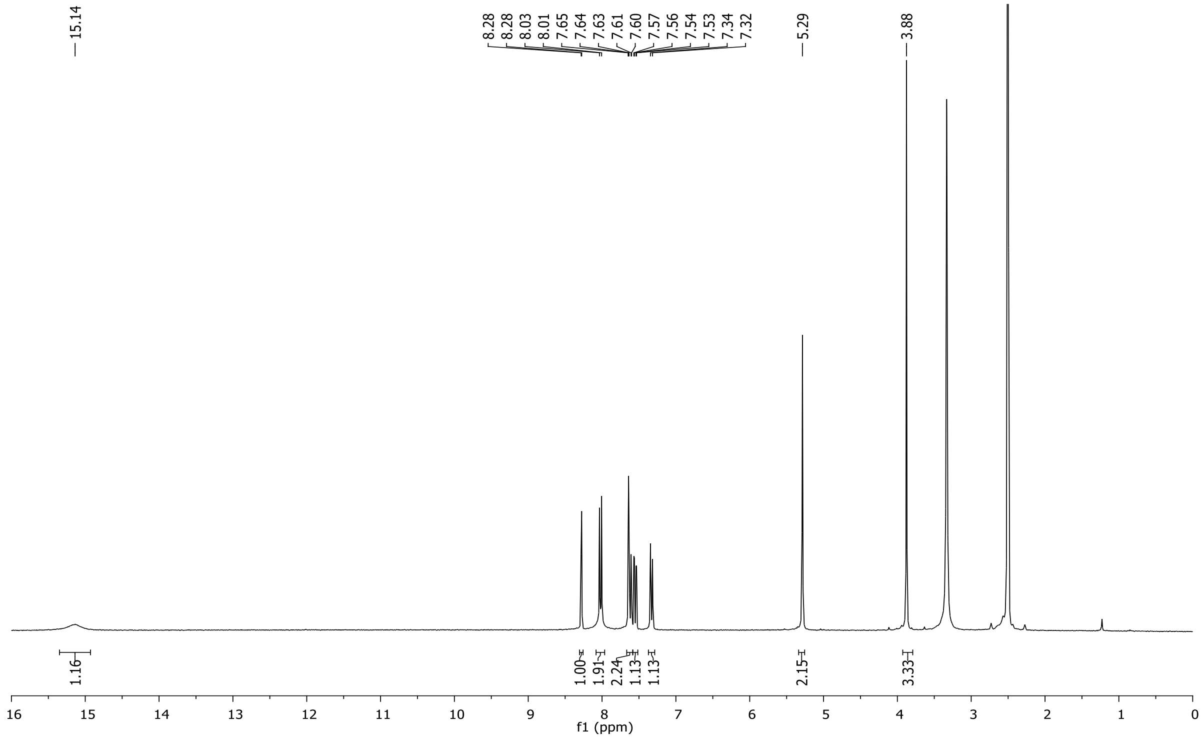

b)

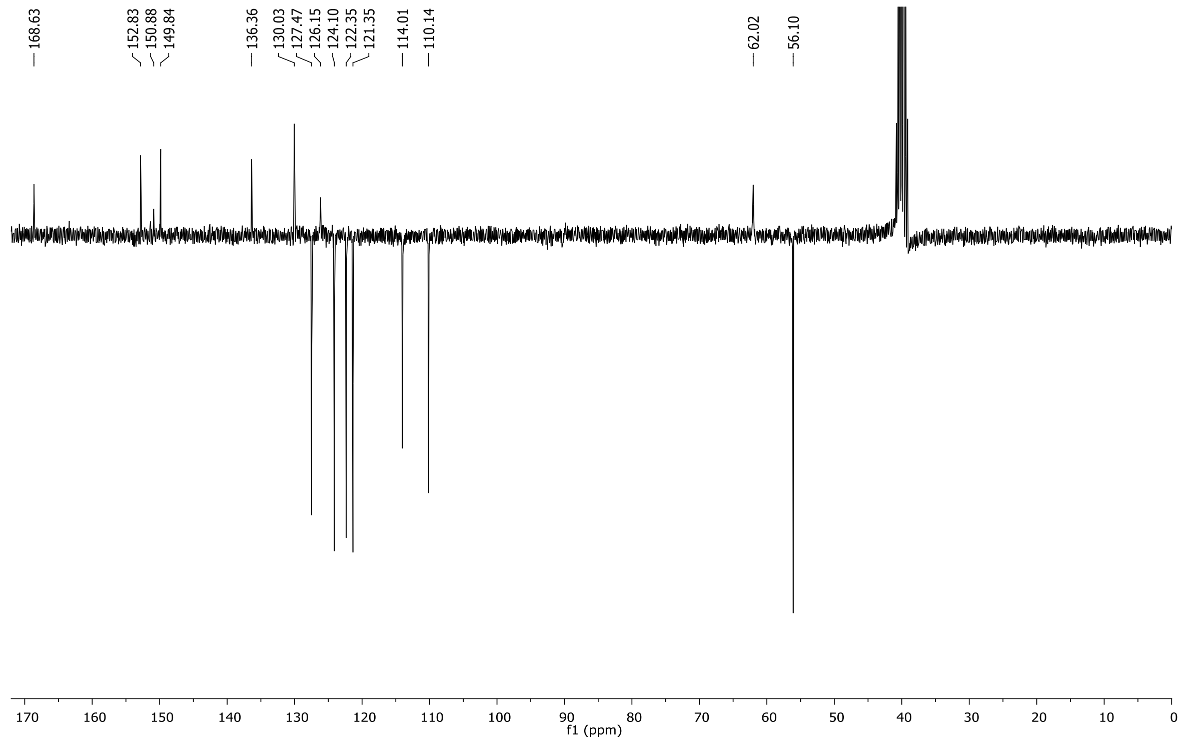

Fig. S40 a) ^{1}H NMR and b) ^{13}C NMR of compd. **22a**

a)

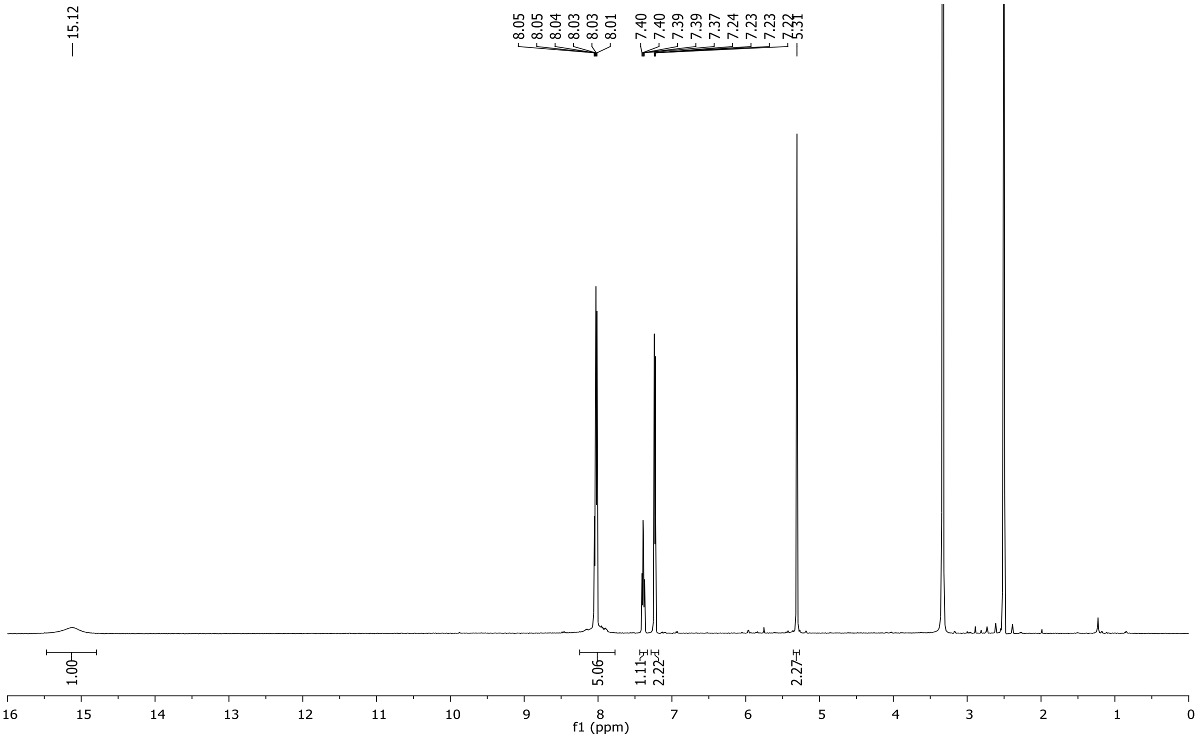

b)

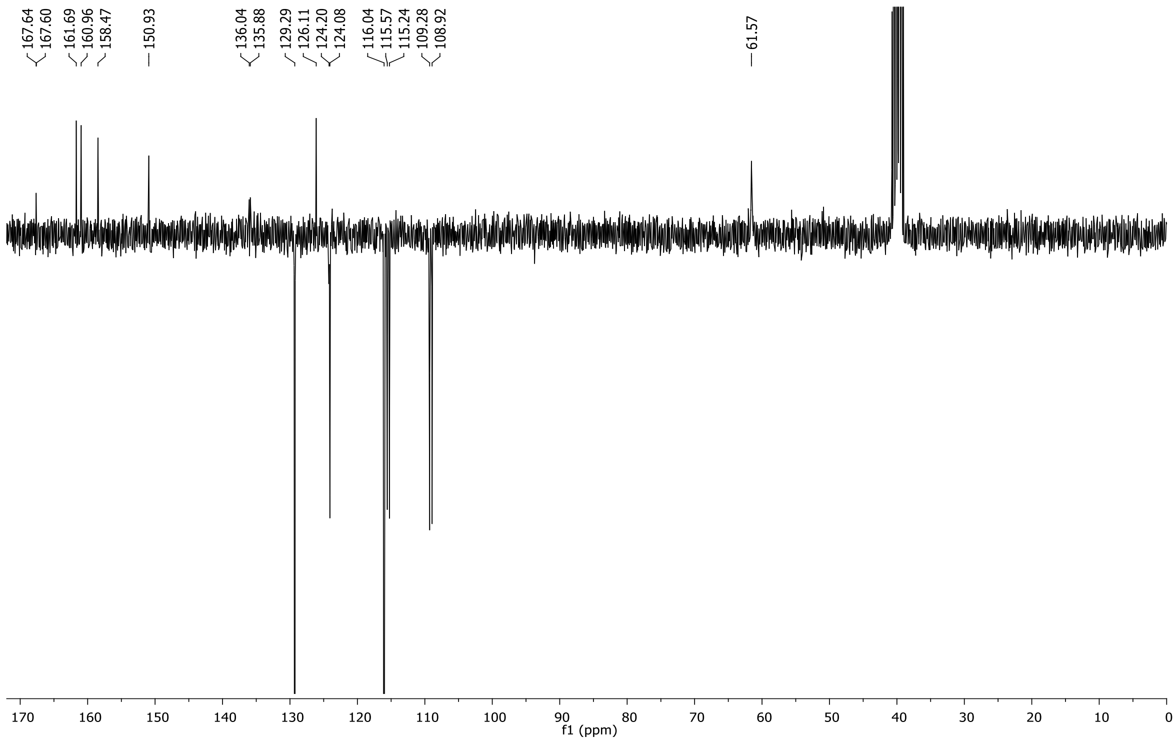

Fig. S41 a) ^{1}H NMR and b) ^{13}C NMR of compd. **22b**

a)

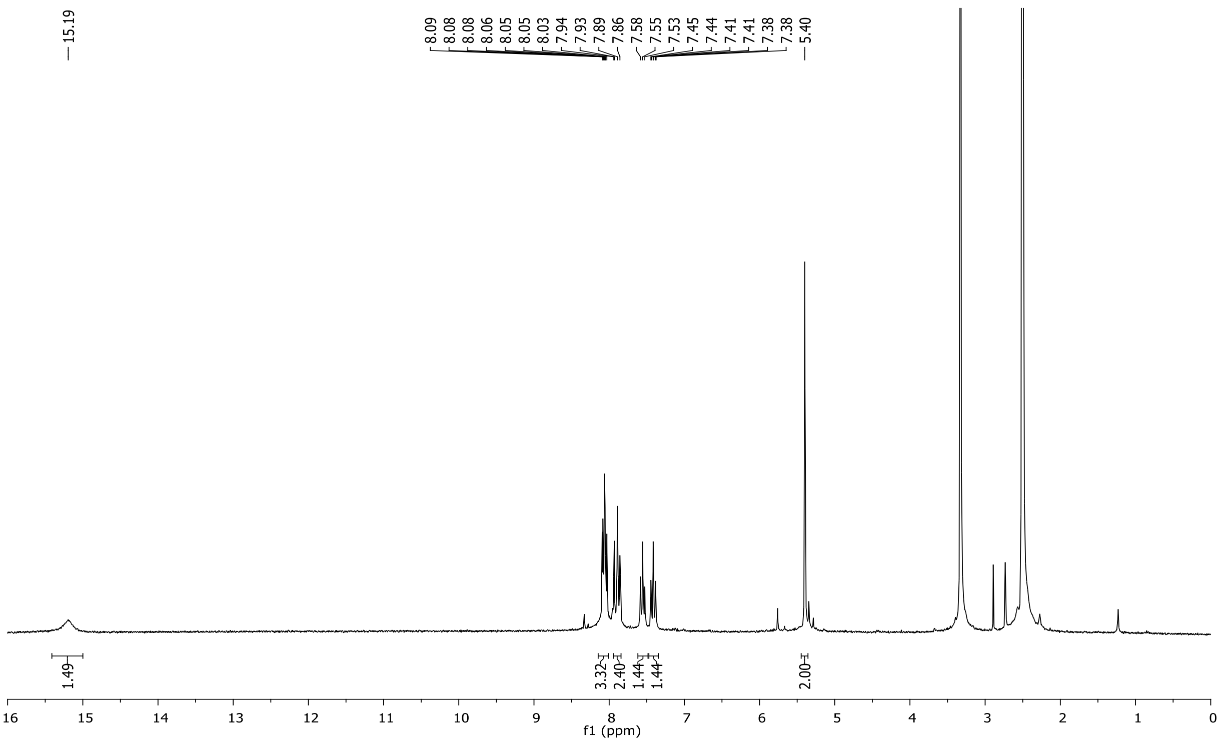

b)

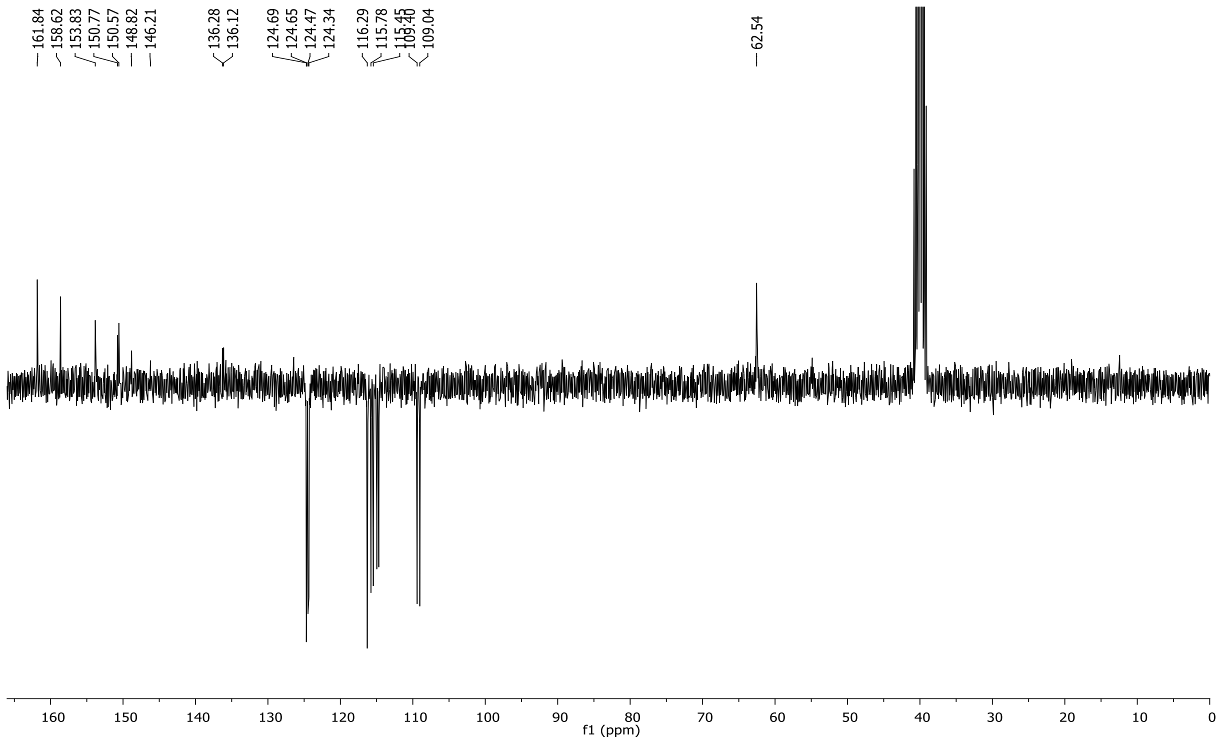

Fig. S42 a) ^{1}H NMR and b) ^{13}C NMR of compd. **22c**

a)

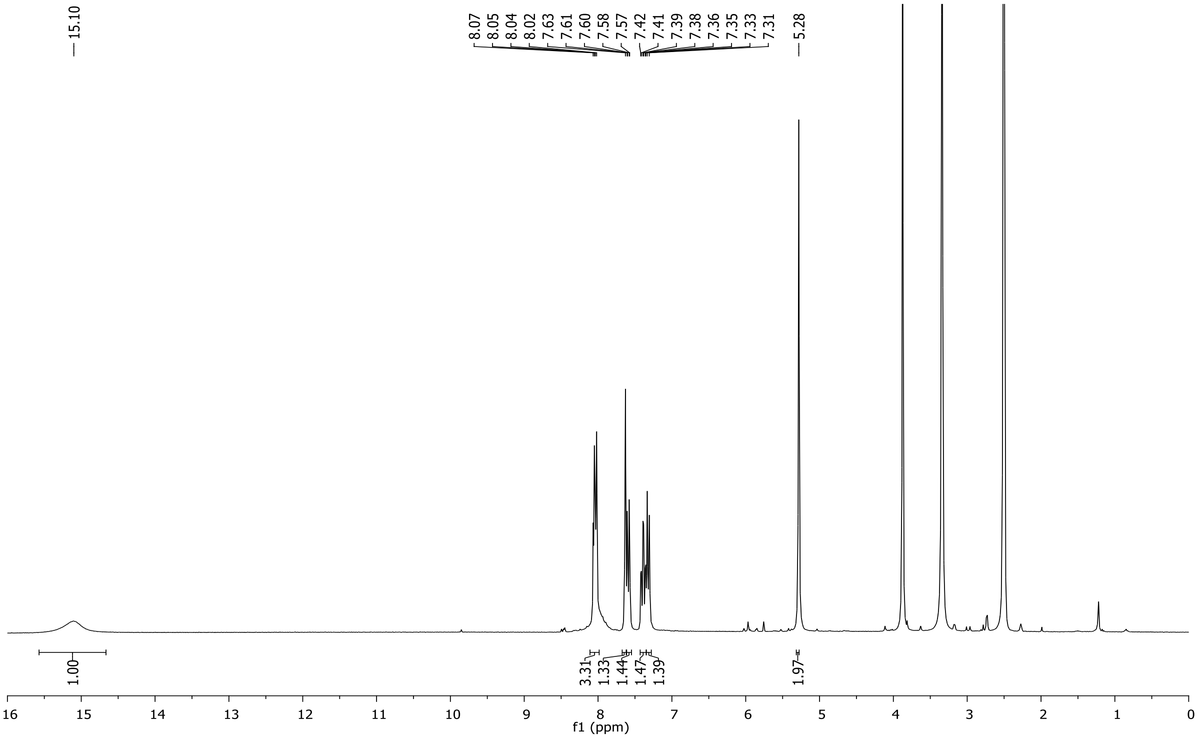

b)

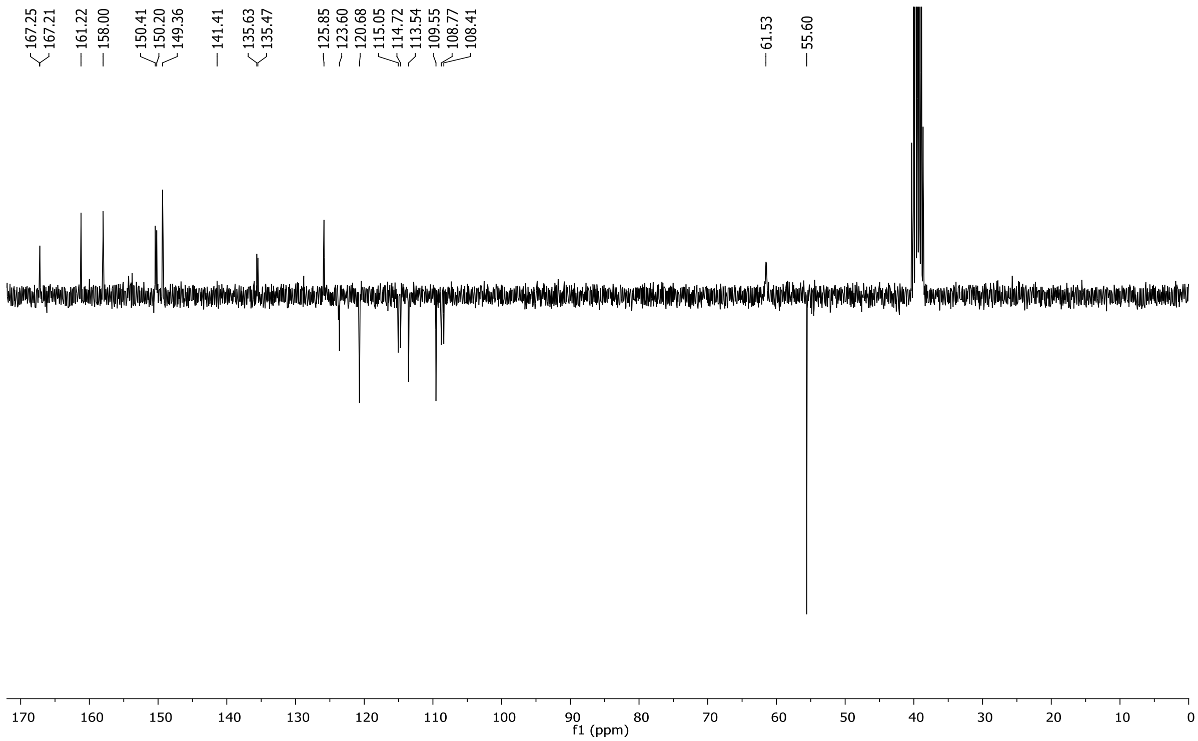

Fig. S43 a) ^{1}H NMR and b) ^{13}C NMR of compd. **23a**

a)

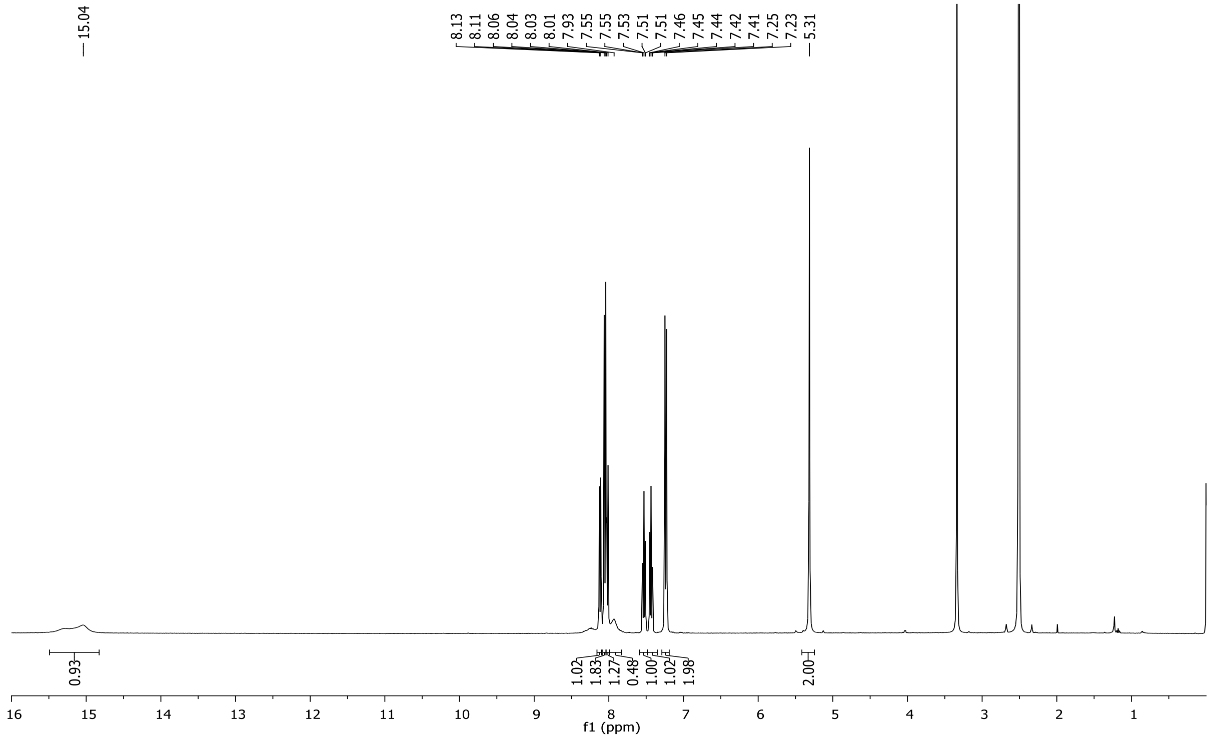

b)

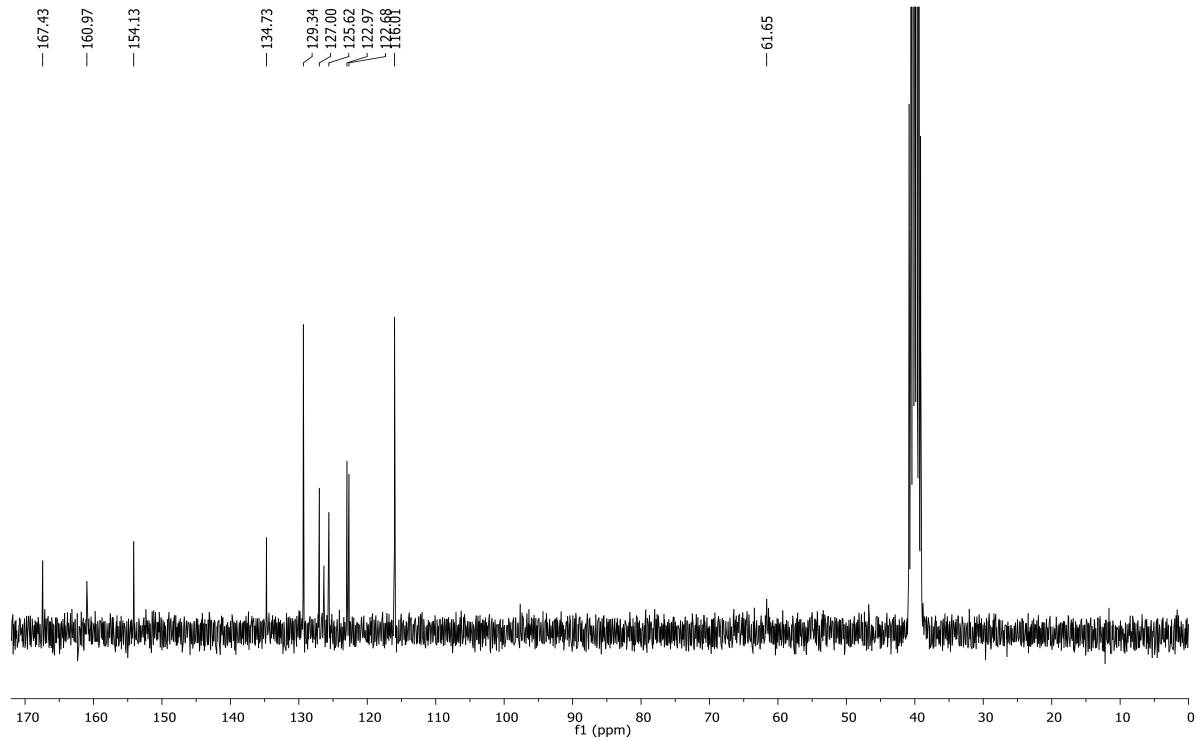

Fig. S44 a) ^{1}H NMR and b) ^{13}C NMR of compd. **23b**

a)

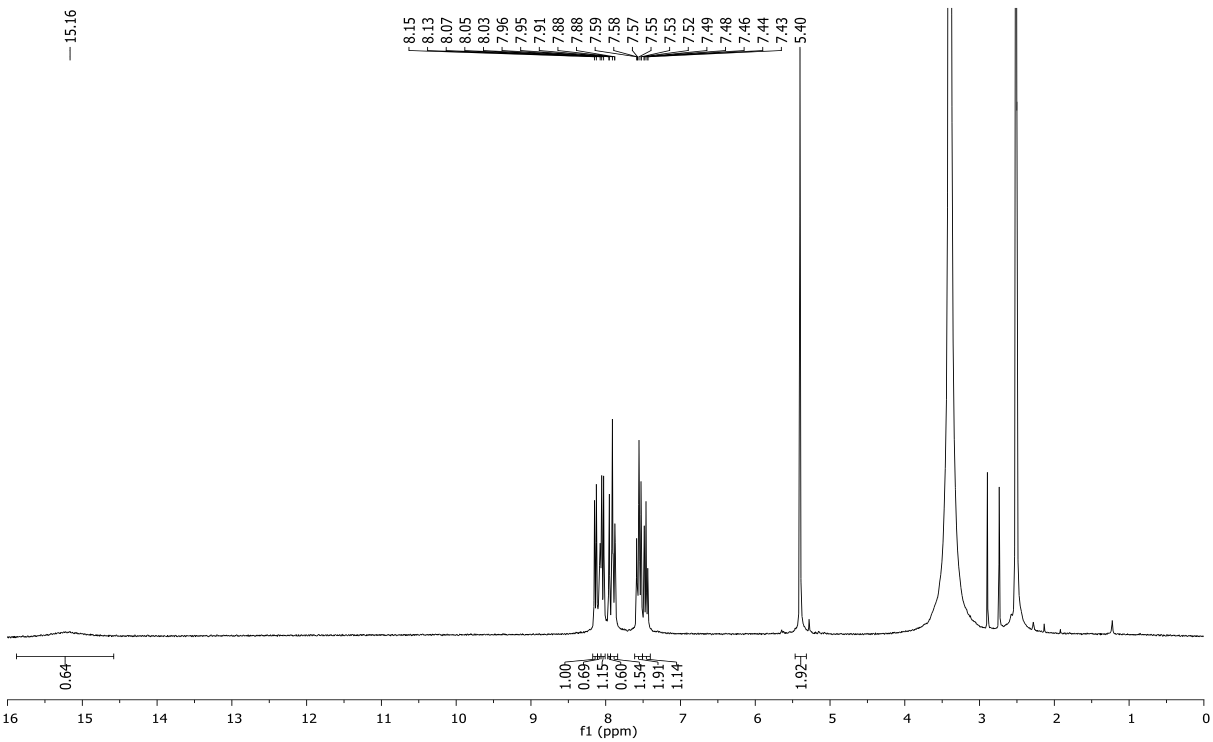

b)

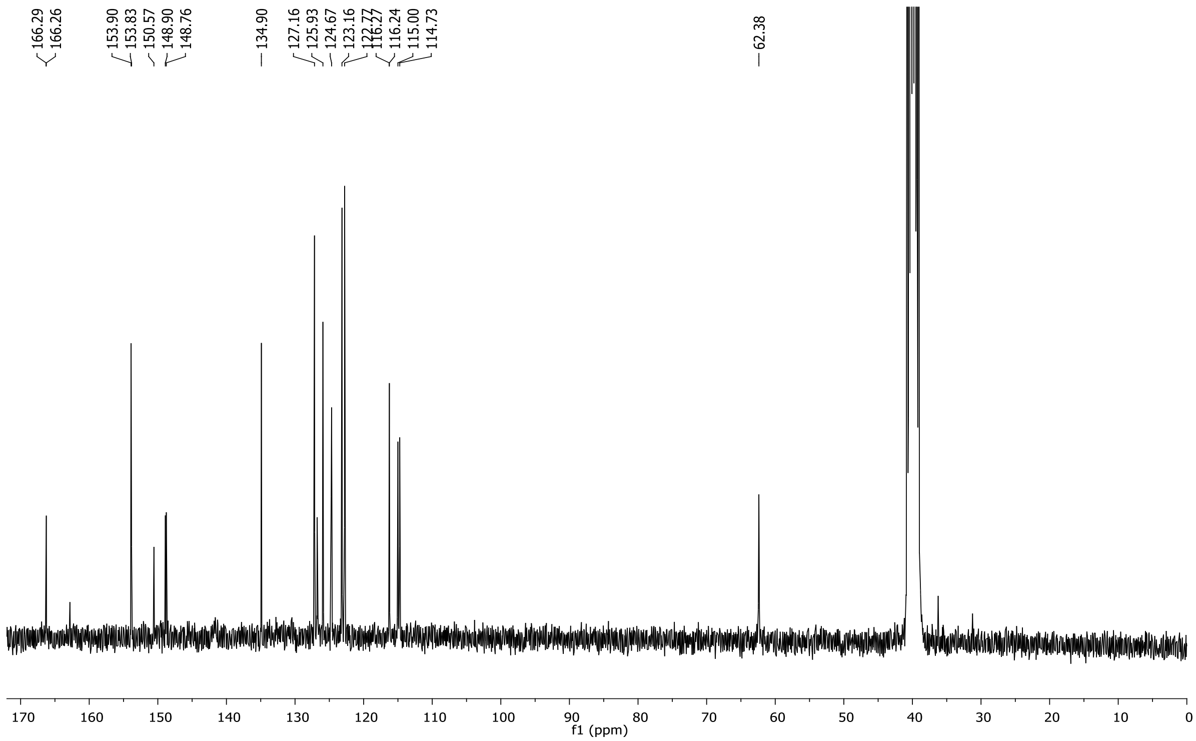

Fig. S45 a) ^{1}H NMR and b) ^{13}C NMR of compd. **23c**

a)

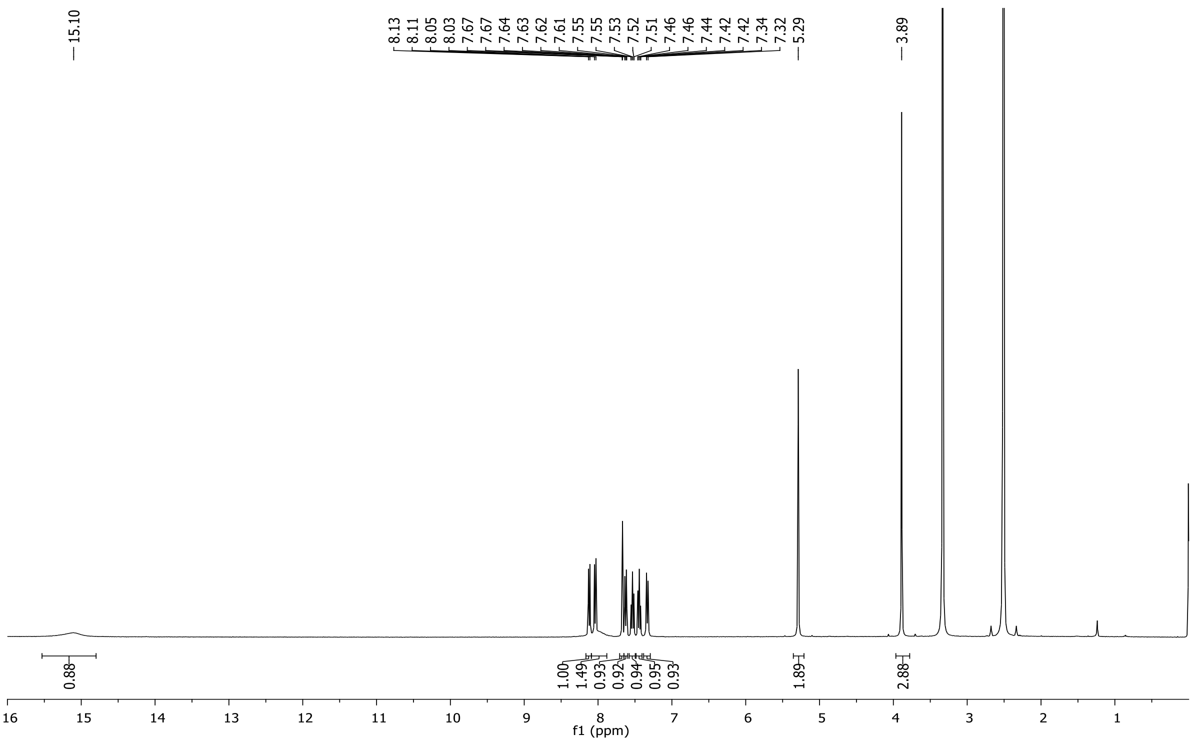

b)

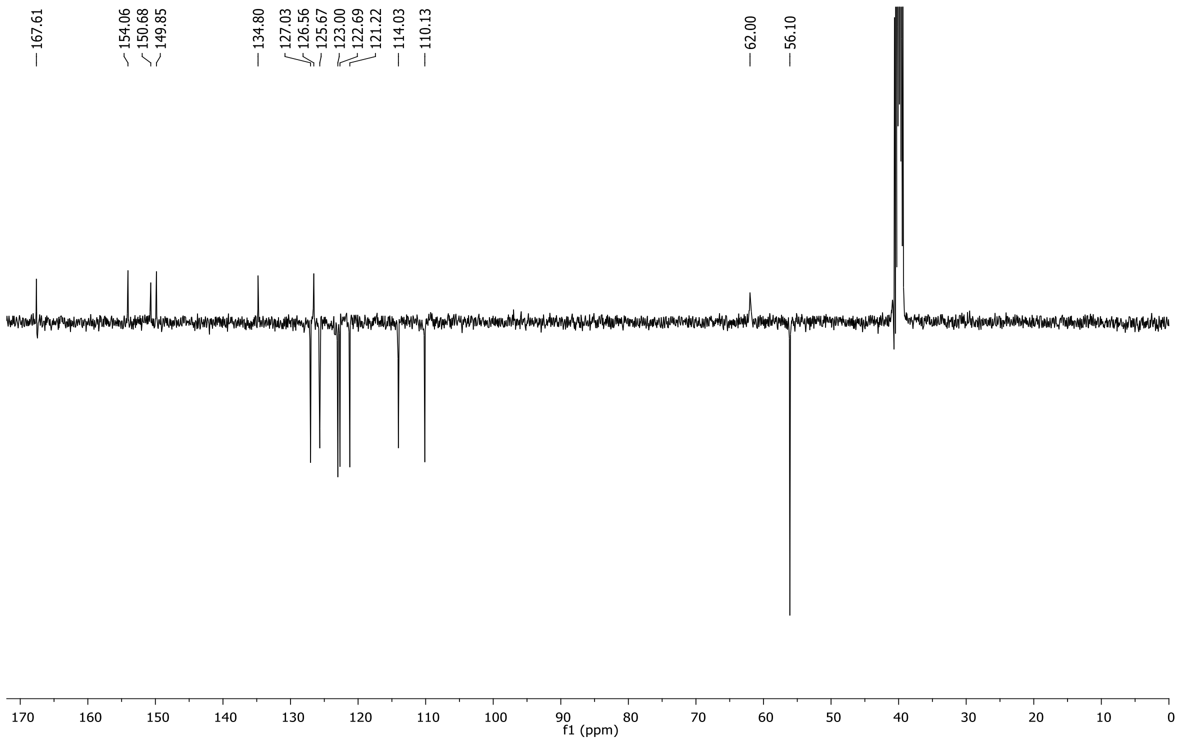

Fig. S46 a) ^{1}H NMR and b) ^{13}C NMR of compd. **24a**

a)

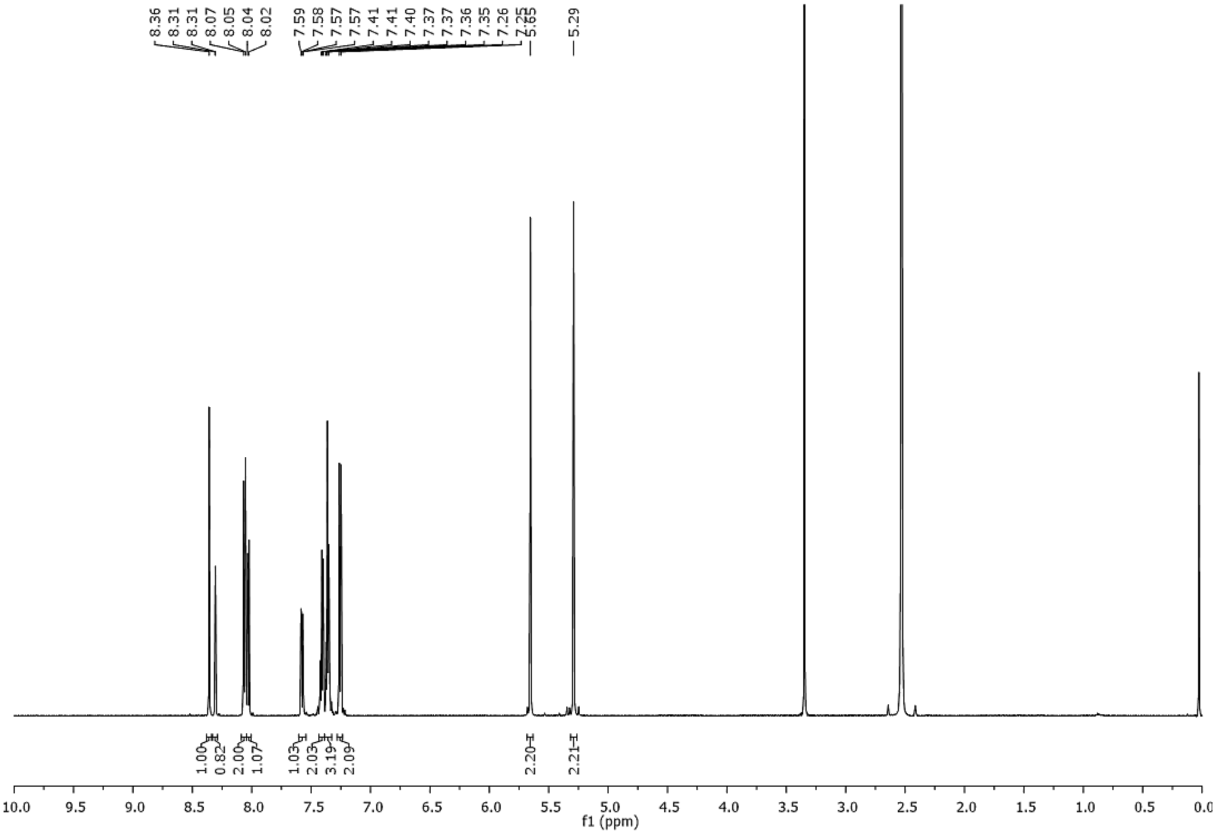

b)

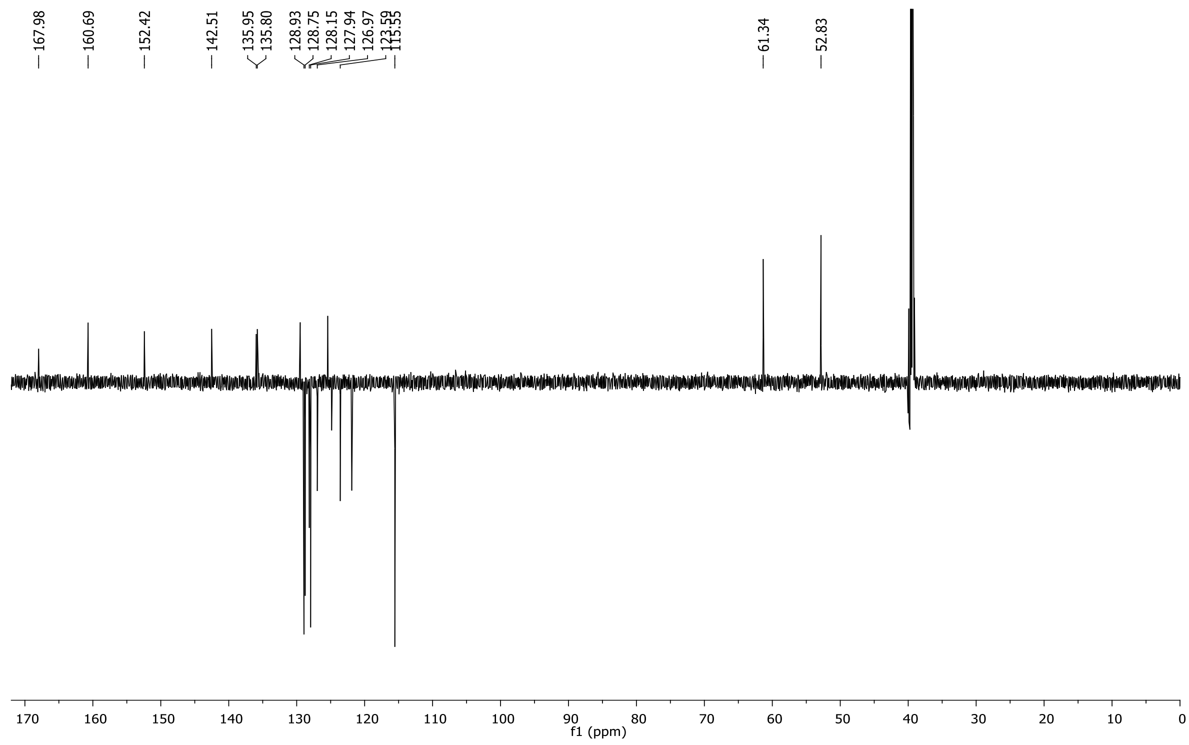

Fig. S47 a) ^{1}H NMR and b) ^{13}C NMR of compd. **24b**

a)

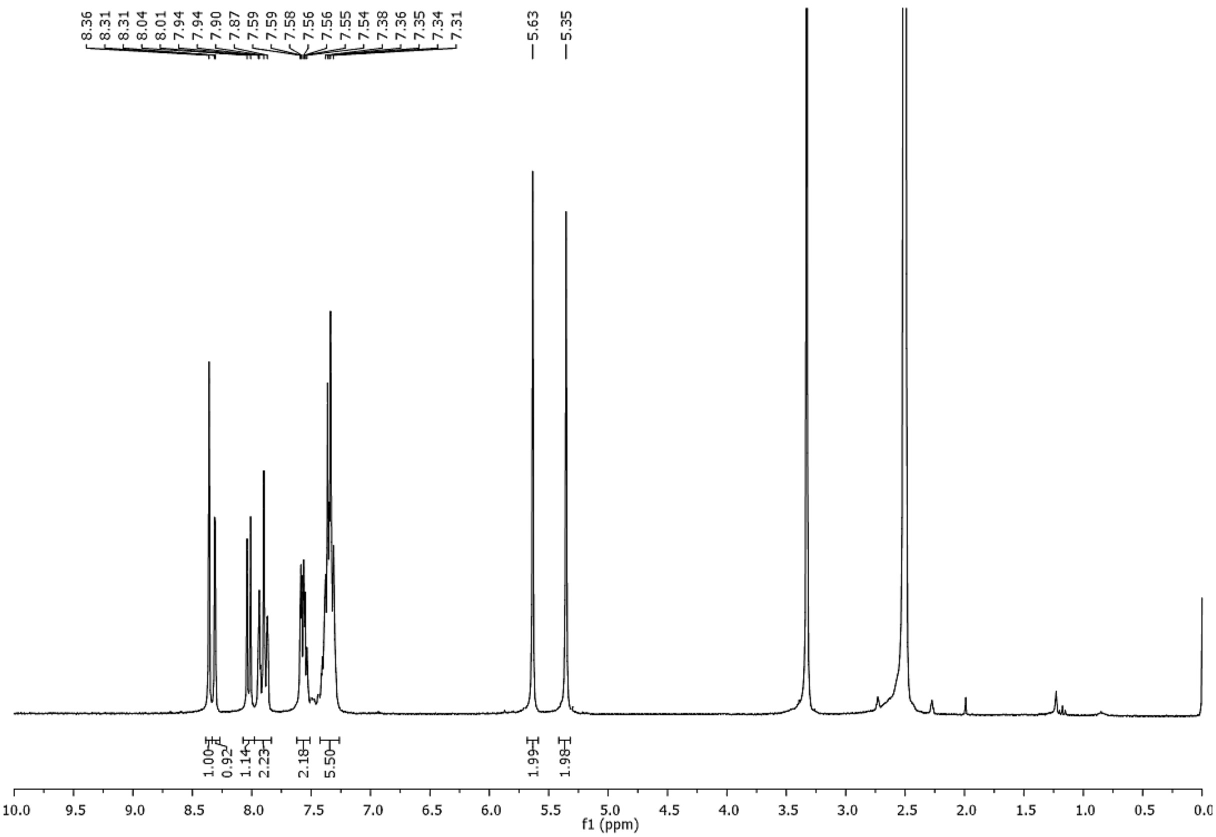

b)

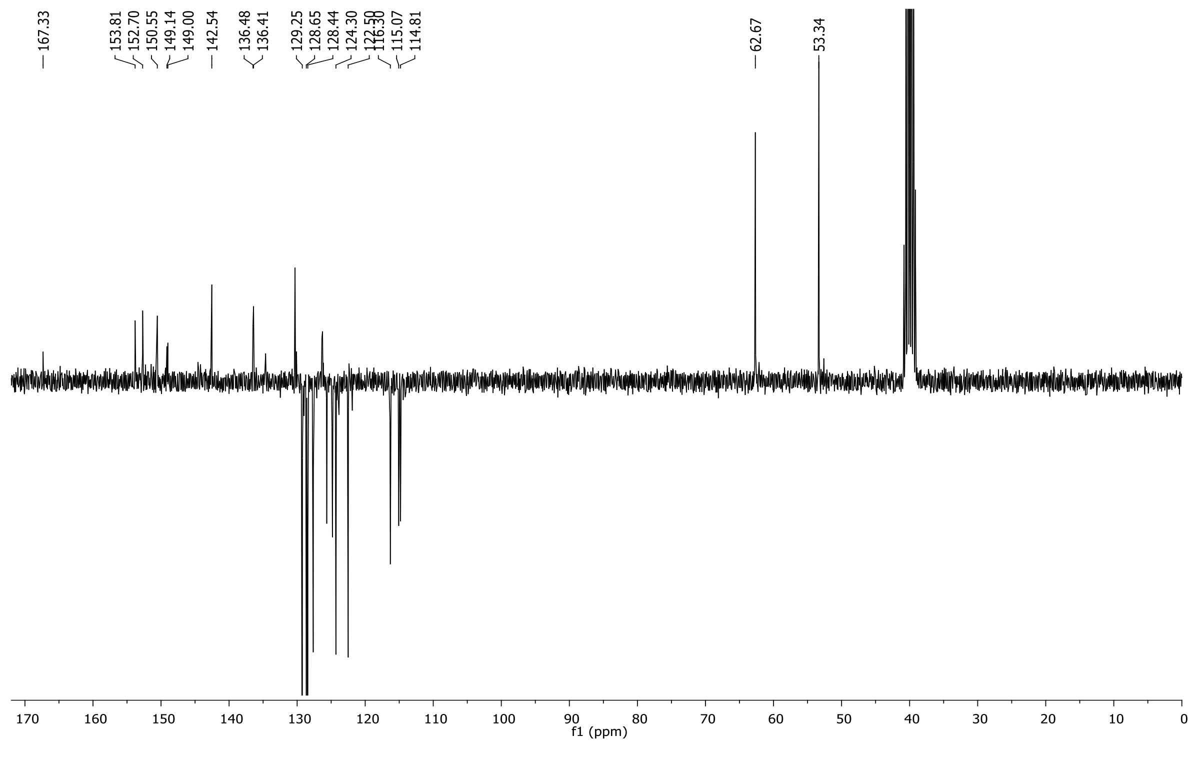

Fig. S48 a) ^{1}H NMR and b) ^{13}C NMR of compd. **24c**

a)

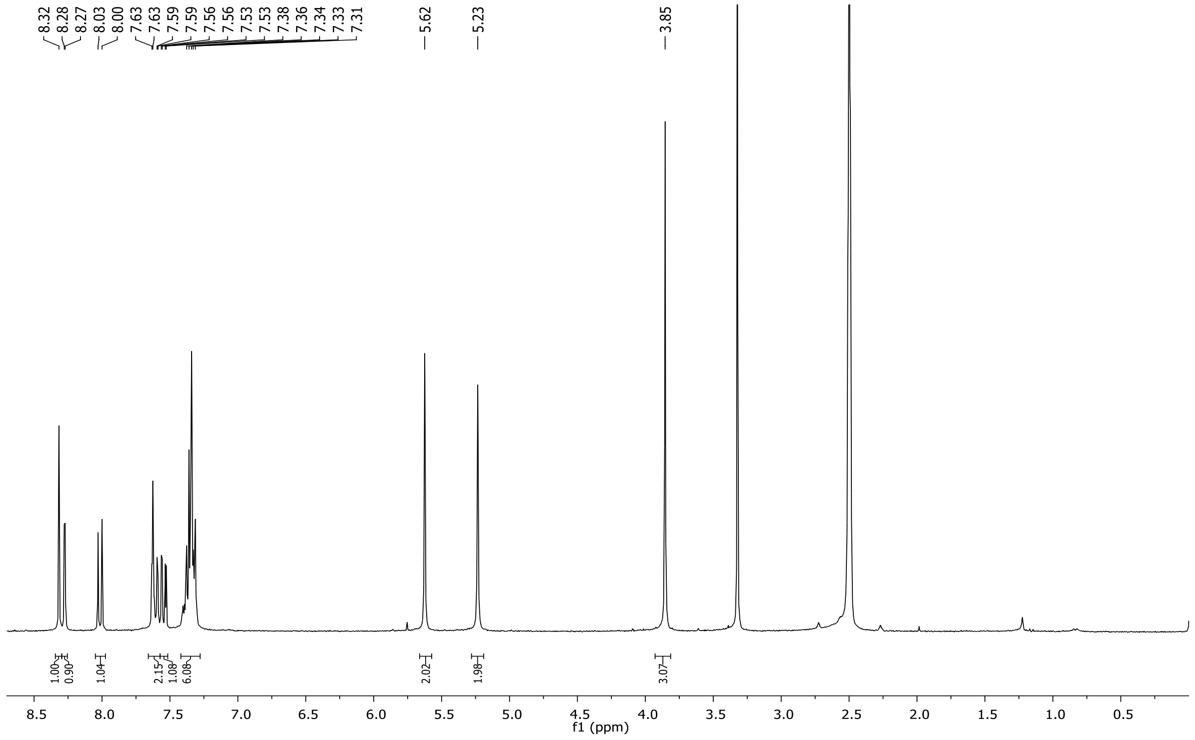

b)

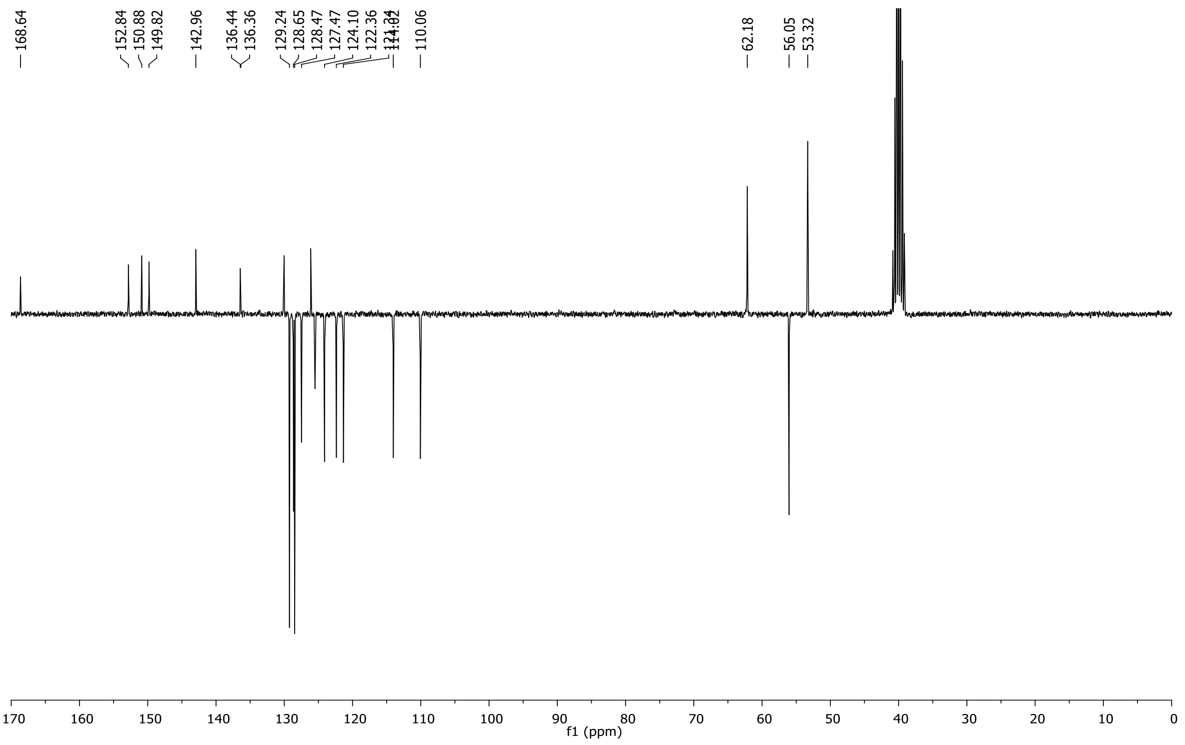

Fig. S49 a) ^{1}H NMR and b) ^{13}C NMR of compd. **25a**

a)

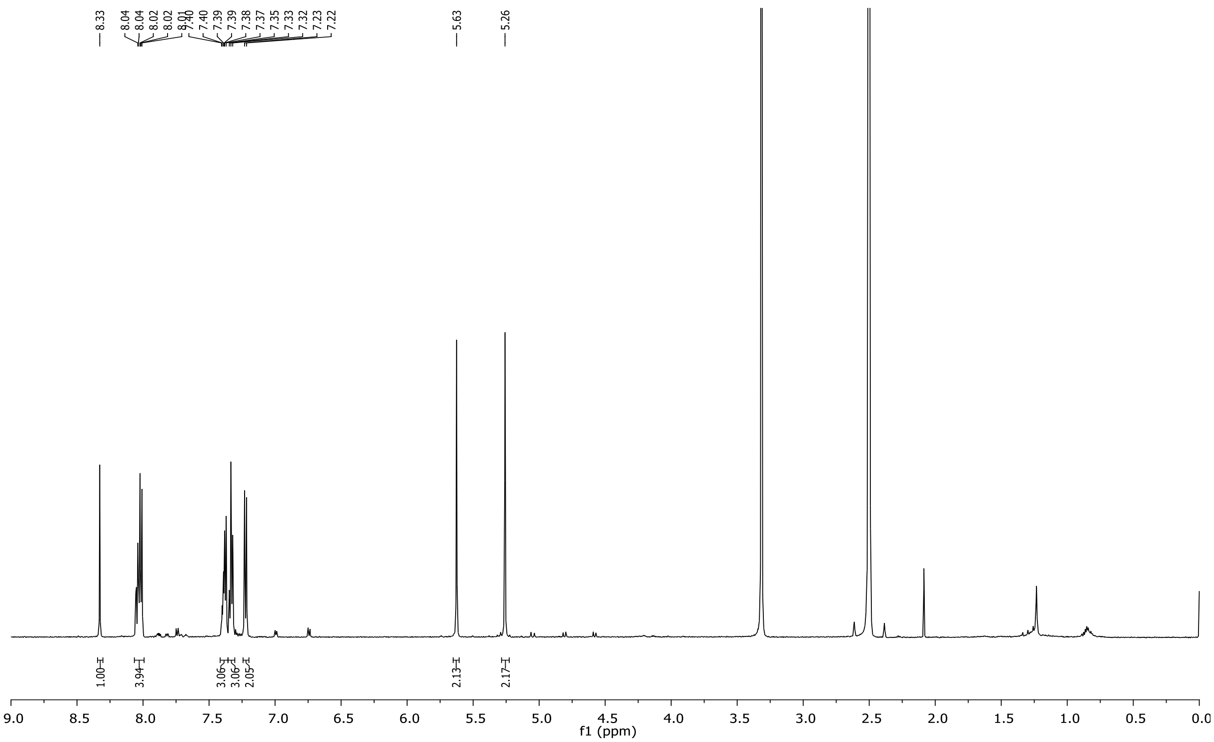

b)

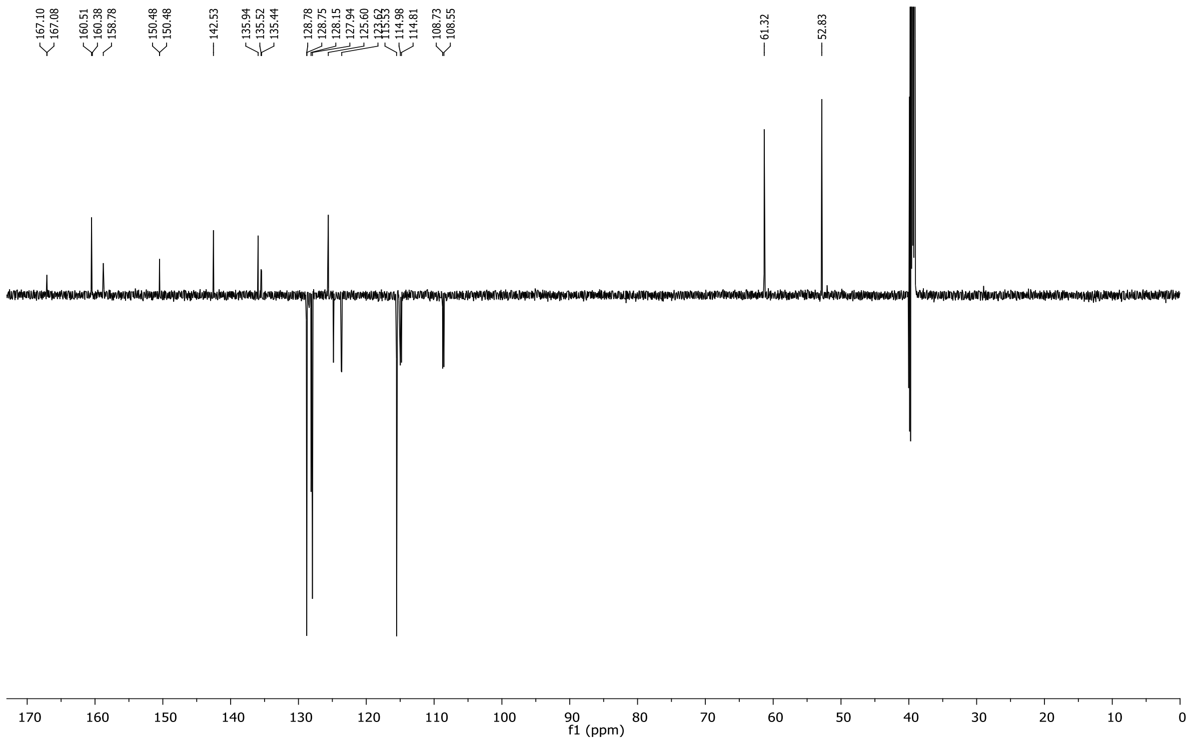

Fig. S50 a) ^{1}H NMR and b) ^{13}C NMR of compd. **25b**

a)

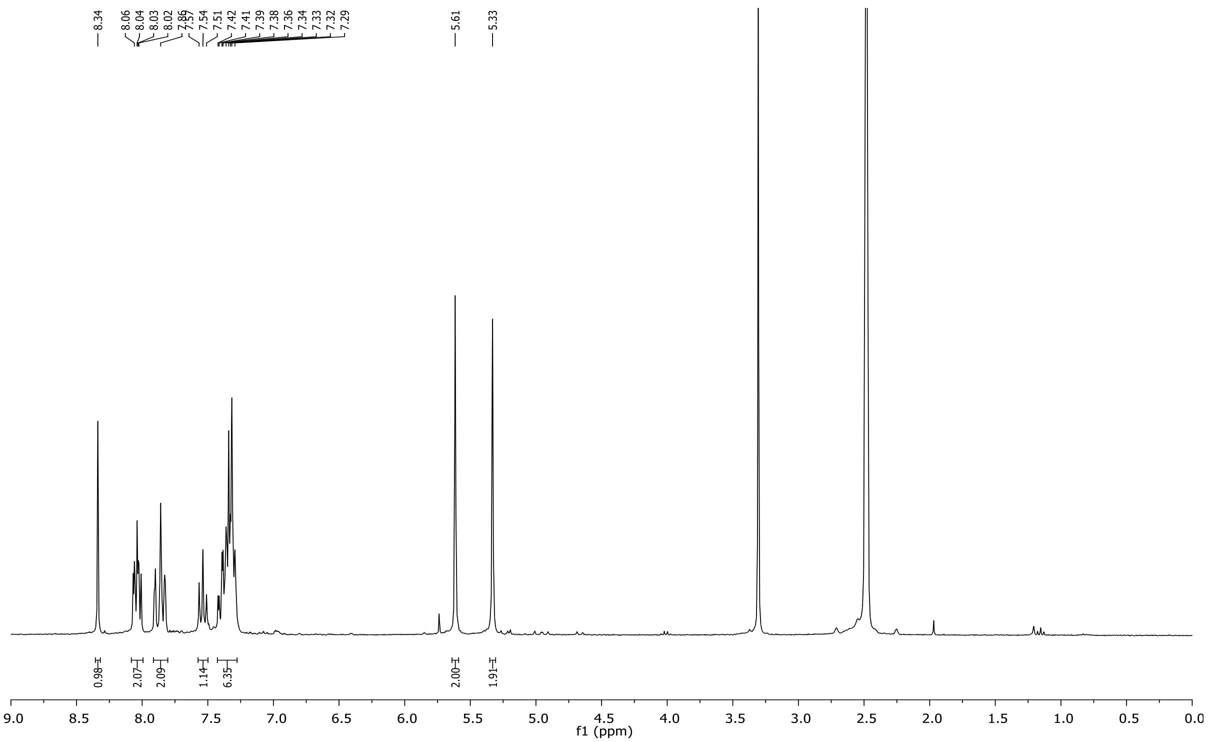

b)

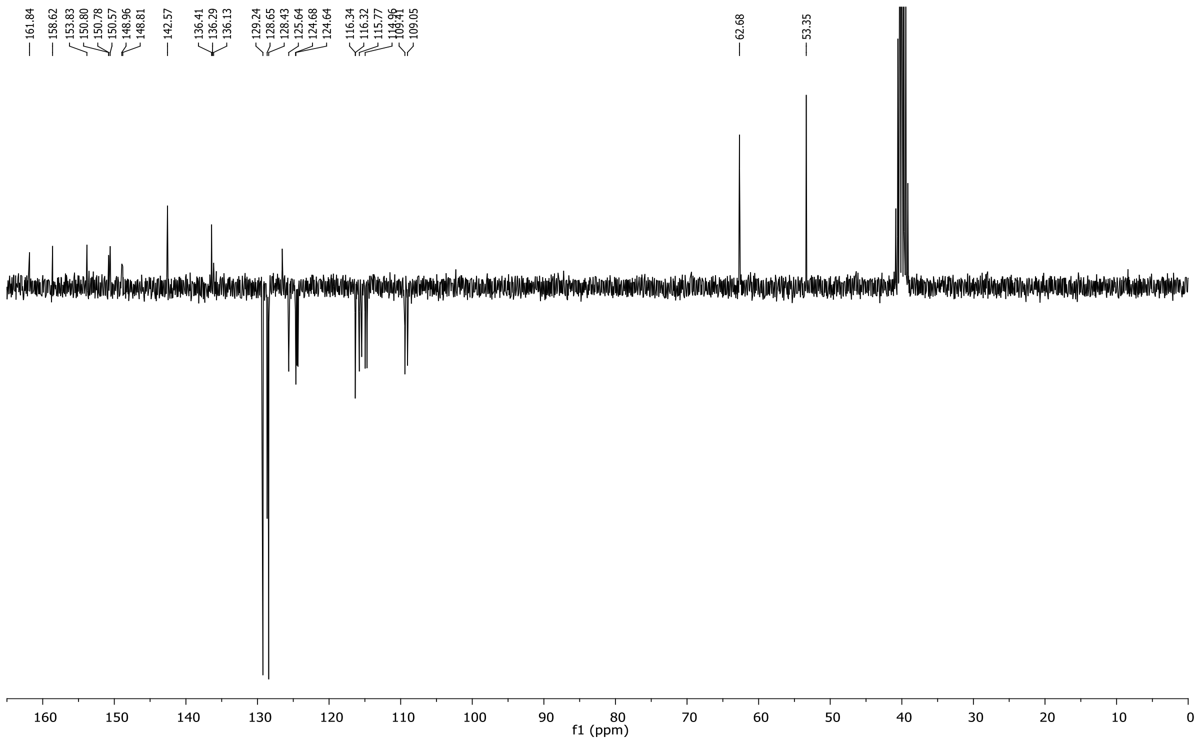

Fig. S51 a) ^{1}H NMR and b) ^{13}C NMR of compd. **25c**

a)

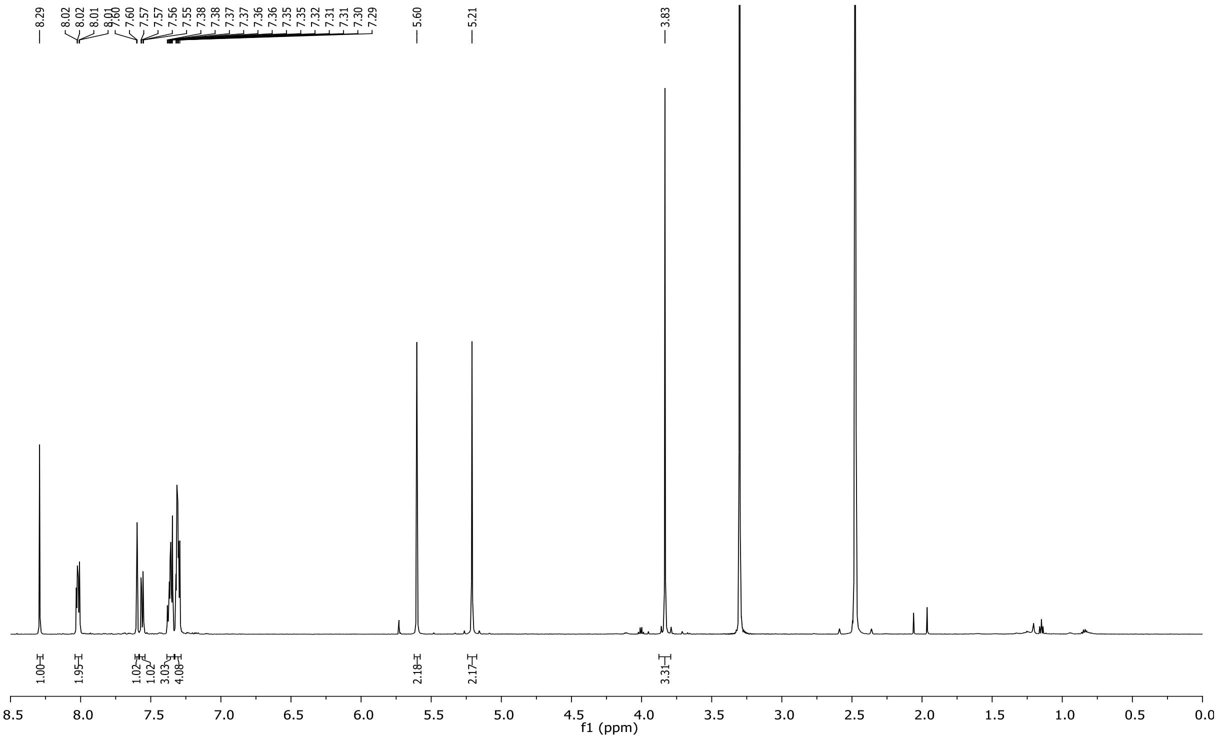

b)

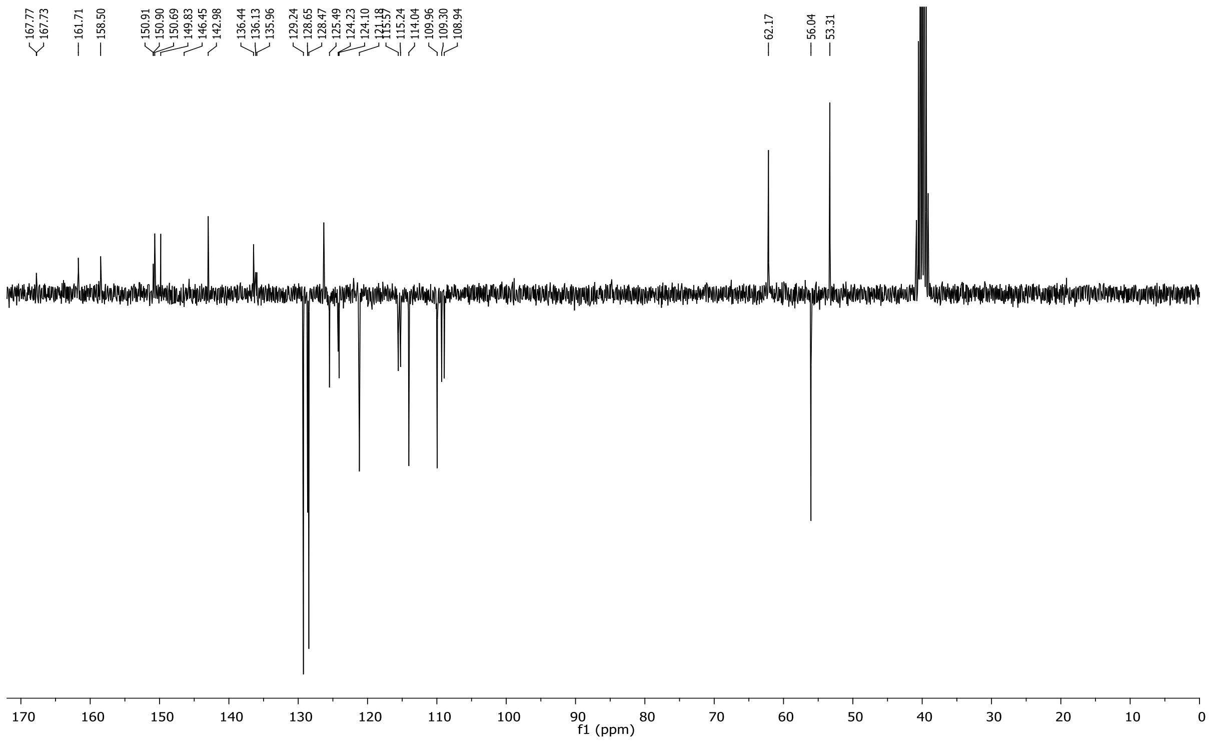

Fig. S52 a) ^{1}H NMR and b) ^{13}C NMR of compd. **26a**

a)

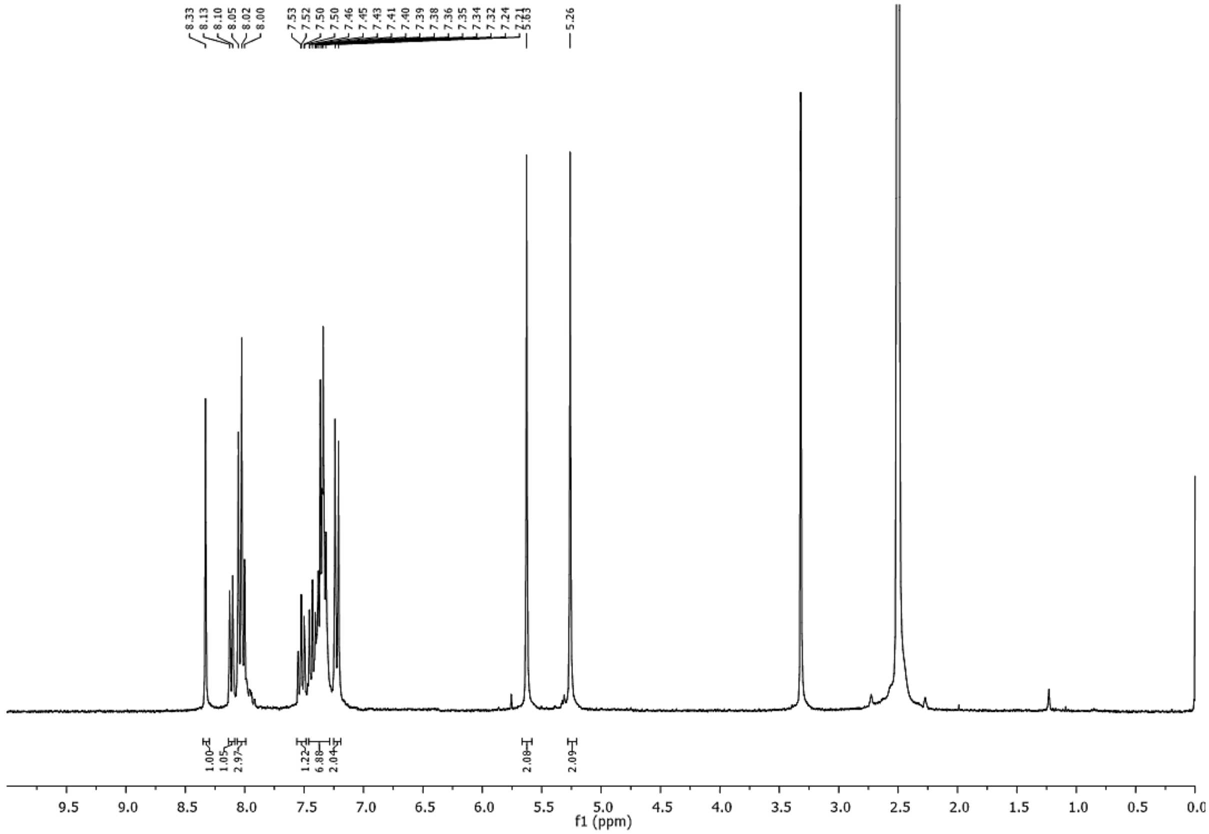

b)

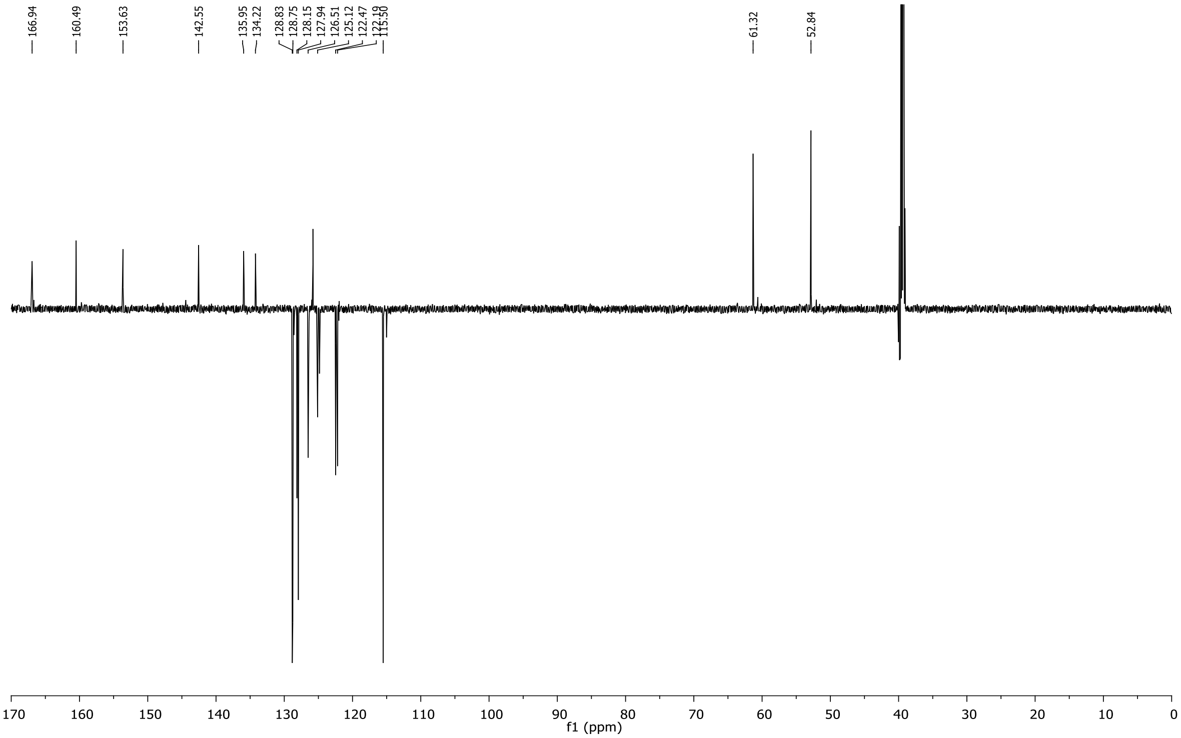

Fig. S53 a) ^{1}H NMR and b) ^{13}C NMR of compd. **26b**

a)

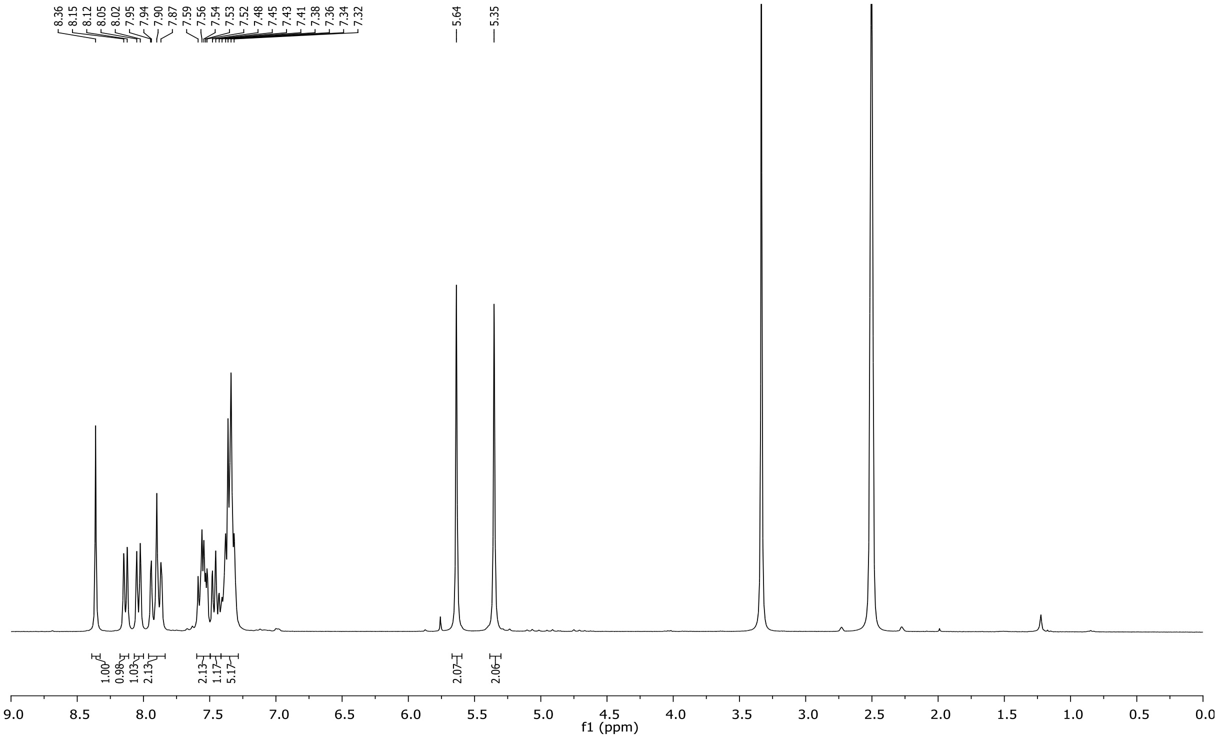

b)

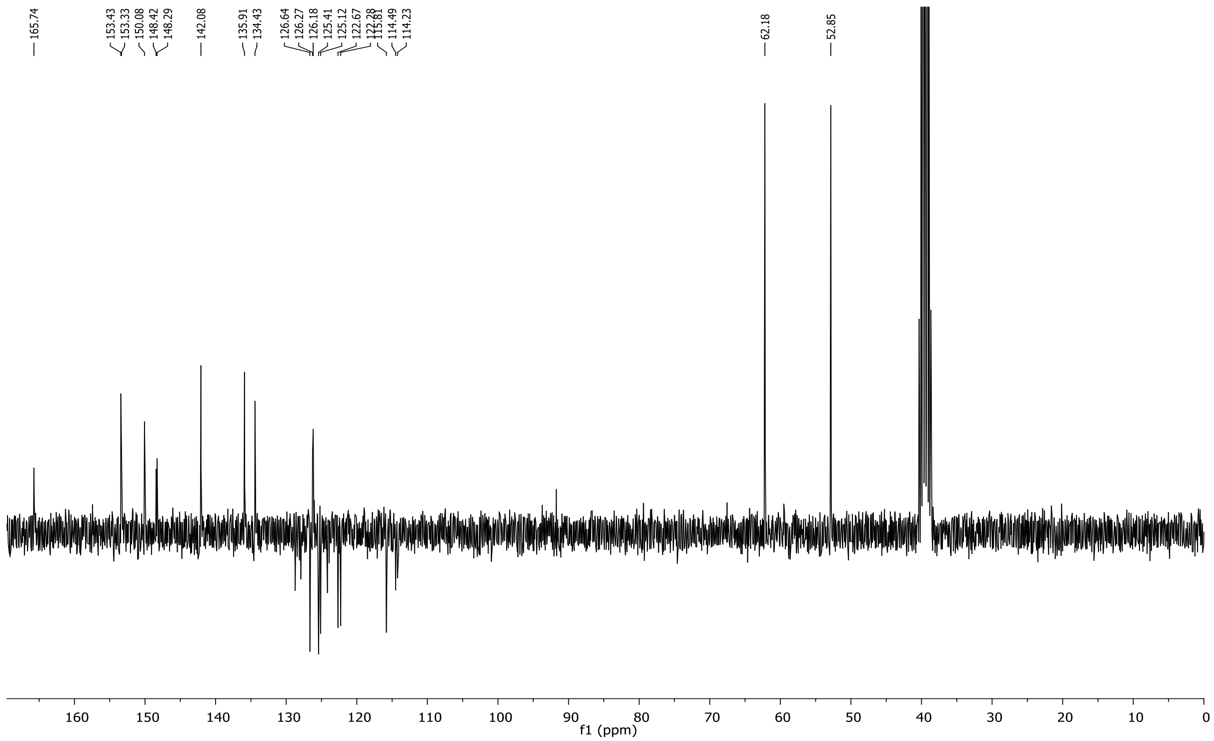

Fig. S54 a) ^{1}H NMR and b) ^{13}C NMR of compd. **26c**

a)

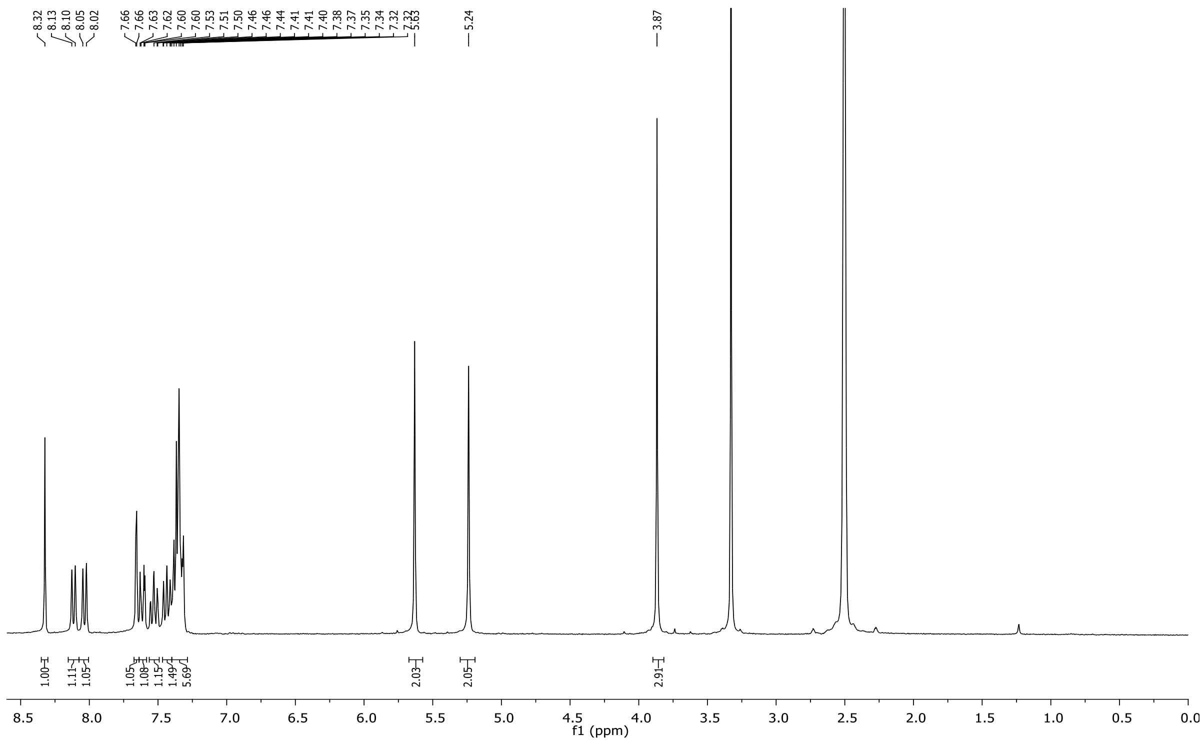

b)

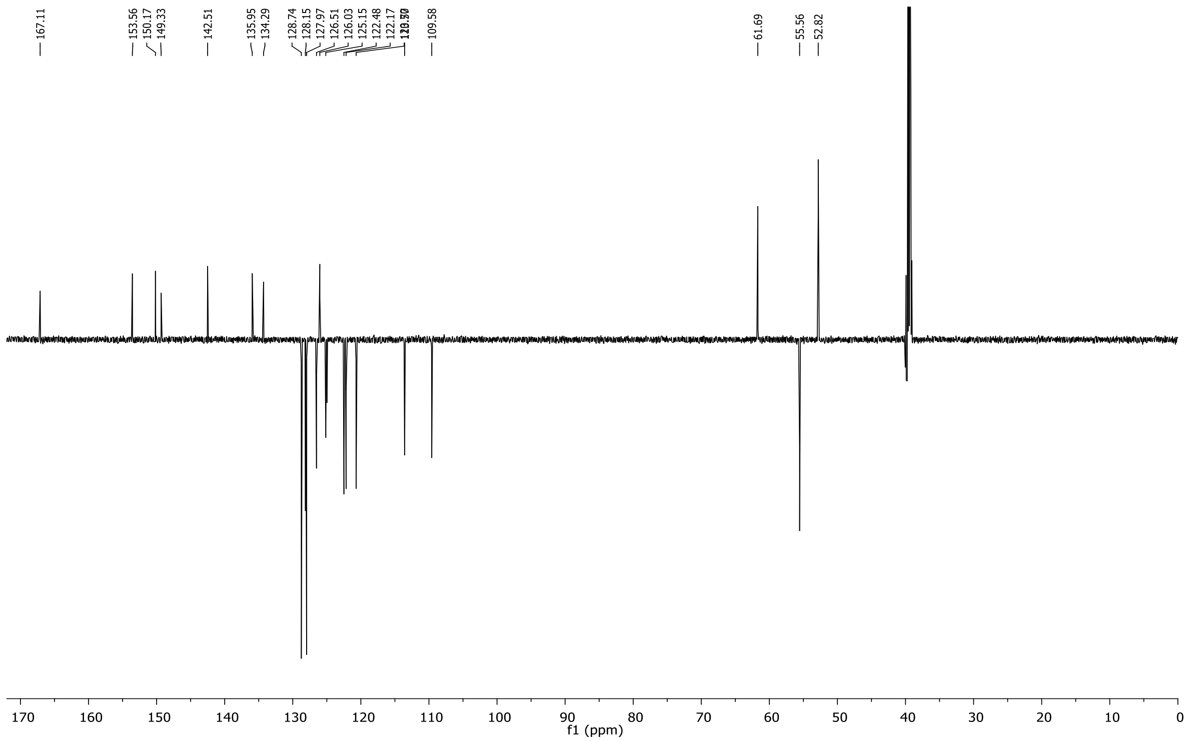

Fig. S55 a) ^{1}H NMR and b) ^{13}C NMR of compd. **34a**

a)

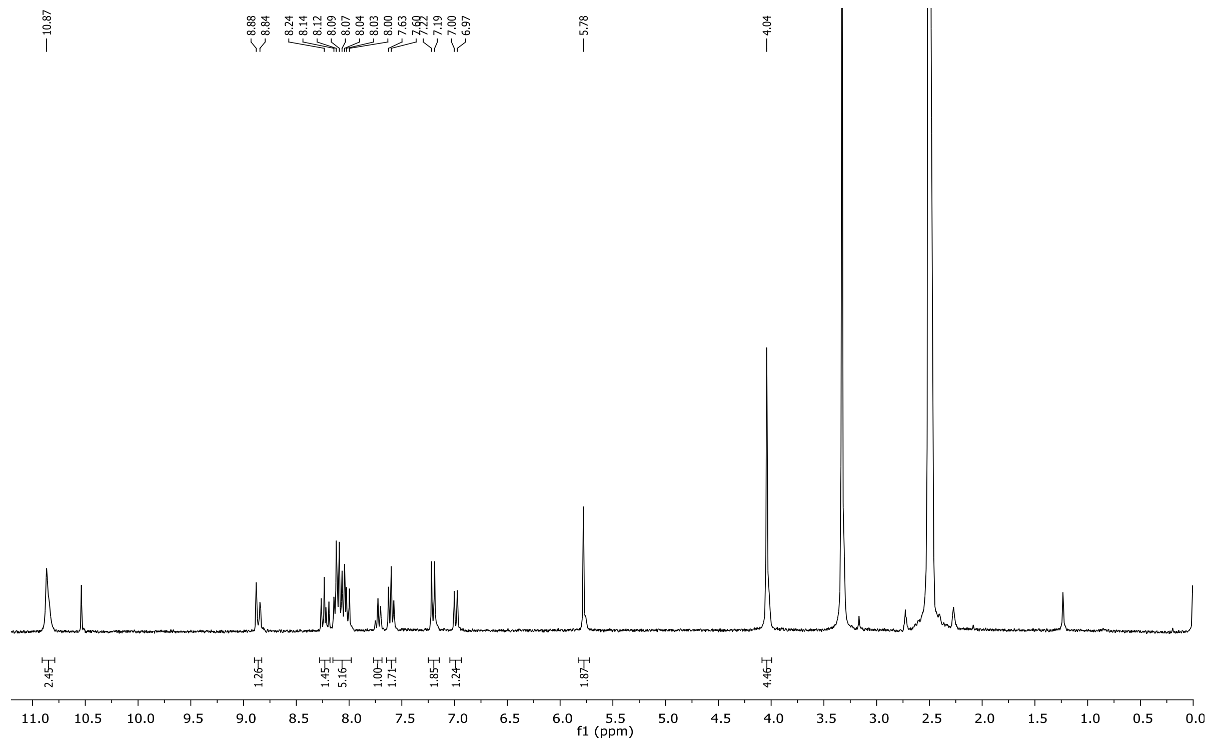

b)

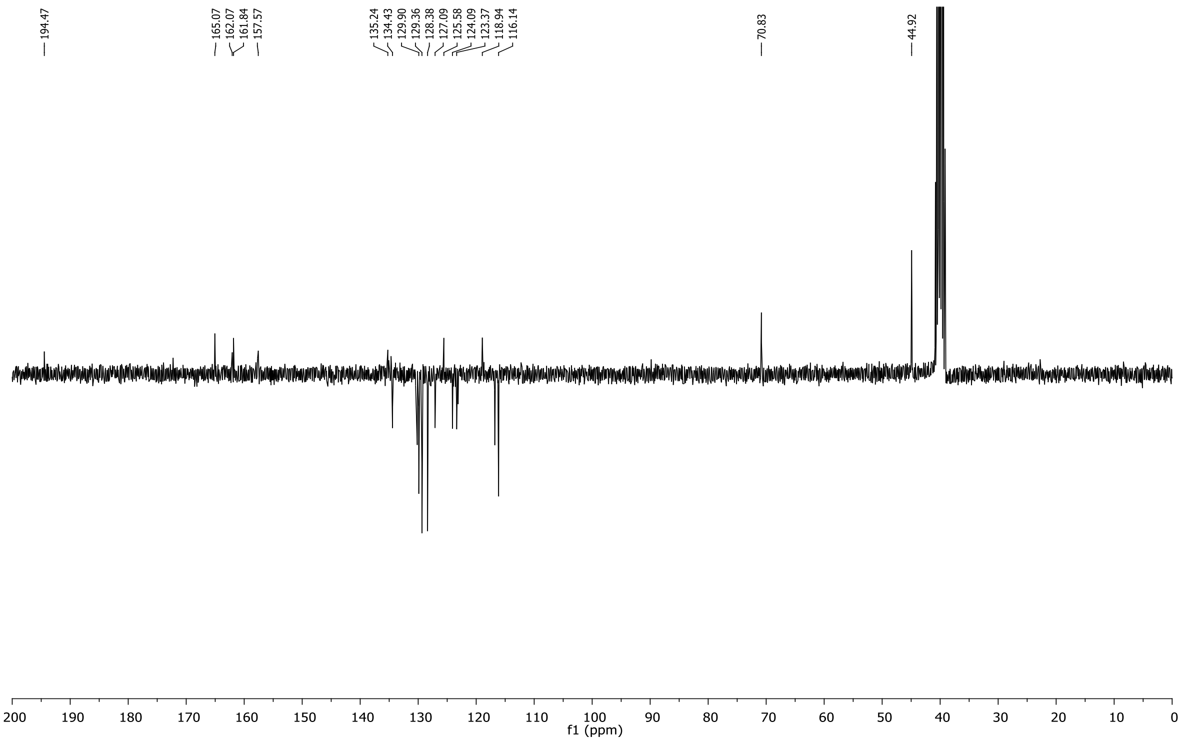

Fig. S56 a) ^{1}H NMR and b) ^{13}C NMR of compd. **34b**

a)

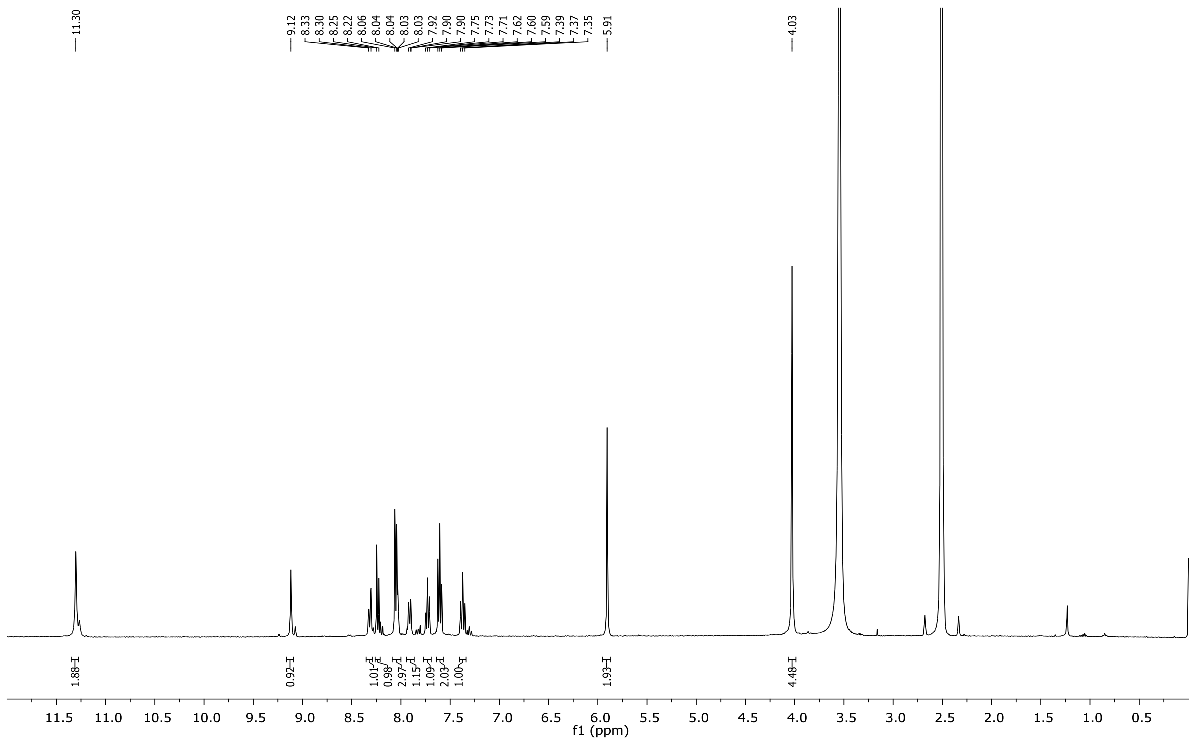

b)

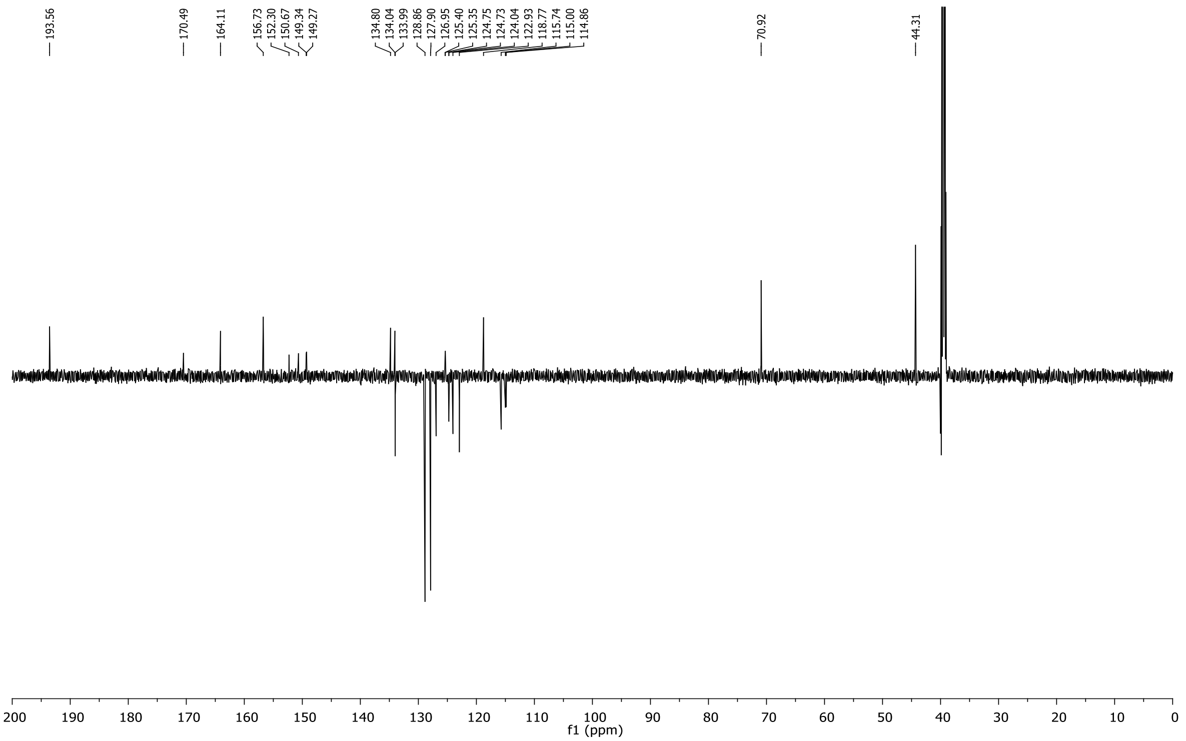

Fig. S57 a) ^{1}H NMR and b) ^{13}C NMR of compd. **34c**

a)

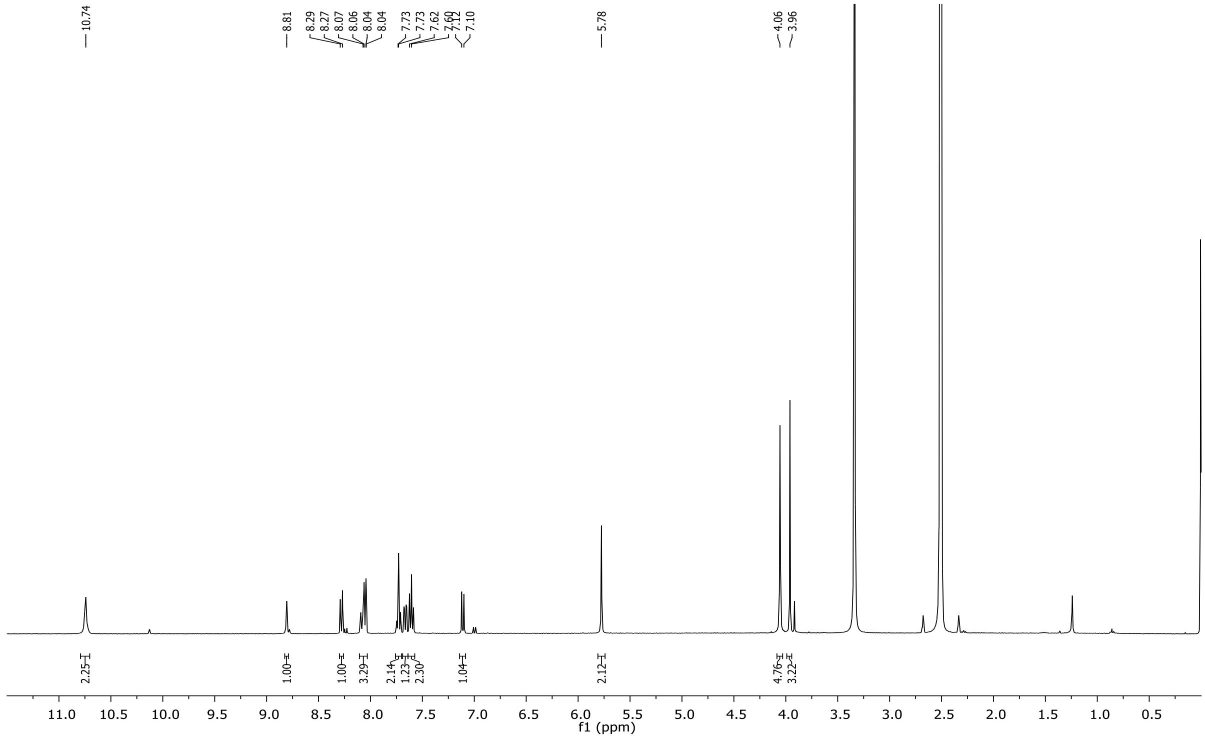

b)

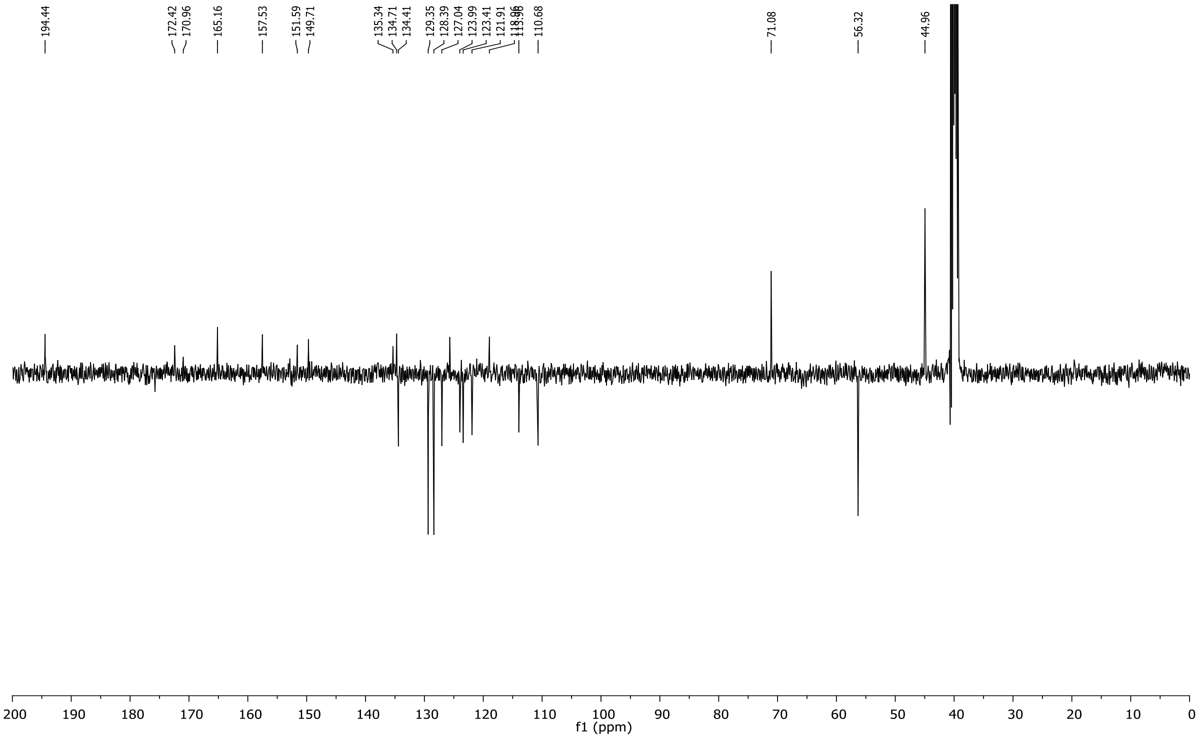

Fig. S58 a) ^{1}H NMR and b) ^{13}C NMR of compd. **35a**

a)

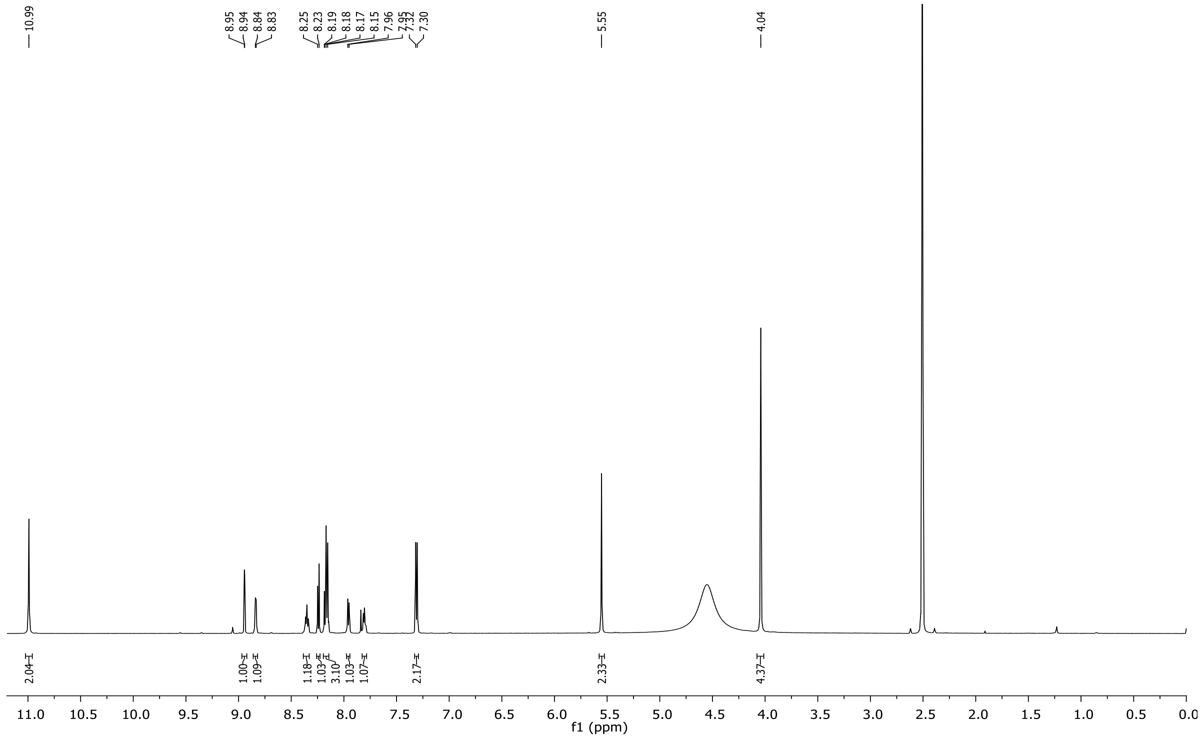

b)

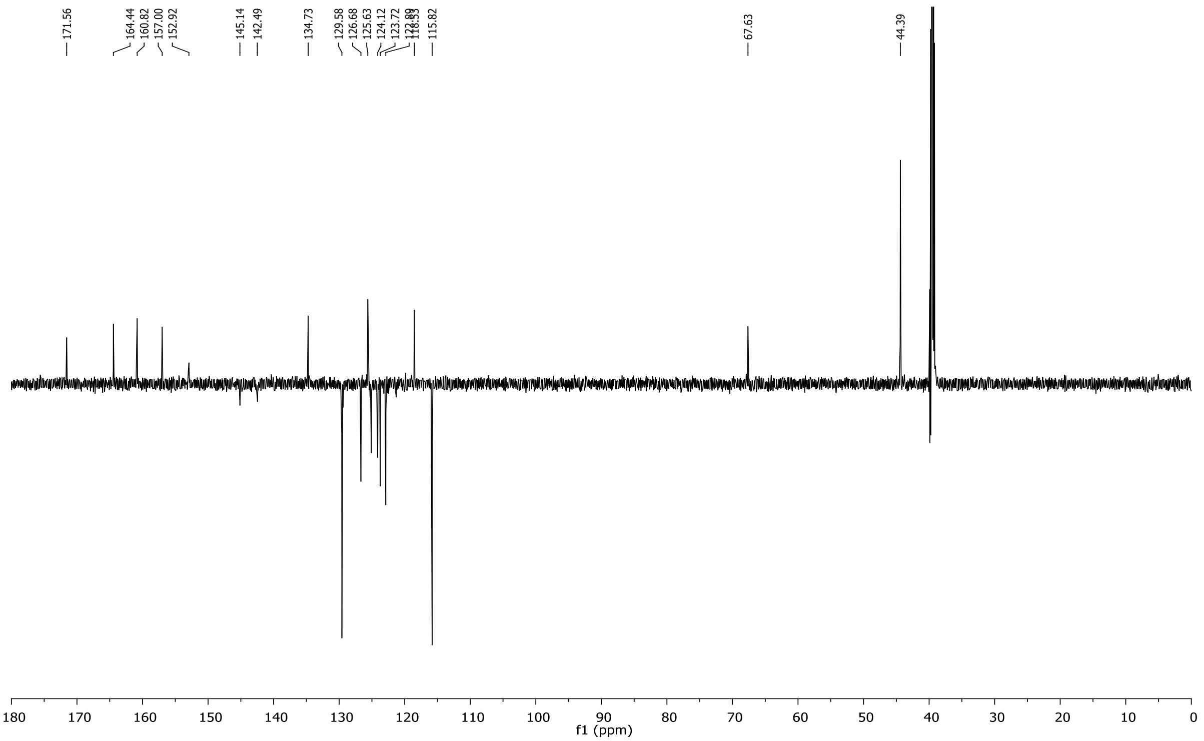

Fig. S59 a) ^{1}H NMR and b) ^{13}C NMR of compd. **35c**

a)

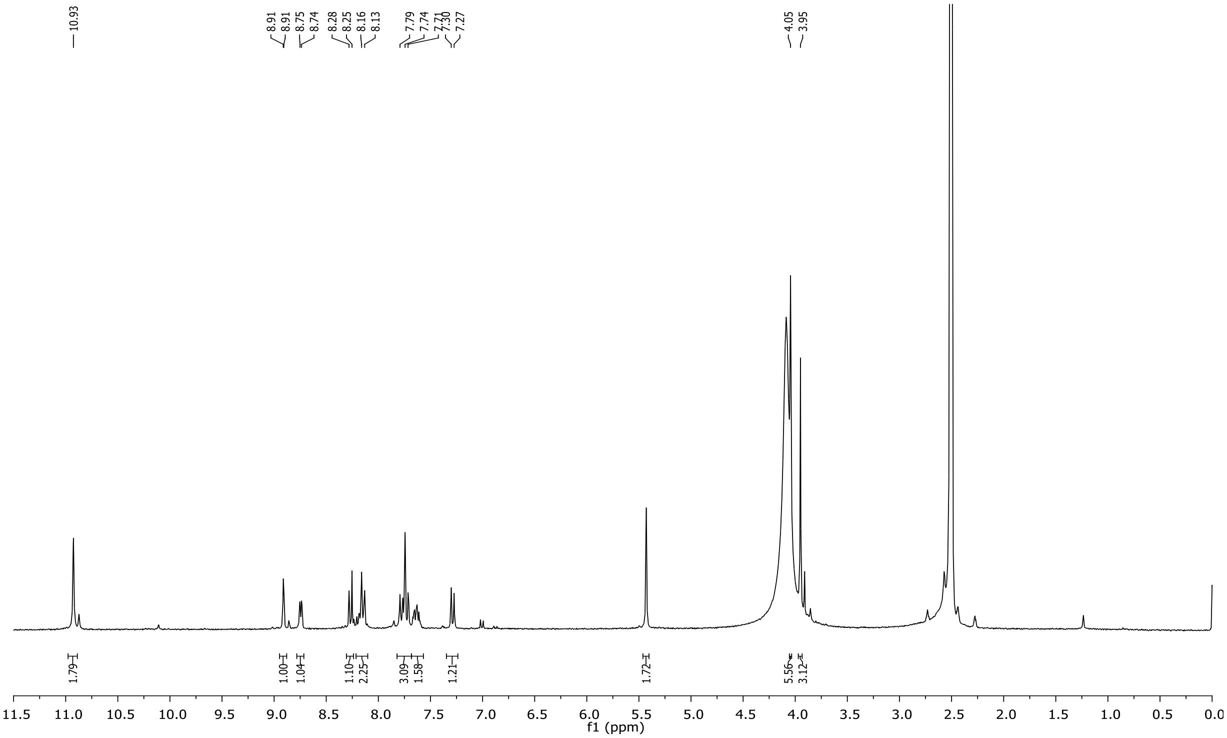

b)

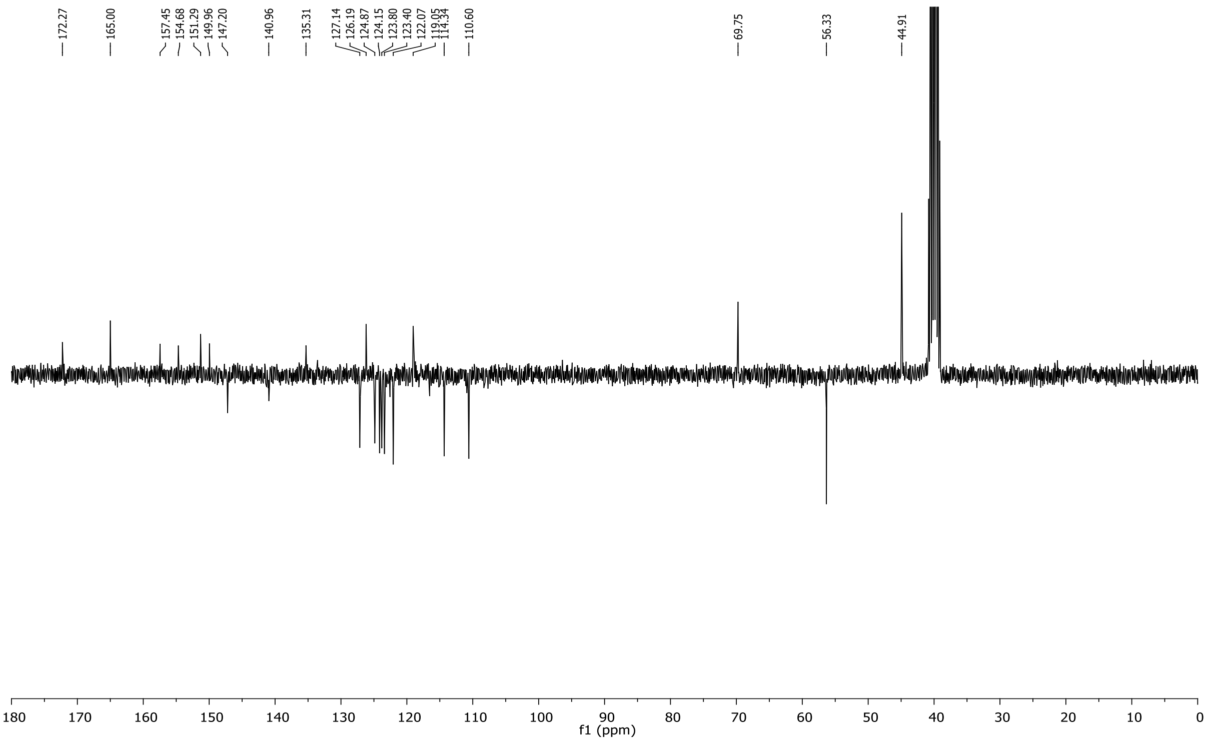

Fig. S60 a) ^{1}H NMR and b) ^{13}C NMR of compd. **36a**

a)

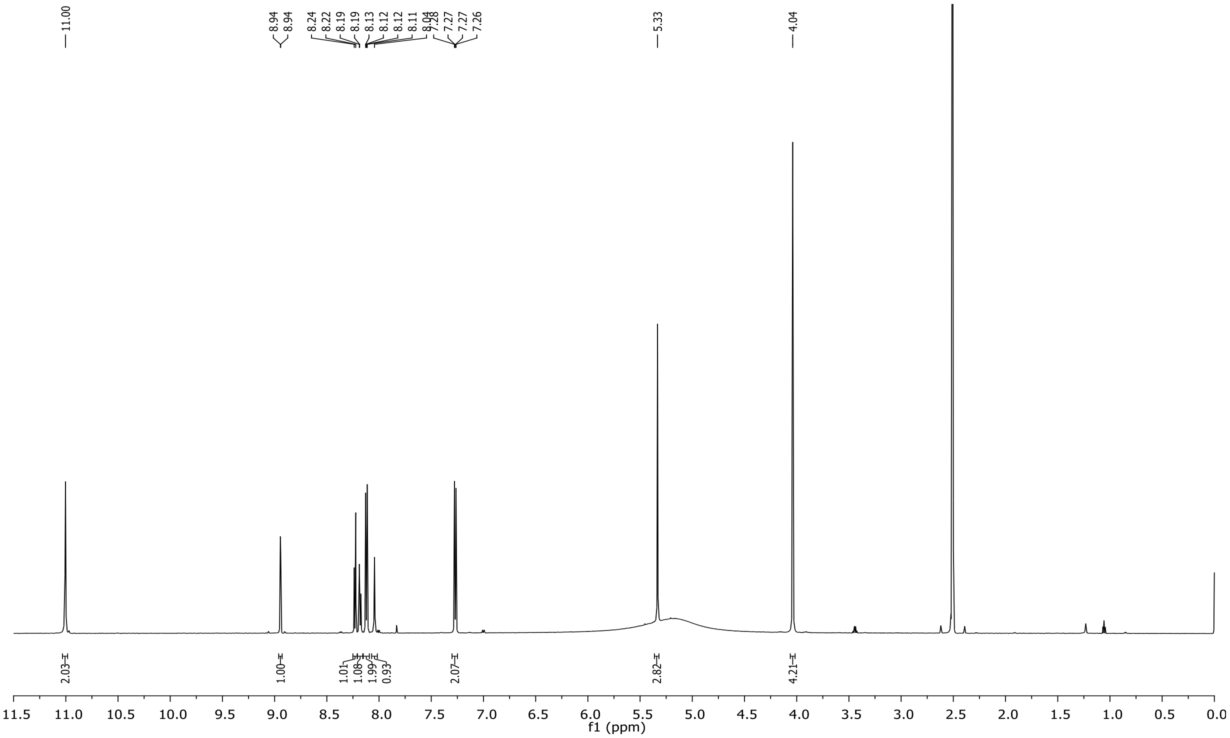

b)

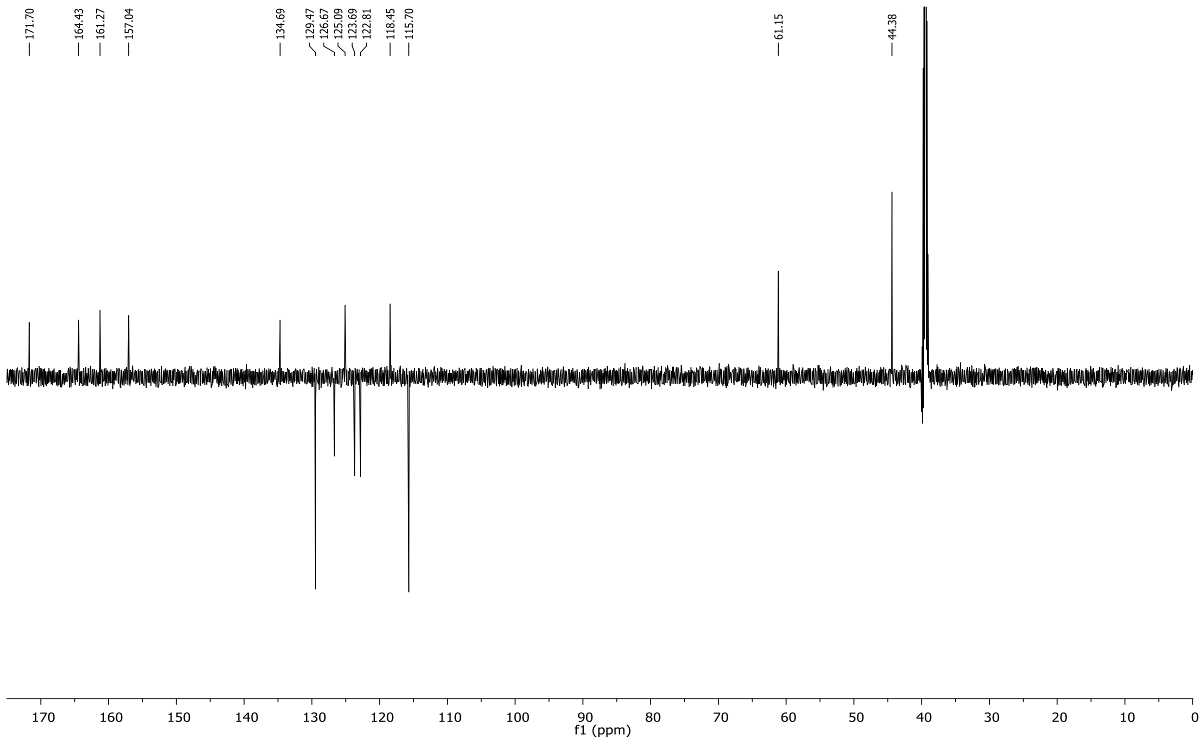

Fig. S61 a) ^{1}H NMR and b) ^{13}C NMR of compd. **36b**

a)

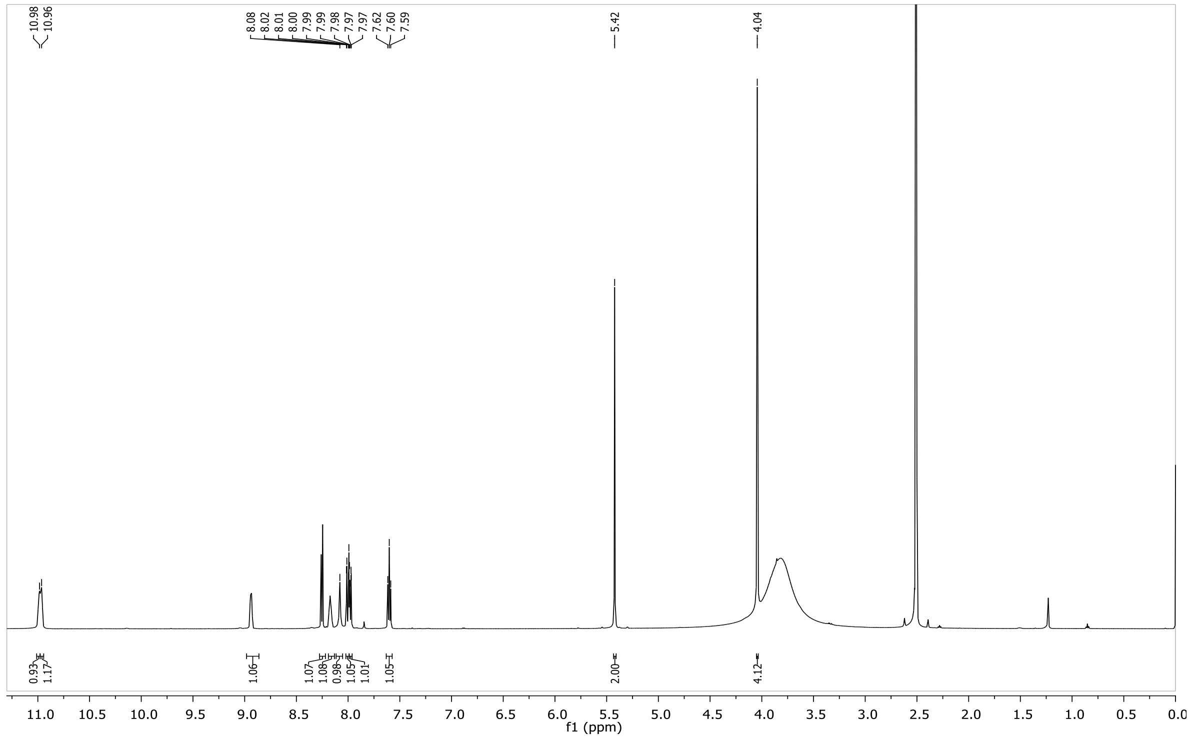

b)

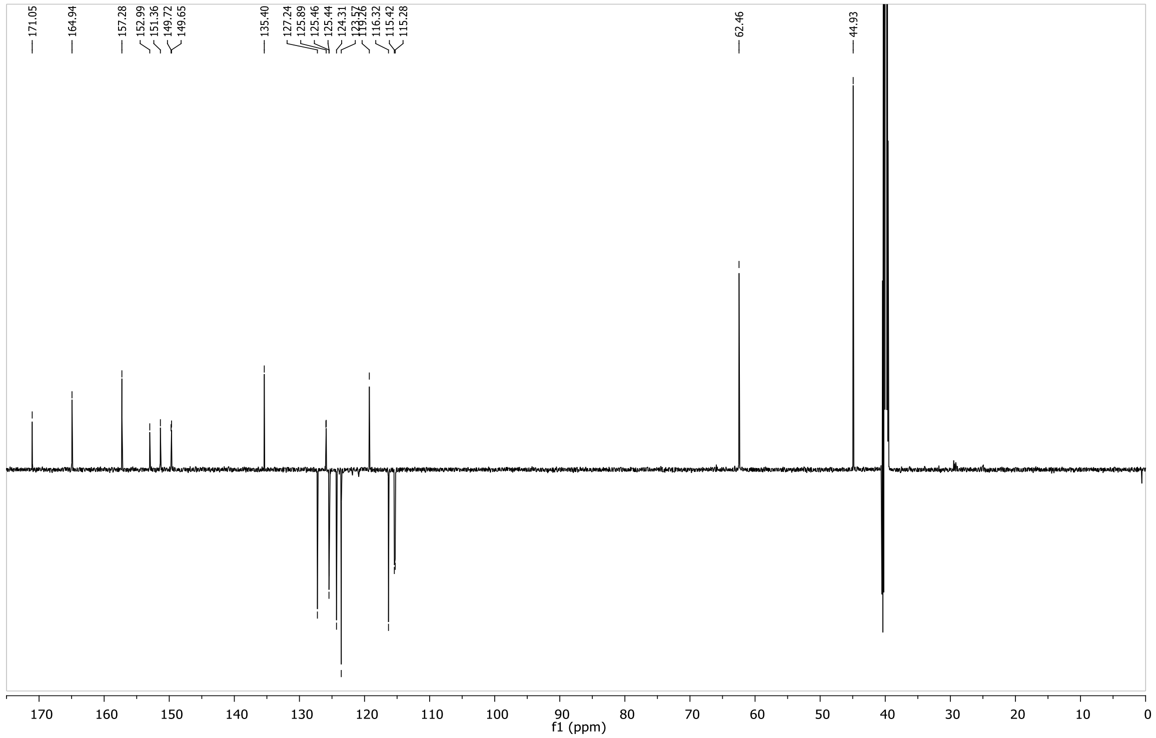

Fig. S62 a) ^{1}H NMR and b) ^{13}C NMR of compd. **36c**

a)

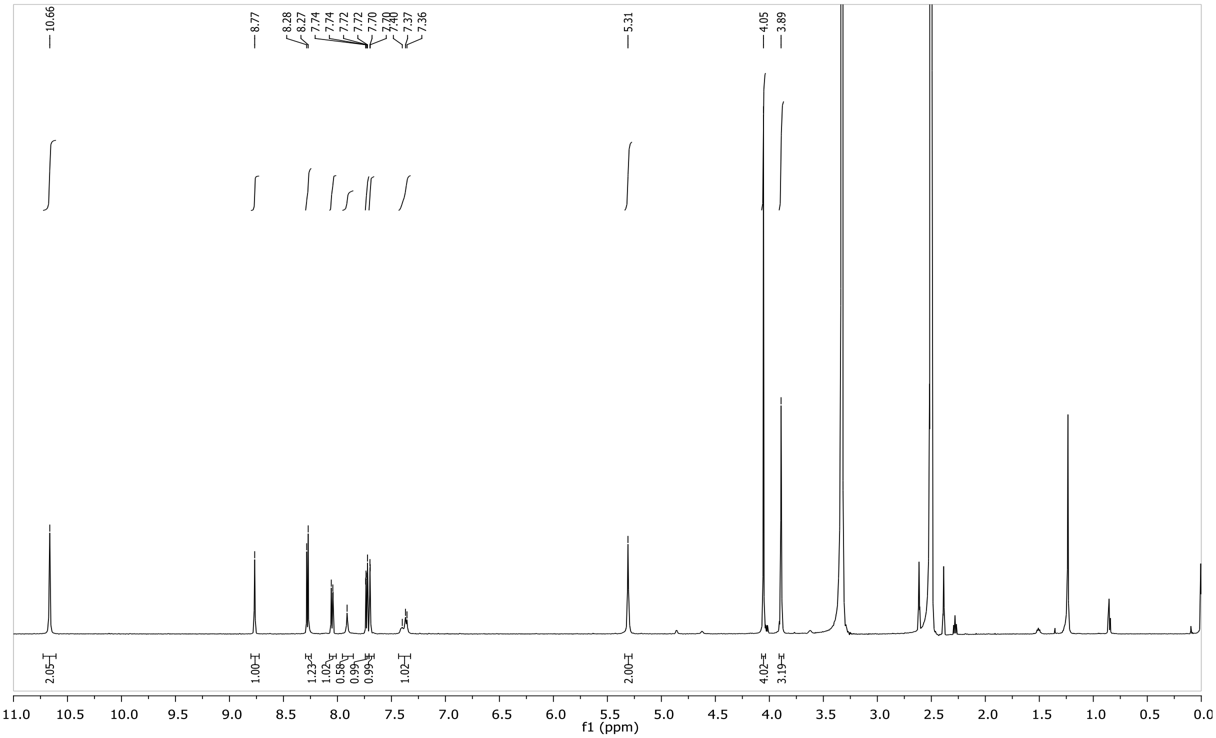

b)

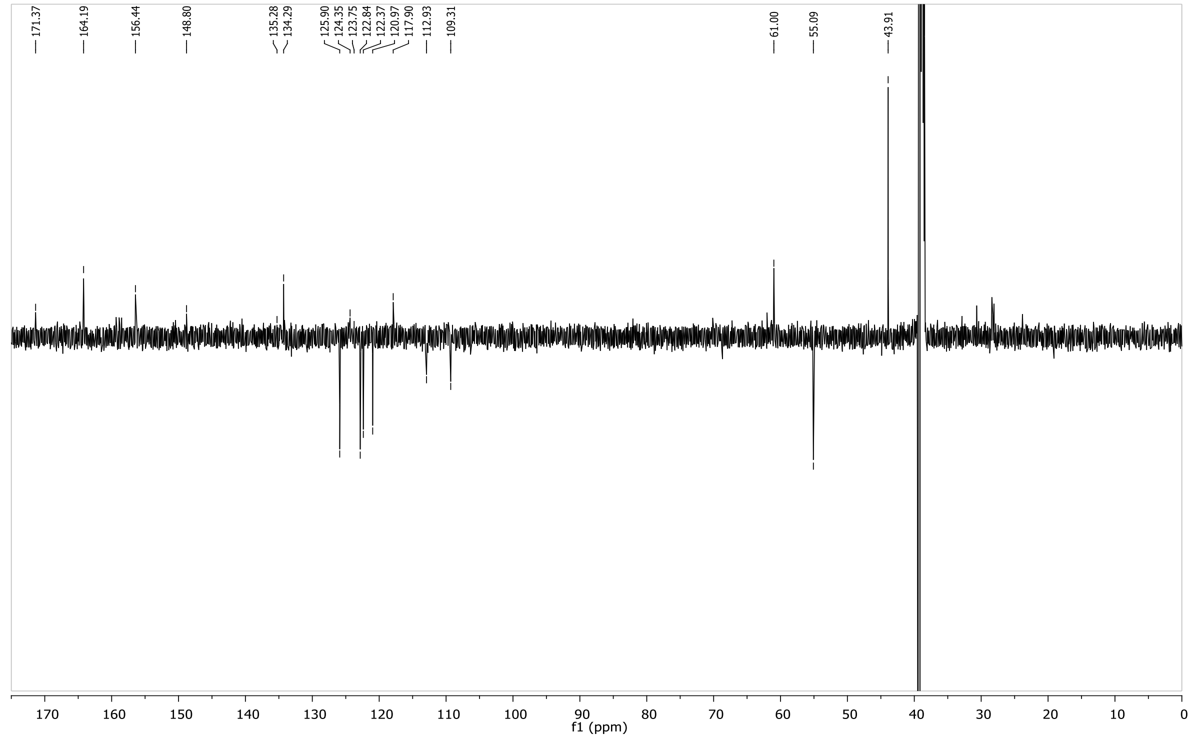

Fig. S63 a) ^{1}H NMR and b) ^{13}C NMR of compd. **37a**

a)

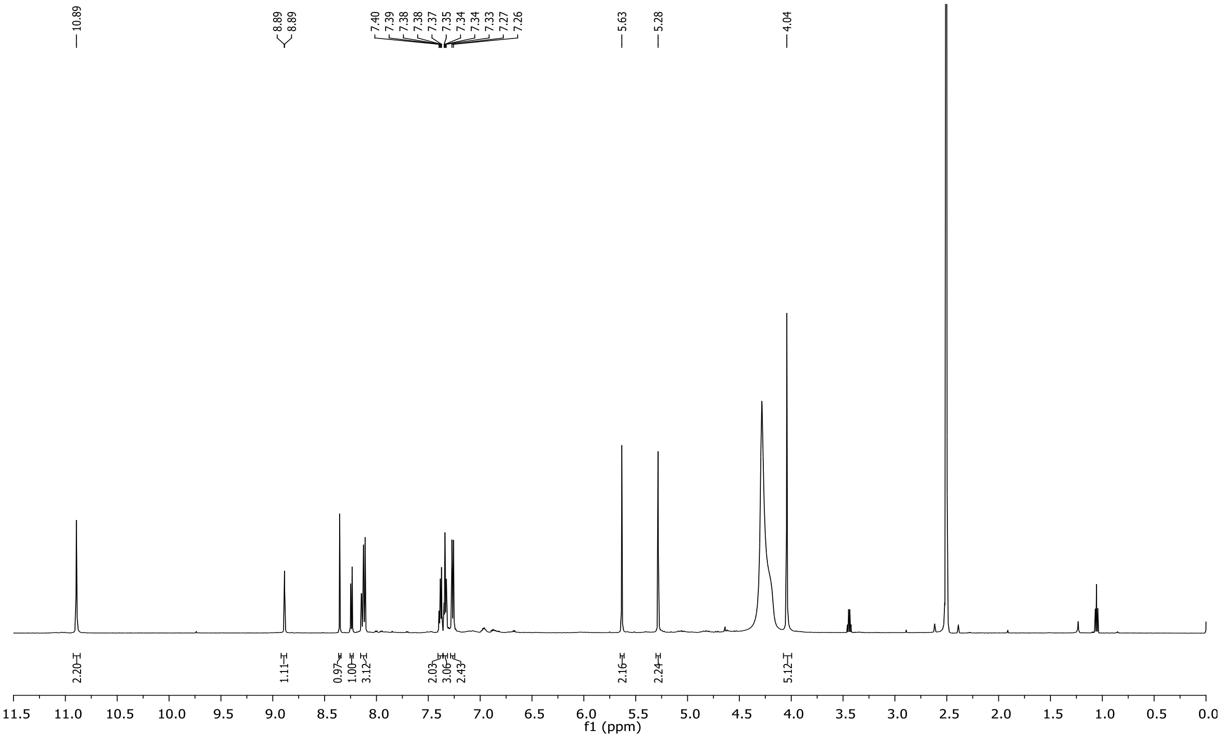

b)

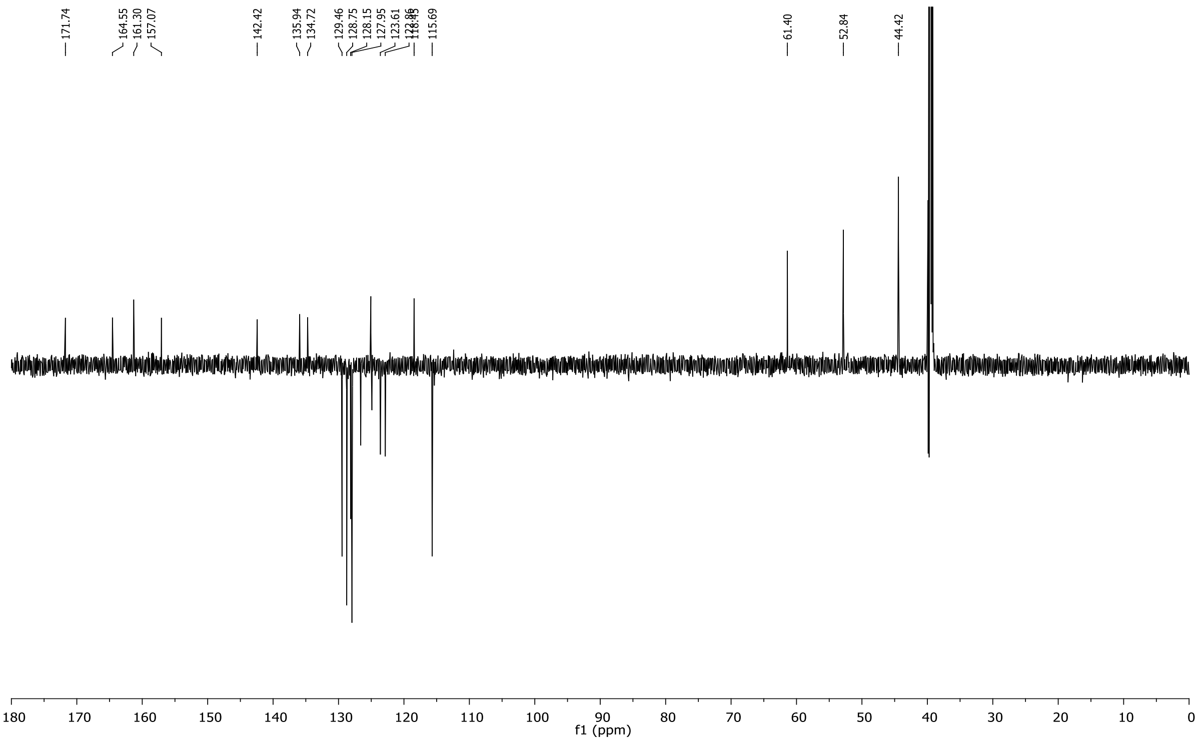

Fig. S64 a) ^{1}H NMR and b) ^{13}C NMR of compd. **37b**

a)

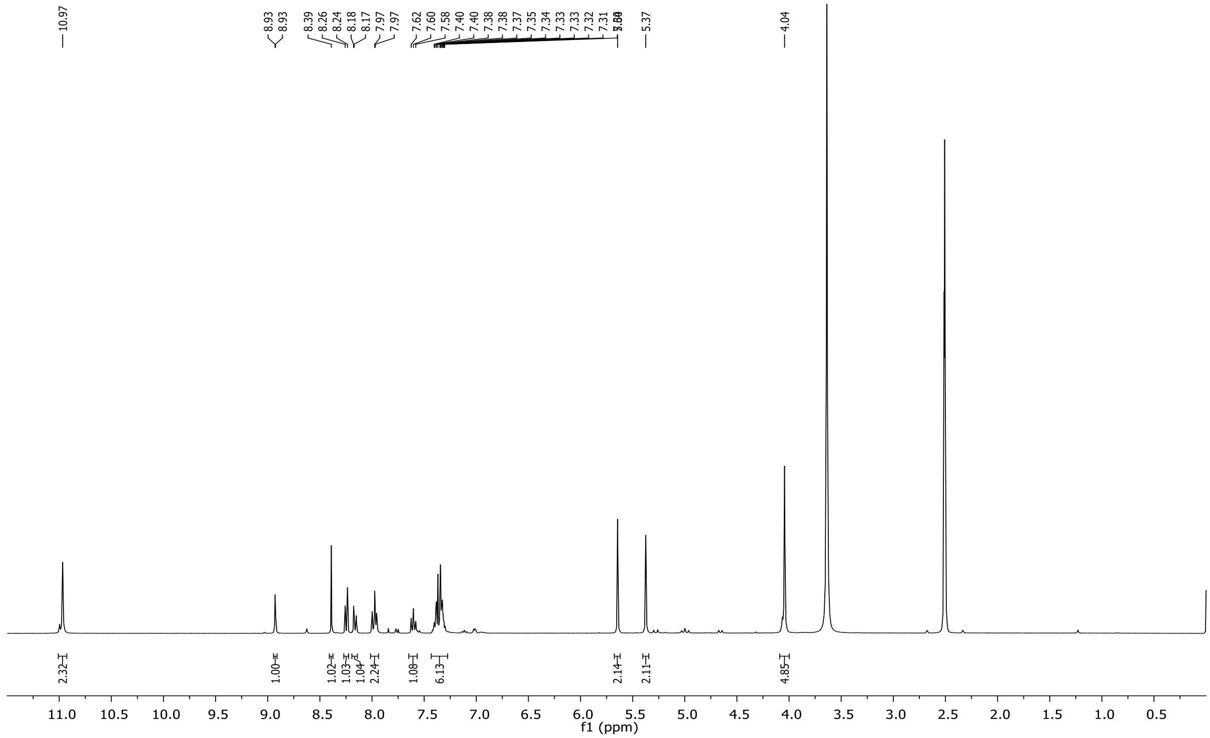

b)

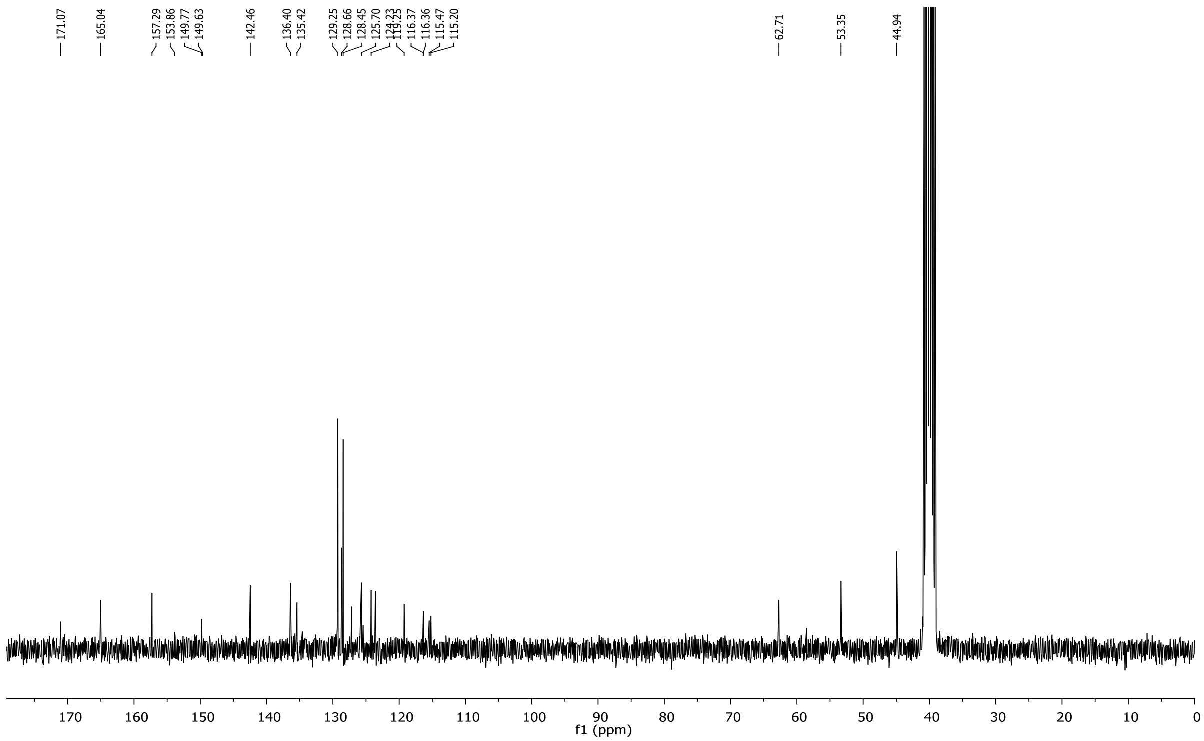

Fig. S65 a) ^{1}H NMR and b) ^{13}C NMR of compd. **37c**

a)

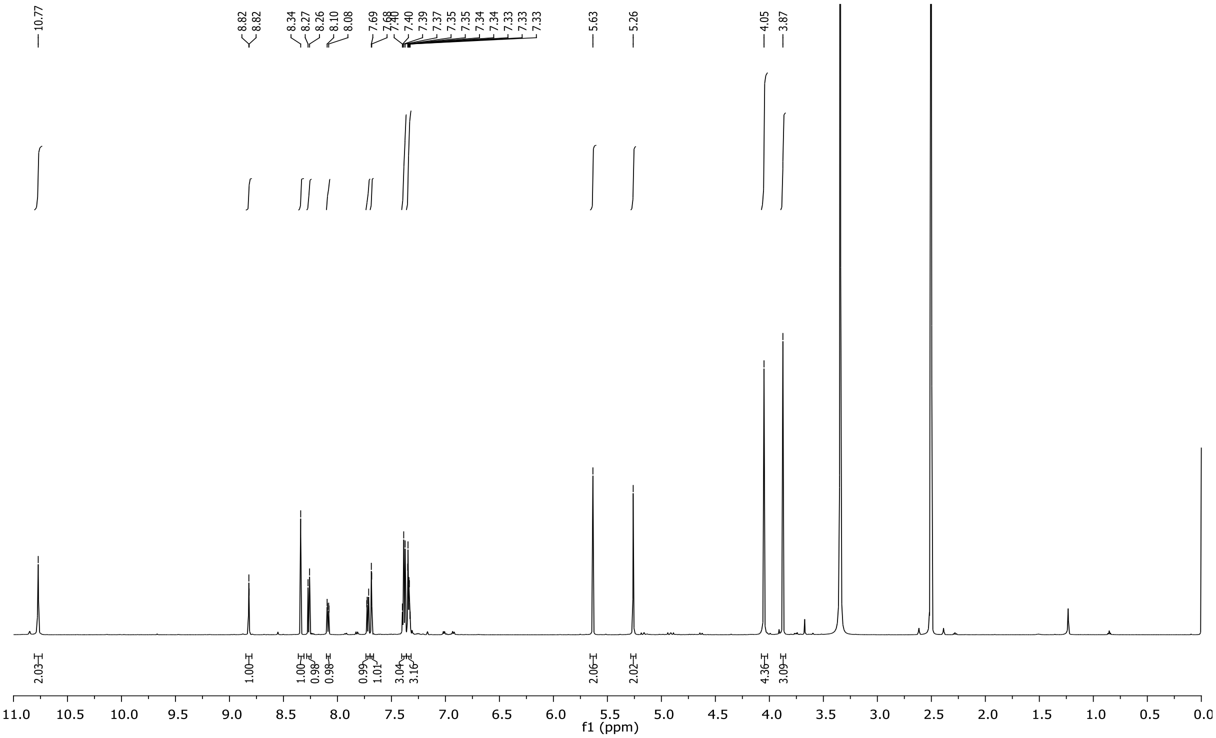

b)

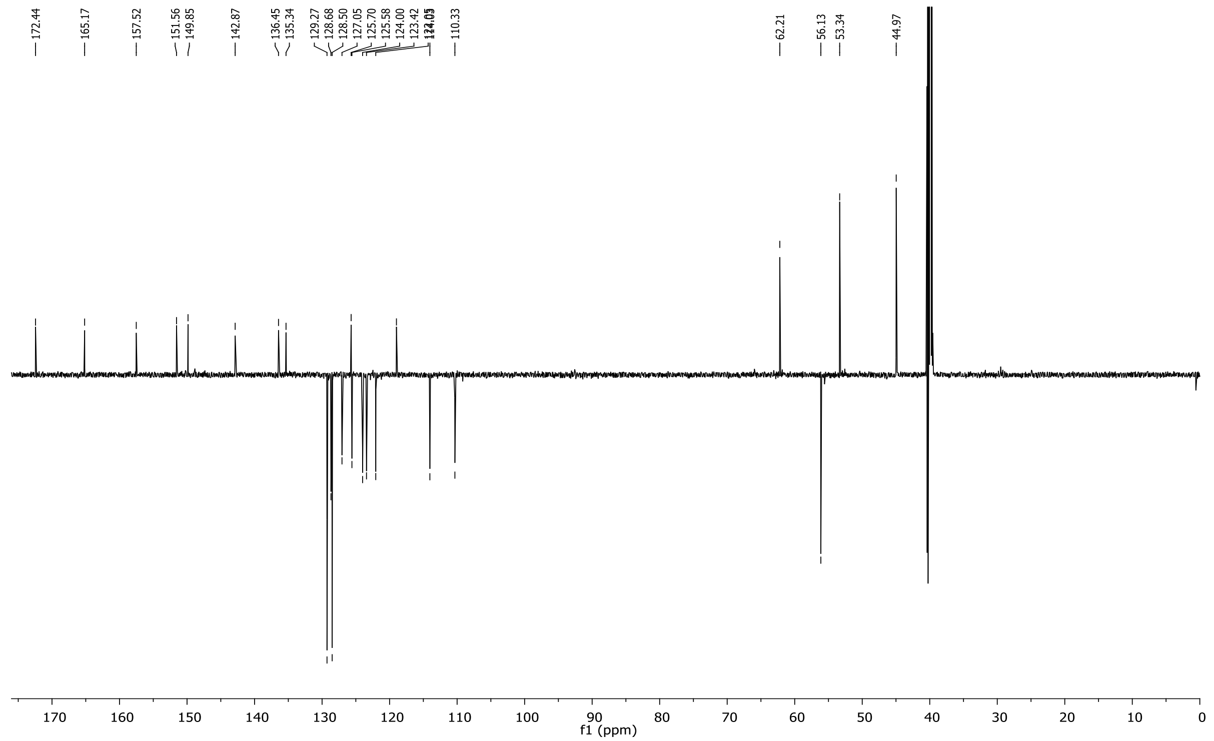

Supplement: Supplementary file 1 [file ijms-23-15843-s001.zip › ijms-2086430-supplementary.pdf]
